# Multivalent Fucosides Targeting β-Propeller Lectins from Lung Pathogens with Promising Anti-Adhesive Properties

**DOI:** 10.1021/acschembio.2c00708

**Published:** 2022-11-22

**Authors:** Margherita Duca, Diksha Haksar, Jacq van Neer, Dominique M.E. Thies-Weesie, Dania Martínez-Alarcón, Hans de Cock, Annabelle Varrot, Roland J. Pieters

**Affiliations:** † Department of Chemical Biology & Drug Discovery, Utrecht Institute for Pharmaceutical Sciences, Utrecht University, NL-3508 TB Utrecht, The Netherlands; ‡ Department of Biology, Utrecht University, Padualaan 8, 3584 CS Utrecht, The Netherlands; § Debye Institute for Nanomaterials Science, Utrecht University, Padualaan 8, 3584 CS Utrecht, The Netherlands; ∥ Univ. Grenoble Alpes, CNRS, CERMAV, 38000 Grenoble, France

## Abstract

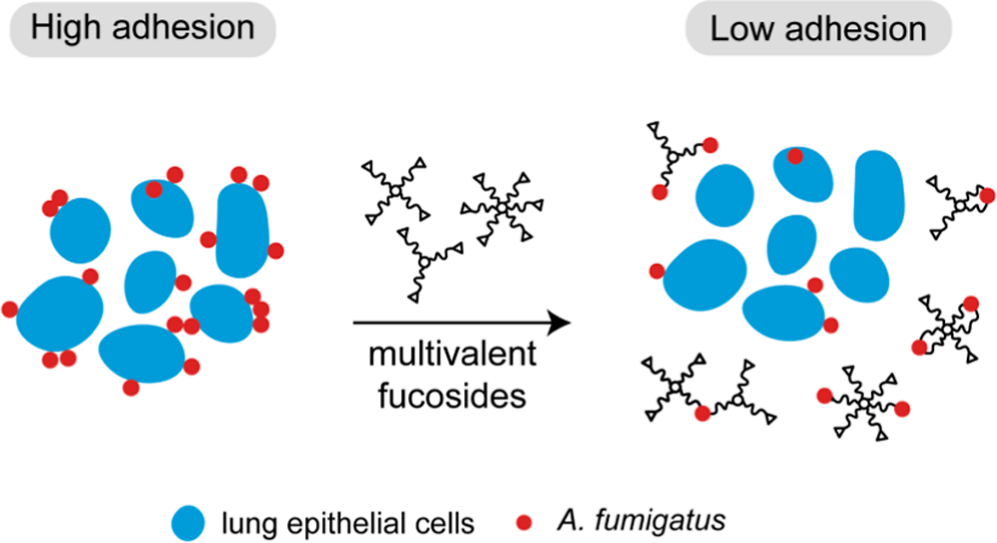

Fungal and bacterial pathogens causing lung infections often use lectins to mediate adhesion to glycoconjugates at the surface of host tissues. Given the rapid emergence of resistance to the treatments in current use, β-propeller lectins such as FleA from *Aspergillus fumigatus*, SapL1 from *Scedosporium apiospermum,* and BambL from *Burkholderia ambifaria* have become appealing targets for the design of anti-adhesive agents. In search of novel and cheap anti-infectious agents, we synthesized multivalent compounds that can display up to 20 units of fucose, the natural ligand. We obtained nanomolar inhibitors that are several orders of magnitude stronger than their monovalent analogue according to several biophysical techniques (i.e., fluorescence polarization, isothermal titration calorimetry, and bio-layer interferometry). The reason for high affinity might be attributed to a strong aggregating mechanism, which was examined by analytical ultracentrifugation. Notably, the fucosylated inhibitors reduced the adhesion of *A. fumigatus* spores to lung epithelial cells when administered 1 h before or after the infection of human lung epithelial cells. For this reason, we propose them as promising anti-adhesive drugs for the prevention and treatment of aspergillosis and related microbial lung infections.

## Introduction

The discovery of antibiotics has probably been one of the greatest advances in medicine of the last century. Similarly, the clinical need of antifungal drugs has been increasing in the last few decades because the incidence of fungal diseases has greatly expanded.^1^ However, the rapid emergence of microbial strains resistant to available drugs, along with the increase of the number of immunocompromised people, demands the elaboration of novel defences to combat infections.^2,3^

Among all innovative alternatives to antimicrobials to tackle infections, the carbohydrate-based anti-adhesive approach is still considered underexplored.^4,5^ Anti-adhesive agents are designed to prohibit the long-lasting attachment of a microbe on the host cell’s surface, therefore preventing infection without generating selective pressure upon the pathogen.^6^ Microbes initially attach to the host cells via the action of surface lectins, which are carbohydrate-binding proteins which recognize specific glycan patterns on the cellular surface.^7^ Because of their role in immunity and infection, various lectins from pathogens have become appealing targets for the design of carbohydrate-based inhibitors.

Multivalency, which allows simultaneous low-affinity interactions between carbohydrate ligands and their receptors, plays a major role in adhesive events.^8^ It leads to avidity and also allows better selectivity. For these reasons, it is often applied to the design of synthetic glycoconjugates which can act as anti-infectious agents or vaccines. Various scaffolds with different valencies and spatial organizations (e.g., calixarenes, dendrimers, nanoparticles, cyclopeptides, and cyclodextrins)^9−14^ have been exploited to tailor the structure of the inhibitors on their targets, leading to strong binding by means of the chelate effect or statistical rebinding or combinations thereof.^8^

To contribute to the search of alternative treatments for microbial infections via multivalent drugs, this study targets three fucose-binding lectins belonging to the six-bladed β-propeller subfamily:^15^ FleA (also known as AFL), SapL1, and BambL. Such lectins are found on the surface of the highly resistant opportunistic lung pathogens, respectively, the microfungi *Aspergillus fumigatus* and *Scedosporium apiospermum*, and the bacterium *Burkholderia ambifaria*. The well-known *A. fumigatus* causes worldwide more than 8 million cases of allergic and chronic pulmonary infections each year. Strikingly, of the ∼350,000 patients who contract invasive aspergillosis annually, about 50% will die even if treated with antifungals.^16,17^ Together with the emerging pathogen *S. apiospermum*, they are the most prevalent airborne filamentous fungi isolated from the lungs of cystic fibrosis patients and both present resistance to many antifungal classes. The Gram-negative bacterium *B. ambifaria* is a similarly harmful agent: as one of the virulent species of the *Burkholderia cenocepacia* complex, it is responsible for a respiratory infection known as “Cepacia syndrome”.^18^ Its high resistance to many antibiotics and its ability to form biofilms results in frequent cross-infections and nosocomial outbreaks among immunosuppressed individuals and cystic fibrosis patients.^19−21^

**Figure 1:**
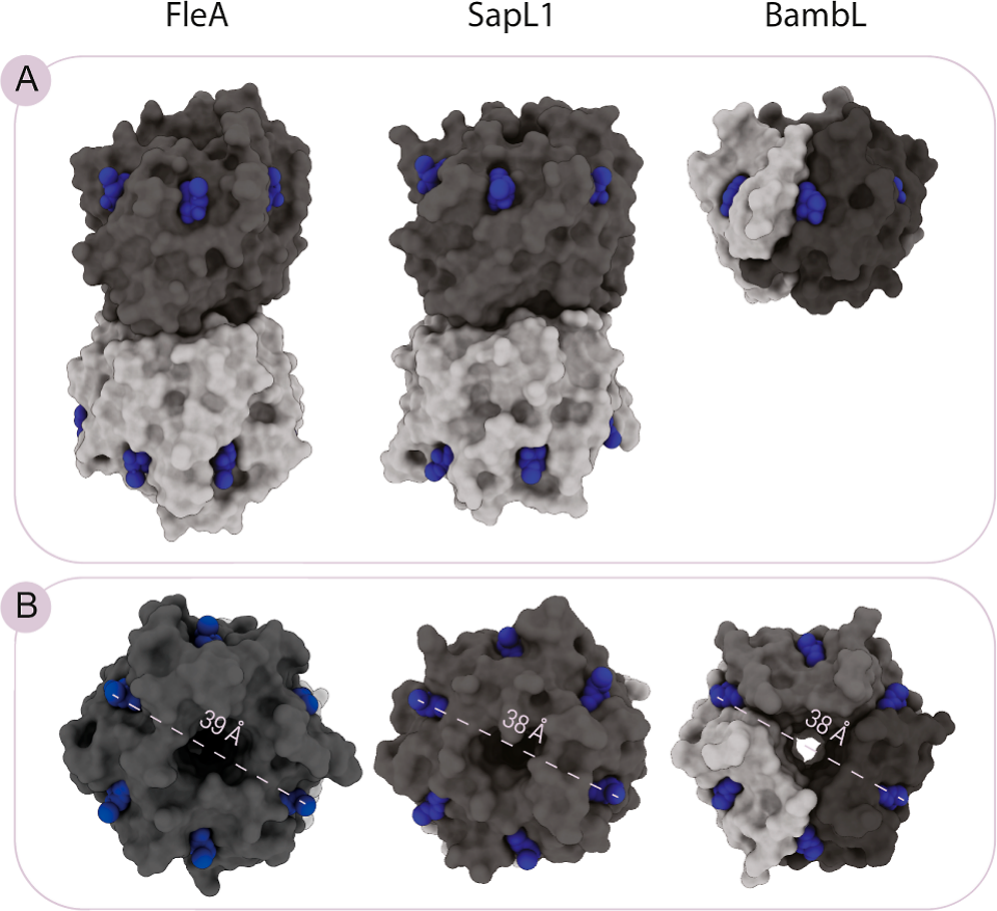
Overall structure of the targeted lectins: FleA, SapL1, and BambL (PDB codes 4AGI, 6TRV, and 3WZ2, respectively). (A) Side view. (B) Top view. Different monomers of the same protein are depicted in different shades of gray. The fucoside ligand is colored in blue and occupies each binding site. The longest distance between the opposite binding sites is indicated with a dashed line.

Besides their similarities as the cause of lung diseases, these pathogens present, in particular, one lectin that is comparable in its tertiary structure and specificity for fucosides. FleA, SapL1, and BambL are β-propeller lectins constituted by antiparallel beta-sheets which organize in six blades around a central funnel-like pore to generate a donut-shaped assembly.^15^ Structural characterization by X-ray crystallography showed that SapL1 and FleA share a high similarity in their tertiary and quaternary structures, featuring a homodimeric assembly in which each monomer displays six fucose binding sites at the interface between blades.^22,23^ BambL, instead, is formed by the trimerization of a shorter peptidic chain.^24^ Interestingly, such an arrangement leads to a hexavalent lectin with one binding surface, while the fungal homologues FleA and SapL1 are dodecavalent with two opposite binding faces (Figure 1).

Thanks to this resemblance, it is logical to design multivalent anti-adhesives which can be used for their inhibition in a broad-spectrum fashion. To explore the effect of dimension and valency on the affinity for their targets, these structural features were combined in the synthesis of a set of compounds. Such compounds can display up to six fucoside units, the maximum number of adjacent ligands that can be bound on a protein binding face. As a comparison, we additionally prepared a fucose-conjugate polymer of high valency, designed without a rigorous architecture. The binding properties of the resulting multivalent fucosides were assessed by various techniques (i.e., fluorescence polarization: FP, isothermal titration calorimetry: ITC, and bio-layer interferometry: BLI) to explore their potential applicability as anti-adhesive agents while subsequently demonstrating their effect on the attachment of fungal spores to human lung epithelial cells.

## Results and Discussion

### Synthesis

The analysis of the interactions of FleA, SapL1, and BambL with blood group epitopes by glycan array displayed their preference for short, non-branched α-fucosylated oligosaccharides.^22−24^ The weak affinity for α-methyl fucose, with K_d_s in the range 1–200 μM, was in some cases ameliorated by its presentation in a disaccharide; especially the α1,3/4 appeared as the preferred fucose linkage and common to all three lectins. For example, the *K*_d_ of binding for FleA is less than half when passing from the monosaccharide L-Fuc (209 μM) to the disaccharide αFuc(1–4)GlcNAc (63 μM) in an SPR experiment.^25^ Besides these disaccharides, efforts to prepare simplified monovalent ligands with enhanced potency have yielded compounds with a *K*_d_ of 19 μM.^26^ While the ultimate multivalent inhibitor will be composed of the optimal monovalent ligand combined with the optimal scaffold;^27^ at this stage, α-l-fucose was chosen as a suitable monovalent ligand for scaffold explorations.

**Scheme 1:**
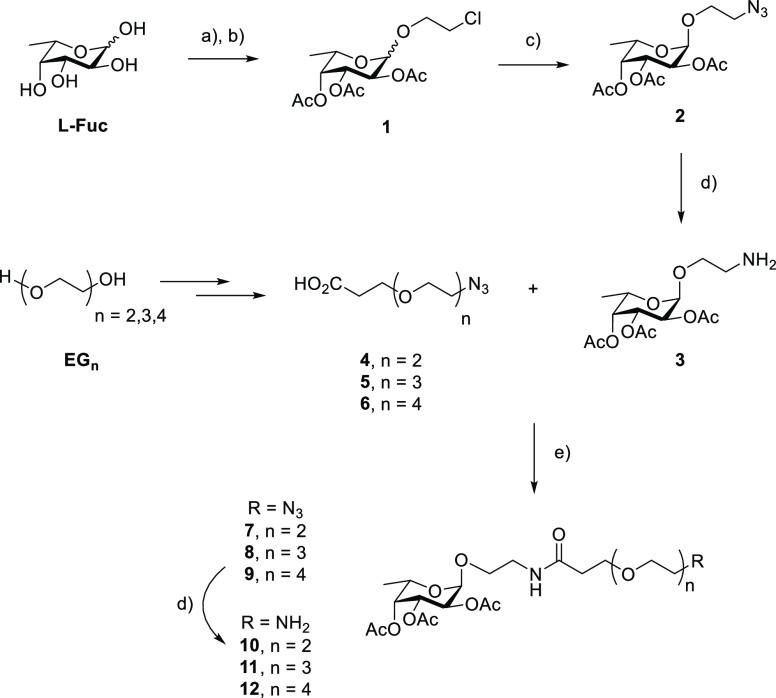
Synthesis of the Elongated Ligands **10–12** Reagents and conditions: (a) 2-chloroethanol, Amberlite IR-120 H^+^, reflux; (b) Ac_2_O, Py (68% over 2 steps); (c) NaN_3_, TBAI, DMF, 90 °C (48% α); (d) SnCl_2_, HCl aq., MeOH (99%); and (e) HATU, DIPEA, DCM (34–53%).

Fisher glycosylation of the monosaccharide in 2-chloroethanol, followed by peracetylation, was employed to obtain the corresponding fucoside **1** in a 7:3 α/β ratio (Scheme 1). Similarly to what was reported by Wang et al.,^28^ the reaction of **1** with NaN_3_ afforded the pure azido fucoside **2** after chromatography. Reduction to the corresponding amine **3** was performed with SnCl_2_^29^ because acetyl migration was observed with hydrogenation or the Staudinger reaction. We selected a flexible polyethylene glycol (PEG) spacer, tunable in length, to connect the central aromatic core with the peripheral monosaccharides. Although rigid spacers can achieve more effective binding due to lower entropic loss upon chelation, their design is more challenging because it must have a perfect fit.^30^ Flexible linkers such as PEGs, on the other hand, are advantageous for their plasticity and are suitable for broad-spectrum inhibition of lectins and cases of high valency. To optimize the structure for a possible chelating mechanism, besides, for example, statistical rebinding, the dimensions of the spacer were tailored to span the furthest site distance of 39 Å. We calculated effective polymer length in terms of Flory radius^31^ and selected 2–4 units of PEG as the optimal size range. Following a four-step procedure described in the literature,^32,33^ building blocks **4**, **5**, and **6** were prepared in comparable yields (see the Supporting Information), ready to be combined with the same fucoside **3**. Peptide coupling reactions, employing HATU as coupling agent, afforded compounds **7**, **8**, and **9**. The subsequent reductions gave the spacer-elongated fucosides **10**, **11**, and **12** with the corresponding free amino functionality.

**Scheme 2:**
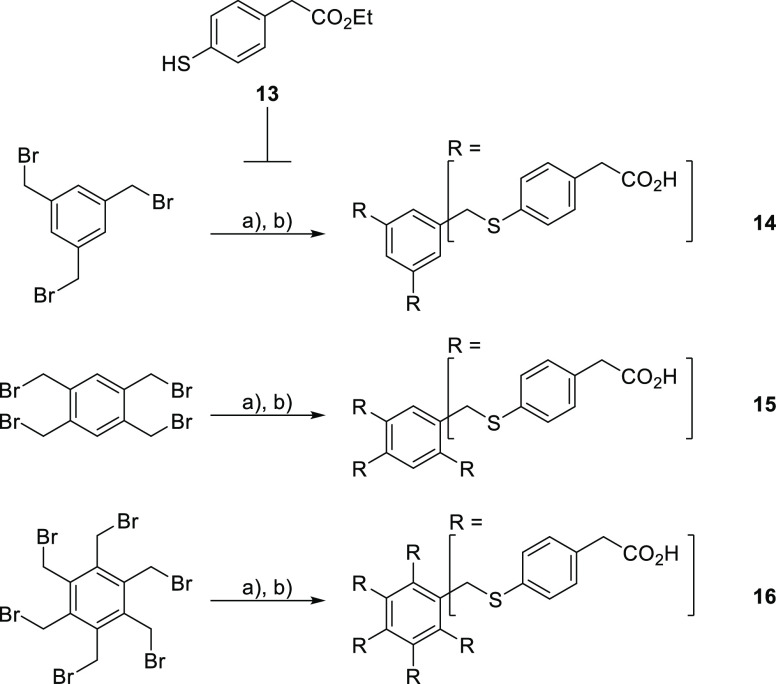
Synthesis of the Tri-, Tetra-, and Hexa-Valent Cores Reagents and conditions: (a) NaH, DMF; (b) THF/dioxane/NaOH 2 M 1:2:1 (41–87% over two steps).

To match the symmetry of the targeted proteins, a benzene ring was used as the central unit in the design of the tri- to six-armed dendrimers. Inspired by the promising inhibition properties of sulfurated asterisks reported by Sleiman et al. for the inhibition of Concavalin A,^34^ we designed similar core moieties starting from appropriately substituted benzenes. Commercial 4-mercapto-phenylacetic acid was first protected as the corresponding ethyl ester **13** (not shown) and then reacted with different benzyl bromides (Scheme 2). The subsequent hydrolysis liberated the carboxylic acids groups in **14**, **15**, and **16**.

**Scheme 3:**
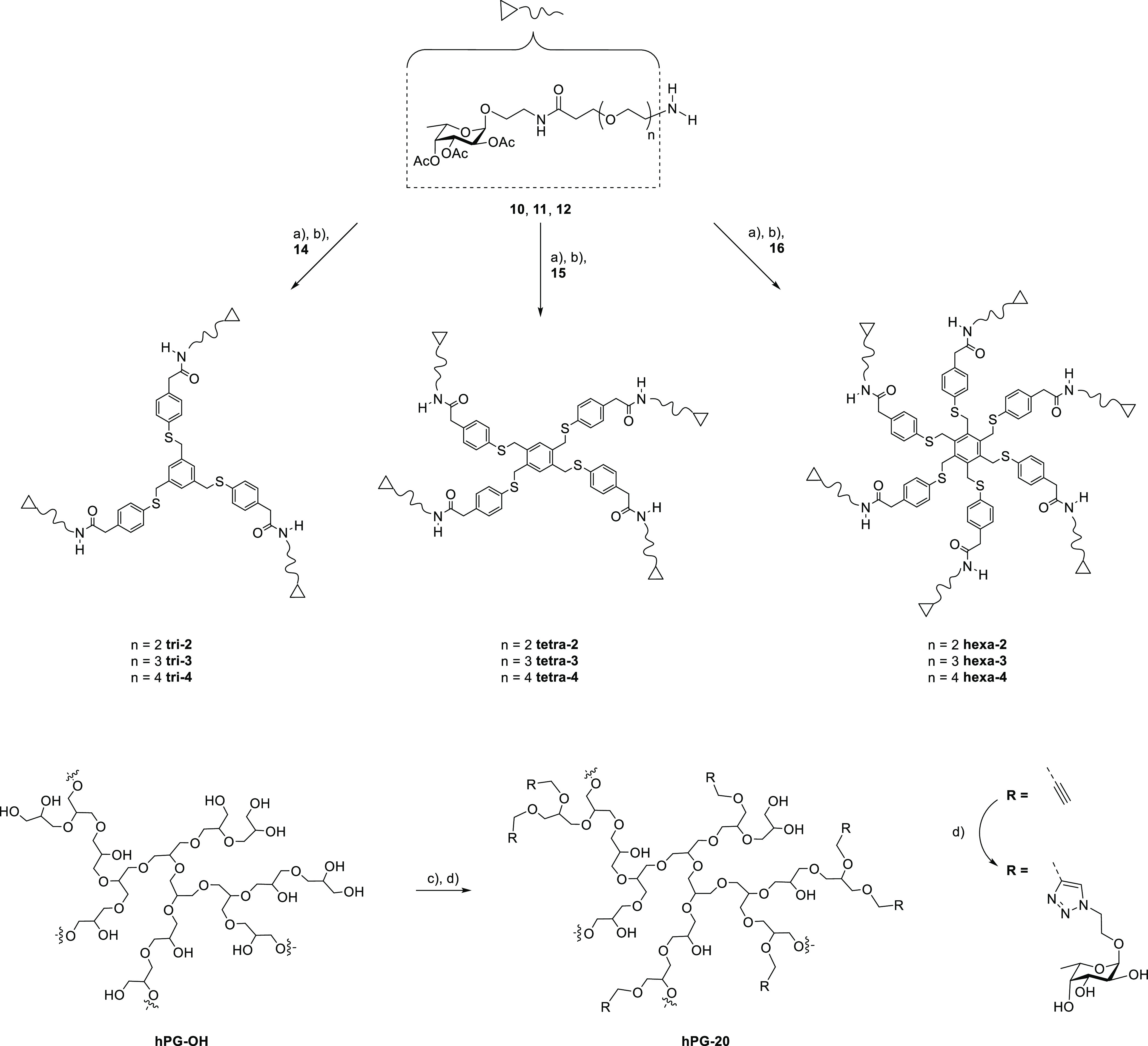
Final Steps in the Synthesis of Multivalent Fucosides Reagents and conditions: (a) HATU, DIPEA, DMF; (b) MeONa/MeOH; (c) NaH, KI, propargyl bromide; and (d) **2a**, CuSO_4_, Na ascorbate, 100 °C, MW. Products were obtained in yields ranging from 24 to 66% over two steps.

The final compounds were prepared as the result of all possible combinations between core valency and spacer length. To this end, each PEG-elongated ligand was multiplied into the final structure by peptide coupling with the cores **14**, **15**, and **16**. The subsequent deacetylation under Zemplén conditions afforded all the final products (Scheme 3, top). Moderate yields are due to incomplete coupling reaction and to difficulties in purification of the final compounds, which was done by silica gel chromatography and preparative HPLC. Considering that three to six couplings are happening in the same reaction step, the results were nevertheless satisfactory and we did not focus on the further optimization of this step.

To explore the limits of valency and take a less strict approach to the chelate-driven design, we expanded the PEG spacer to the level of polymer chemistry in the design of a hyperbranched polyglycerol (hPG)-fucose conjugate. Being favorably stable, biocompatible, and cheap,^35^ hPG nanoparticles have already been used in our group to successfully inhibit the cholera toxin B subunit^36^ and the Shiga toxin,^37^ both systems with a high number of binding sites. We used the same synthetic strategy as recently reported^36^ and added **hPG-20** to the repertoire of our multivalent compounds (Scheme 3, bottom). More in detail, **hPG-OH** was prepared according to a reported procedure^38^ and then derivatized with propargyl bromide. According to NMR, the level of functionalization obtained was 16%, which corresponds to about 20 propargyl end groups per molecule. We finally used copper-catalyzed alkyne azide cycloaddition (CuAAC) to conjugate the polymer to the deacetylated version of fucoside **2** (**2a**, structure and synthesis in the Supporting Information). IR confirmed the absence of alkyne and azide stretching peaks, indicating the complete conversion of hPG-propargyl into the desired product **hPG-20**.

### Evaluation of Binding Affinities

In our search for novel inhibitors of β-propeller lectins, we employed several biophysical techniques to assess the binding properties of the synthesized compounds. In the expression of FleA and Sapl1, we took advantage of a high-yielding purification method involving a *N*-terminal tag presenting 6xHis, removed afterward with the Tobacco Etch Virus (TEV) protease to get native protein, as described.^23,39^ Our attempts to do the same for BambL using the vector pProEX-Htb failed the tag cleavage, presumably due to inaccessibility of the TEV cleavage site. Because of this, the recombinant protein was obtained from the expression of pET25-*bambL* plasmid, as reported before.^24^

With the targeted lectins in hand, we could screen the inhibitory properties of the synthesized compounds by a competitive binding assay based on fluorescence polarization (FP). In the assay, all multivalent compounds showed the ability to displace a fluorescent fucoside probe^40^ from the binding site. In general, results confirmed the power of multivalency as IC_50_ values improved from the micromolar range for the monosaccharide reference to the nanomolar range for the multivalent counterparts (Table 1).

**Table 1:** Inhibitory Potencies Toward FleA, SapL1, and BambL, as Obtained from a Competitive FP Assay^a^

	IC_50_ (nM) ± SD		
Compd	FleA	SapL1	BambL
**α-Me-Fuc**	111,000 ± 14,000	24,000 ± 11,000	1400 ± 200
**tri-2**	794 ± 29	147 ± 10	43 ± 1
**tetra-2**	619 ± 15	69 ± 3	43 ± 2
**hexa-2**	144 ± 4	101 ± 7	34 ± 1
**tri-3**	517 ± 47	82 ± 6	44 ± 1
**tetra-3**	173 ± 14	60 ± 5	26 ± 1
**hexa-3**	137 ± 1	89 ± 1	28 ± 3
**tri-4**	234 ± 5	79 ± 1	39 ± 2
**tetra-4**	160 ± 10	69 ± 3	26 ± 1
**hexa-4**	148 ± 6	43 ± 6	31 ± 3
**hPG-20**	24 ± 2	16 ± 1	9 ± 0

a IC_50_ values are calculated as an average over two independent experiments. Exact concentrations of species in solution for each experiment can be found in the Supporting Information.

Increasing both spacer length and valency contributed to the improved binding to FleA and SapL1. A beneficial effect of the valency was also evident from the results of **hPG-20**, as the most potent binder. For the fungal lectins, the impact of spacer length was clear: glycodendrimers with PEG length *n* = 4 were the most potent. The bacterial lectin BambL, instead, did not show striking preferences. Surprisingly, all multivalent fucosides inhibited the bacterial lectin with almost equal potency and none of the valency nor dimension appears to be significantly privileged. The binding pockets of BambL are more open with a shallow groove leading to the central funnel and symmetrical; therefore, we speculate that this might play a role in easing the accessibility of the compounds into the binding sites and allow the inhibitors to achieve strong binding with only few units of ligand bound. The situation for FleA (and its homologous SapL1) might be more complex because the protein is known to have non-equal binding sites and some binding sites are more buried with surface loops blocking access to the central funnel (Figure 1).^25^

Due to the time-consuming measurements and the high quantity of protein necessary in some techniques, we selected only the four inhibitors with the largest size (compounds with PEG_*n*=4_ and **hPG-20**) for further biophysical analysis. More in-depth studies were continued by isothermal titration calorimetry (ITC) and bio-layer interferometry (BLI), which confirmed the trends previously seen by FP (Table 2). In the ITC experiments, the fungal lectins bound their monovalent reference with a stoichiometry (*N*) value around 4, which is consistent with the number of high-affinity binding sites per monomer. With the value close to 6 obtained for BambL, the reference experiments are indicative of active recombinant proteins and correlate well with previous findings in the literature.^39,41^ The N values for multivalent compounds suggest that most of the binding sites are occupied. This calls for a likely mixed chelate-aggregative binding, whose precise architecture cannot be determined based solely on the compound/protein ratio.

**Table 2:** Binding Affinities to FleA, SapL1, and BambL, Measured by ITC and BLI at 25 °C

	FleA				SapL1				BambL			
	ITC			BLI	ITC			BLI	ITC			BLI
compd	*K*_d_ (nM)	β(/v)^a^	*N*^b^	*K*_obs_ (nM)	*K*_d_ (nM)	β(/v)^a^	*N*^b^	*K*_obs_ (nM)	*K*_d_ (nM)	β(/v)^a^	*N*^b^	*K*_obs_ (nM)
**mono ref.**	97,600 ± 300		4.1		68,100 ± 4400		3.52		2140 ± 80		5.43	
**tri-4**	240 ± 24	407(136)	1.4	25.9 ± 0.3	59.3 ± 2.8	1148(383)	1.27	2.7 ± 0.1	3.7 ± 0.7	578(193)	2.91	0.8 ± 0.1
**tetra-4**	149 ± 17	655(164)	1.2	7.4 ± 0.1	59.9 ± 1.7	1137(284)	0.95	3.6 ± 0.1	3 ± 0.9	713(178)	1.47	1.1 ± 0.1
**hexa-4**	115 ± 4	849(142)	0.8	4.1 ± 0.1	24.9 ± 2.7	2735(456)	0.72	1.2 ± 0.2	4.7 ± 0.9	455(76)	1.05	1.6 ± 0.1
**hPG-20**	60 ± 4	1627(81)	0.4	3.5 ± 0.2	18.5 ± 2.2	3681(184)	0.32	0.8 ± 0.1	2.7 ± 0.1	793(40)	0.42	1.2 ± 0.1

a β affinity enhancement values are calculated as *K*_d,mono_/*K*_d,multi_. The affinity enhancement per single sugar is shown in brackets and calculated as β/v, where v stands for valency.

b For direct comparison of the results, the stoichiometry has been calculated as ratio of compound per protein containing six binding sites (FleA and SapL1 monomers or BambL trimer). The relative standard deviations were not higher than 15% and are reported in the Supporting Information, together with thermodynamic parameters for all experiments.

The fucosylated inhibitors showed great affinity enhancements (β factors) compared to the monovalent analogue, with the affinity improving hundreds of times per single sugar unit. Interestingly, the compound with the best affinity, **hPG-20**, is also the one with the worst β/v value. This can be explained considering the thermodynamic parameters of the interaction: although the compound has on average 20 ligand per molecule available for binding, Δ*H* of interaction is only ca. 8–5 times higher than for the monovalent fucoside **2a**, indicating that not all ligands are involved in the binding event (data shown in Supporting Information). Yet, there is some danger in trying to exactly interpret thermodynamic data because an aggregating behavior was observed by naked eye after each ITC titration. Multiple events can contribute additively to the final free energy of binding, even aggregation/precipitation, if associated with a release or consumption of heat. In this case, however, such irreversible aggregating behavior is not reported by calorimetry, at least not by the curve shape. Because the c value dictating the curve shape is constant during the experiment, we can safely assume that aggregation happens on a slower timescale than titration, as previously hypothesized by Toone et al. for the cross-linking of ConA via multivalent ligands.^42^ They also postulated enthalpy–entropy compensation as a hallmark for aggregation, where a gradual reduction of Δ*H* is accompanied by an increase of Δ*S* during the binding of successive saccharide units.^43^ Opposite to what was described, favorable enthalpic forces and unfavorable entropy costs increase gradually from the interaction with the lowest valency inhibitor (**tri-4**) to the highest (**hPG-20**). Still, the overall free energy of interaction (Δ*G*) has little variations.

Applying different techniques to study the mechanism of multivalent binding is always advisable to better understand the process at a molecular level.^44^ BLI is a biophysical technique where the target protein can be immobilized on the surface of the sensor, so problems related to aggregation should be eliminated. This technology has in fact been applied to the study of lectin-multivalent glycoconjugate interactions in a reliable, low-cost, and fast way.^45^ Our BLI experiments showed affinity values about 10-fold higher (lower *K*_obs_) than the values obtained by ITC in the inhibition of the fungal lectins, but the discrepancy was smaller for the bacterial one. A discrepancy of this nature is not too surprising considering that in the BLI experiment, one of the binding partners is immobilized, so its *K*_d_’s might be better labeled as *K*_obs_. Different techniques measure inherently different properties. It has been pointed out in a recent publication that drastically different outcomes can be obtained from solution to surface experimental approaches and that these variations are highly dependent on the accessibility of the binding motif.^46^ The arrangement of glycans and the linker used for immobilization have shown to influence affinity on the glycan array setting. Therefore, it is likely that also the arrangement of a protein on the surface can have a similar effect when the setting is reversed.^46,47^ The difference in affinities between ITC and BLI for the fungal versus the bacterial lectins may relate to their orientation on the surface. The proteins were immobilized to the surface of a streptavidin-coated sensor through biotinylation of their lysine residues. BambL has only one lysine per monomer, opposite to the binding surface, while FleA and SapL1 have several, some of which close to the binding sites. It is plausible that this would influence the binding of the lectins to the sensor and the exposure of their sites. Furthermore, the two fungal lectins possess two opposite faces where binding sites are located, so their opportunities to be cross-linked are higher than those for the half-sized BambL. Although we cannot prove it at this stage, we think that the presentation of BambL on the surface could partially impair the cross-linking ability of the multivalent compounds, while FleA and SapL1 have more possibilities for orientation and remain accessible to multiple modes of binding. In contrast, all binding sites and modalities are accessible in solution during ITC.

**Figure 2:**
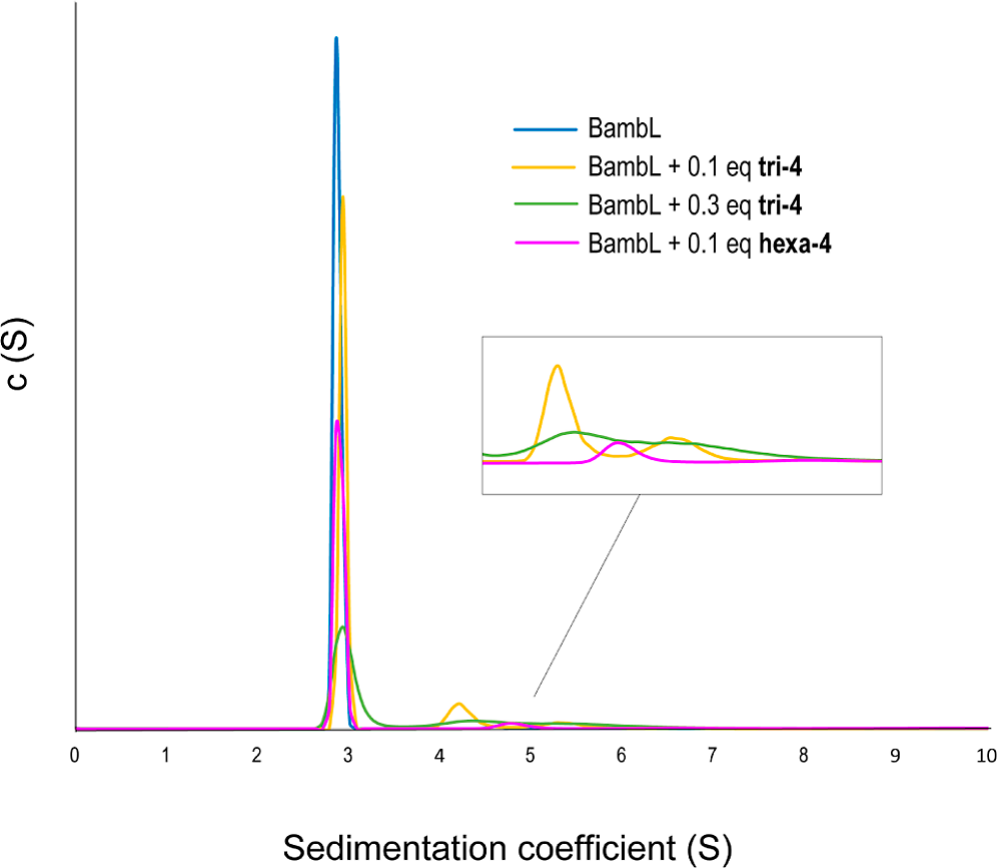
Distribution of the sedimentation coefficients for mixtures of BambL with different concentrations of **tri-4** and **hexa-4**.

To try and decipher the mechanism of cross-linking, we studied the formation of aggregates by analytical ultracentrifugation (AUC). Unfortunately, the analysis of the sedimentation profile when the proteins were in a 1:1 molar ratio with the simplest compound, **tri-4**, turned out to be inaccessible. The formation of insoluble aggregates of high mass was supported by a fast decrease of absorbance at λ 280 nm to below the limit of detection. Therefore, we decided to focus only on the AUC analysis of the smaller protein, BambL, in the presence of substoichiometric quantities of multivalent fucosides (Figure 2). A sedimentation coefficient of 2.96 S was found for the pure lectin in buffer solution, corresponding to a protein molecular weight of 27 kDa, which is very close to its real molecular weight of 28.1 kDa. For the mixtures, the comparison of absorbance intensity before and at the beginning of any centrifugation run showed that between 19 and 46% of the sample was already settled in insoluble complexes and only the remaining part of the sample in solution could be analyzed. Just 0.1 equiv of **tri-4** (0.15 equiv of fucose per protein) was enough to link two or three proteins intermolecularly, giving peaks at *S* = 4.37 and 5.48, corresponding to species of 46 and 64 kDa. These values are somewhat lower than the expected MWs but consistent with a greater friction of the real inhibitor–lectin complex compared to the theoretical sphere used as assumption in the calculation. Increasing the amount of **tri-4** by a factor of three resulted in a less defined oligomeric state, with the two peaks broadening, indicative of species in dynamic interchange with each other. An even more pronounced effect was observed when 0.1 equivalent of the higher valency **hexa-4** were used, causing a new halfway peak to appear.

These findings show that the binding mode involves a significant amount of aggregation, but other mechanisms such as chelation cannot be excluded a priori. It is likely that an interplay of different binding modalities is responsible for the final affinities. Our results would be in accordance with the properties of other β-propeller lectin inhibitors possessing PEG spacers, for which a mixed chelating-aggregating mechanism has been already speculated.^39,48,49^

We tried using atomic force microscopy (AFM) to analyze the arrangement of lectins in cross-linked networks or chains because this technique was applied to the study of size and shapes of supramolecular assemblies formed between LecA and multivalent ligands.^50^ Unfortunately, the heterogeneity of the aggregates was detrimental to AFM characterization, as well as for X-ray crystallography. Our attempts to crystalize the lectin-inhibitor complexes for their study at the molecular level were unsuccessful because the presence of multivalent molecules favored precipitation over the organization of the protein into an ordered crystal. Soaking was impossible too because the thigh packing of the apo crystals did not allow for the diffusion of the compounds, so that no ligand could be observed in the electron density after diffraction.

### Evaluation of Anti-Adhesive Properties

To evaluate the potential of the synthesized inhibitors as anti-adhesive agents, we evaluated their properties in a cell-based infection assay. Because *A. fumigatus* is arguably the most harmful agent in our study, we focused our attention on the inhibition of conidial (asexual spores) adhesion to human lung epithelial cells. Other groups carried out similar analyses to probe the involvement of FleA in the adherence or germination of fungal conidia on pneumocytes.^26,39,51^ While these studies use the bronchial cell line BEAS-2B exclusively, we employed alveolar type II A549 cells to which spore association was also confirmed and linked to FleA recognition.^52^

**Figure 3:**
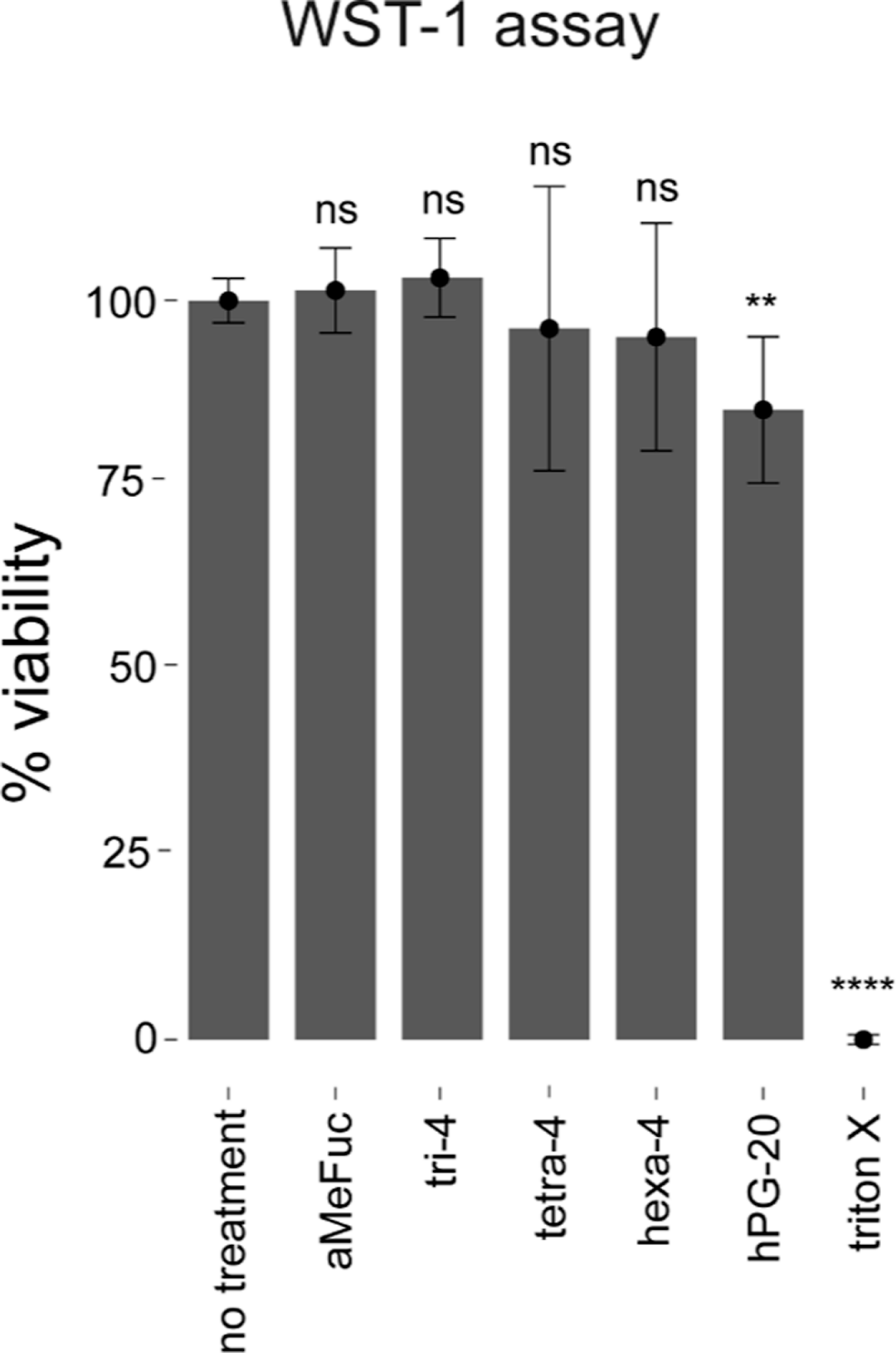
WST-1 viability assay on A549 cells. The concentration of fucosides was 1 mM for **αMeFuc**, 0.1 mM for multivalent compounds. As control, 1% Triton X-100 was used. Values are represented as mean ± CI_95_ from independent replicates, where ns: not significant, ****p < 0.0001, **p < 0.01 (Welch’s unpaired *t*-test).

We checked for the potential toxicity of the inhibitors toward A549 cells using a commercial WST-1 kit to assess cell viability. Apart from the negative control, only **hPG-20** was significantly different from the untreated cells (Figure 3), but with still a high viability of 85% at a 100 μM concentration. Because of their encouraging safety profile, we proceeded to test the anti-adhesive properties of the compounds with the best and worst affinity enhancements per sugar, **tetra-4** and **tri-4**, alongside **αMeFuc** as the monovalent counterpart.

**Figure 4:**
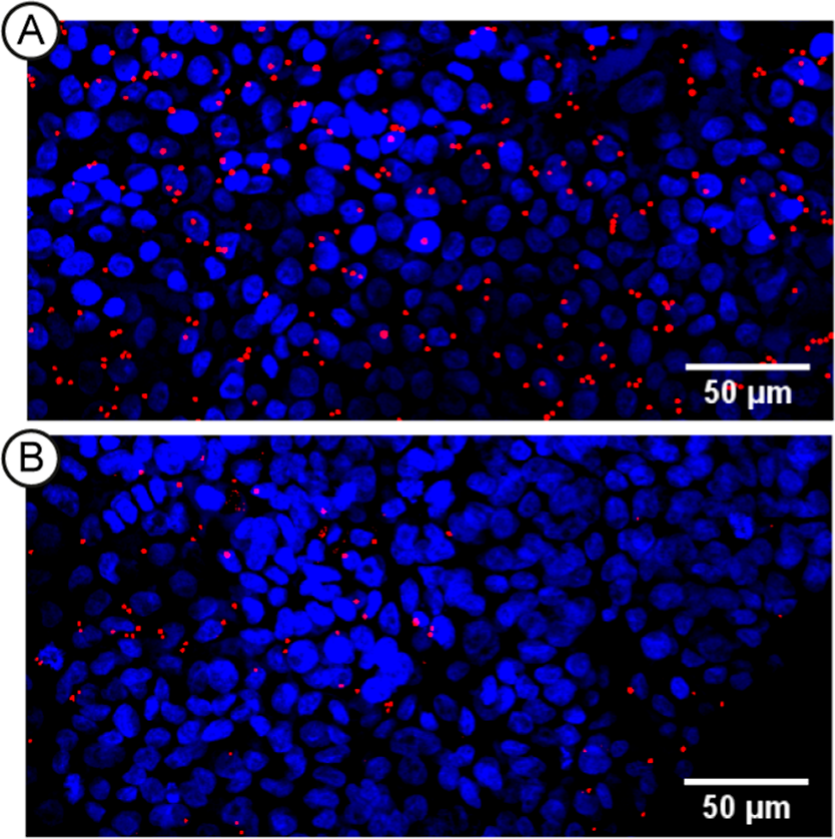
Representative confocal images of spores of fungal strain Af293.1 (red) adhesion on alveolar A549 cells (blue), when cells were untreated (A) or treated with 100 μM **tetra-4** (B). Spores were allowed to adhere for 4 h after infection, followed by washing and staining.

In our adhesion assay, A549 cells were cultured in a well-plate until a confluent layer was obtained and then challenged with *A. fumigatus* conidia in the presence or absence of FleA inhibitors. After incubation and washing to remove unbound spores from the cell layer, imaging was performed by confocal microscopy (Figure 4). The detection of fungi was facilitated by the availability of Af293.1, a strain originally derived from a clinical isolate and genetically modified to constitutively express red fluorescent protein.^53^ Cell nuclei were stained with Hoechst fluorescent dye for visualization.^54^ Cell and spore counts were calculated by ImageJ software and used to express adhesion efficiencies.

**Figure 5:**
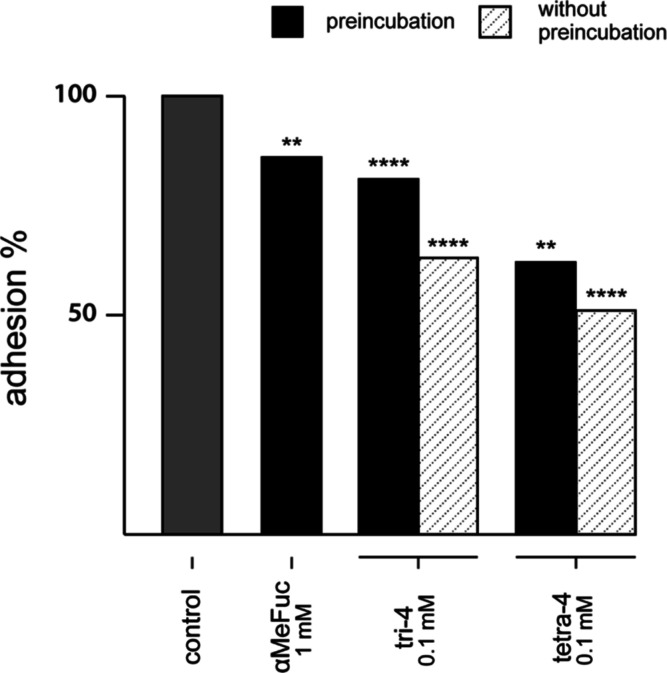
Adhesion of Af293.1 to A459. The black bars refer to the conditions where compounds and spores were pre-incubated 1 h prior infection. The white-striped bars refer to the conditions where compounds were added 1 h after infection. *Significant difference to the control (chi-square test, followed by Bonferroni’s multiple comparison test).

While previous works looked exclusively at differences in adhesion after pre-incubating the spores with fucosides to mimic a prophylactic administration, we additionally investigated the effect of FleA inhibitors post-infection. In the latter setting, the multivalent compounds showed anti-adhesive activity that was even slightly stronger than with preincubation (striped bar in Figure 5). The strongest inhibition was observed with 0.1 mM **tetra-4**, which reduced adhesion by 38% with respect to the untreated control when pre-incubated with spores, and even 49% when added post-infection. At that same dilution, the monovalent **αMeFuc** was ineffective (Supporting Information, Figure S3) and needed 10 times higher concentration (1 mM) to elicit any measurable anti-adhesive properties. An intermediate effect between these two conditions (19–37% reduction of adhesion) was obtained with **tri-4**, as one could forecast from their relative potency. Because of solubility issues, we could not test the unglycosylated core to exclude unspecific effects. However, also **hPG-20** could consistently reduce adhesion in a separate experiment (Supporting Information, Figure S4), suggesting that the anti-adhesion properties observed are not due to unspecific effects of the aromatic scaffold, but rather the multivalent presentation of several fucose moieties. A full blockage of adhesion was not observed under the tested conditions. While it is possible that the most effective concentration was not reached, a more likely scenario may be that other cellular components that mediate adherence are still operative, both on the fungal (RodA, CspA, and AfCalAp) and the host (H-ficolin, E-cadherin, Dectin-1) side.^55^

Given the ability of the multivalent fucosides to aggregate FleA, it is logical to question whether the anti-adhesion properties of such compounds derive from their ability to aggregate spores as well. Although conidial aggregation was reported before with similar inhibitors,^39^ we did not observe significant differences in the number or dimension of fungal spore aggregates in treated versus untreated samples in the absence of cells (Figures S5 and S6, Supporting Information).

## Conclusions

We have here described the preparation of a series of broad-spectrum inhibitors for medicinally relevant six-bladed β-propeller fucose-specific lectins (FleA, SapL1, and BambL). Multivalency plays a major role in adhesive events at early stages of infection, allowing simultaneous low affinity interactions between carbohydrate ligands and their receptors. To block the adhesion of the pathogen to host tissues, we synthesized a set of inhibitors for the targeted lectins that can display 3, 4, 6, or 20 fucoside units. Their dimensions can be easily tuned by the length of the spacer that connects the core of the molecule to the peripheric sugar ligand. Such compounds have the advantage that they can be obtained from cheap starting materials: polyethylene glycols and l-fucose. Although their pharmacokinetic properties still need to be characterized, we run a WST-1 cytotoxicity assay that hints at a good safety profile on alveolar cells. We hope that the characteristics of the scaffold and the multivalency that it provides will ensure not only high affinity but also specificity toward β-propeller lectins of human pathogens, allowing use as biological probes and minimizing unwanted side-effects toward medicinal use.

Potent inhibitors are needed to better elucidate the involvement of β-propeller lectins in pathogenicity and/or immune response. What is more, they are intended for use as anti-adhesive agents in the attempt to block the infection before tissue invasion and worsening of symptoms. Because anti-adhesive therapy does not generate selective pressure on the pathogen, it is particularly compelling in the context of antimicrobial resistance. Our multivalent fucosides fulfill the need for strong inhibition, binding their protein targets with avidities in the nanomolar range, as detected by diverse biophysical methods (FP, ITC, and BLI). They remarkably increase the affinity for fucose, the natural ligand, improving the *K*_d_ hundreds of times per single sugar unit that is incorporated into the final structure. For example, the *K*_d_ of binding toward FleA passed from the low affinity of about 100 μM for the monosaccharide to the high affinity of ca. 0.15 μM for the multivalent **tetra-4**. Especially in the inhibition of the fungal lectins FleA and SapL1, the best results were obtained with the compounds presenting the longest spacer length (PEG_4_), indicating that a long linker is helpful to better accommodate the ligand into the binding pocket in a chelating or cross-linking mechanism. We studied the formation of protein aggregates by AUC; however, the mechanism of binding was not elucidated in completion. It is likely that a mixed behavior is taking place, with a major component of protein aggregation induced by multivalency. Further experimental investigations are needed to fully elucidate the binding modality, from X-ray crystallography to molecular modeling, although these techniques are still challenging for systems of relatively high valency as ours. Despite the fact that a few examples of crystallization of multivalent complexes are available in the literature,^30,56,57^ our attempts were not successful yet.

Next, we were interested to know the effect of lectin inhibition in a biological sense. As a proof of concept for the utilization of the synthesized fucosides, we focused on FleA inhibition directly on the surface of *A. fumigatus* conidia. We evaluated the ability of the compounds to inhibit the association of fungal spores to A549 cells and found that, within the condition that we tested, **tetra-4** was the most effective in reducing adhesion. Anti-adhesive therapy is believed to be beneficial as a prophylactic treatment in preventing microbial infections, in the same way that human milk oligosaccharides are known to lower the risk of bacterial or viral infections in infants.^58^ Following this line, our anti-adhesive assay looked at the adhesion efficacies of conidia where FleA receptors were pre-occupied by the precedent addition of fucosides. Additionally, we looked at the ability of compounds to reverse adhesion after the epithelial layer was already challenged with fungal spores. Surprisingly, the later administration did not worsen the anti-adhesive properties of the multivalent fucosides, indicating that their avidity can drive the protein off the cellular surface. This effect might have to do with the binding kinetics as multivalent binding can increase residence times.^59^ Alternatively, there could be other processes modulating the ability of spores to aggregate that, in turn, affect the chances of association to the host surface.^60^ Before a clear hypothesis can be speculated, more work should be concentrated on expanding the scope to different *A. fumigatus* strains and experimental conditions, especially taking into account the heterogeneity of the system. In fact, it has already been shown that even genetically identical conidia show wide phenotypic variations that can impact pathogenicity depending on the environmental conditions.^61,62^ Nevertheless, this study provides a stimulus for a change of paradigm and gives anti-adhesive agents a potential for treatment not limited to prophylaxis.

We believe that the best chance to contrast microbial infections effectively is via a combination therapy, where multiple virulence factors should be targeted. In this study, FleA inhibition was not enough to arrest the adherence of spores to alveolar cells entirely. This is not surprising because several fungal cellular components take part in recognition events with the respiratory epithelium.^63^ While it has been observed that a single inhibitor blocks pathogen adhesion even in the case of multiple adhesion mechanisms,^64^ this does not seem to be the case here. It may be possible to achieve a synergistic effect through the inhibition of multiple of these targets. We hope our work will expand the arsenal of approaches to the defense against aspergillosis, but not limited to it. The same anti-adhesive agents could be utilized to inhibit fucose-binding lectins to better understand, prevent, and treat other fungal or bacterial lung infections.

## Materials and Methods

### General Information

Chemicals were obtained from commercial sources and were used without further purification unless noted otherwise. Solvents were purchased from Biosolve (Valkenswaard, The Netherlands). Moisture-sensitive reactions were performed under a nitrogen or argon atmosphere. Solvents were dried over activated molecular sieves (4 or 3 Å). Amberlite IR120 H^+^ form was washed with MeOH before using it for neutralization. Chromatographic purifications were performed using 230–400 mesh silica. TLC was performed on Merck precoated Silica 60 F_254_ glass plates. Spots were visualized by UV light and 10% H_2_SO_4_ in MeOH, a KMnO_4_, or ninhydrine stain (prior 10% PPh_3_ in DCM for azides). Microwave reactions were carried out in a Biotage microwave initiator (Uppsala, Sweden). The microwave power was limited by temperature control once the desired temperature was reached. Sealed vessels of 2–5 and 10–20 mL were used. ^1^H and ^13^C NMR were performed on an Agilent 400 MR or a Bruker 600 UtraShield spectrometer. Chemical shifts (δ) are reported in ppm relative to residual solvent signals, and peak assignments were established based on additional ^1^H–^1^H COSY and ^1^H–^13^C HSQC experiments. High-resolution mass spectrometry (HRMS) analysis was performed using an ESI-QTOF II spectrometer (Bruker, Billerica, USA) and Applied Biosystems 4700 MALDI TOF/TOF instrument. Infrared (IR) spectroscopy was performed using universal attenuated total reflectance (UATR) accessory of PerkinElmer Spectrum Two FT-IR. Purification of final products using reparative HPLC was performed with a C_18_ column (Dr. Maisch GmbH) at a flow rate 12.5 mL/min. Runs were performed using a gradient of 100% water to 100% acetonitrile over 125 min. Pure fractions were collected and freeze-dried before characterization. Purity of the final compounds was checked on analytical HPLC (Shimadzu), eluting the compounds on a C_18_ column (Dr. Maisch GmbH) with a gradient of 2% acetonitrile in water to 100% acetonitrile in a run of 30 min at a flow of 0.7 mL/min. The signal was obtained by an UV detector model SPD-20A at wavelength of 254 nm. Integration of the peaks for purity determination was performed in LabSolutions Software (Shimadzu).

### General Procedure for the Synthesis of Functionalized PEGs **4**, **5**, and **6**

The spacers were prepared following procedures described in literature.^32,33,65,66^ To a solution of di- (*n* = 1), tri- (*n* = 2), or tetra- (*n* = 3) ethylene glycol (45 mol, 3 eq.) in 20 mL of dry tetrahydrofuran, metallic sodium (1.5 mmol, 0.1 eq.) was added. At complete dissolution of sodium, *tert*-butyl acrylate (15 mmol, 1 eq.) was added and the solution was stirred for 20 h. After neutralization with 2 mL of HCl 1 M, the residue was dissolved in 100 mL of brine and extracted thrice with 100 mL of EtOAc. The combined organic layers were dried with NaSO_4_. The solvent was evaporated under reduced pressure, and the crude was directly used in the next step. The residue was dissolved in 12 mL of dry DCM and cooled to 0 °C with ice bath. NEt_3_ was added (40 mmol, 3 equiv) to the stirring solution, followed by Ms-Cl (33.5 mmol, 2.5 equiv). After stirring for 16 h, NEt_3_·HCl salt was precipitated and the mixture filtered under applied pressure on Celite. The filtrate was additionally washed with water and NaCl sat. sol. in a separatory funnel. DCM was removed by rotary evaporation. NaN_3_ was added (65 mmol, 5 equiv) to the crude material and both dissolved in 20 mL of DMF. The reaction mixture was stirred overnight at 60 °C, after which DMF was evaporated almost completely. The remaining residue was diluted in EtOAc and washed with water and Brine. Solvent was removed in vacuo and the crude was purified by silica gel column chromatography (PE/EtOAc 9:1 for the spacer with *n* = 2, 8:2 for *n* = 3,4). Products were obtained as pale-yellow oils (yields over three steps: 62% *n* = 1, 63% *n* = 2, 72% *n* = 3). Presence of the azide was confirmed by IR spectroscopy. ^1^H and ^13^C NMR analysis (600 MHz, CDCl_3_) corresponds to what previously reported.^32,33,65,66^ The *tert*-butyl esters obtained in the previous two steps (1 equiv) were dissolved in 30 mL of DCM and 10 equiv of TFA was added to the solution, which was stirred at r.t. for 1.5 h. Then, the solvent was evaporated and the product was obtained as a yellowish oil after flash chromatography (DCM/MeOH 9:1). Yields obtained were as follows: 90% for spacer **4** (*n* = 1), 97% for **5** (*n* = 2), and 79% for **6** (*n* = 3). ^1^H and ^13^C NMR analysis (600 MHz, CDCl_3_) corresponds to what previously reported.^32,33,65,66^

### General Procedure for the Synthesis of Elongated Ligands **10**, **11**, and **12**

Peptide coupling reaction was performed as follows: 1 eq. of amino fucoside **3**, 2.5 equiv of either **4**, **5**, or **6**, and 3 equiv of HATU were dissolved in dry DCM under an inert atmosphere. DIPEA (3.5 equiv) was immediately added to the solution. The mixture was stirred overnight at room temperature and then washed in a separatory funnel with HCl 1 M, NaHCO_3_, and NaCl sat. solutions. The organic phase was dried over sodium sulfate, filtered, and the filtrate was dried in vacuo. The residual crude was purified by flash chromatography. EtOAc 100% → EtOAc/MeOH 95:5 was used as the eluent in the case of azide **7** (*n* = 2) and **9** (*n* = 4), while 1–5% MeOH in DCM was chosen for the purification of **8** (*n* = 3). Yields obtained were as follows: 37% for **7**, 53% **8**, and 34% **9**. Azide reduction was performed as follows: **7**, **8**, or **9** (1 equiv) and SnCl_2_ (2.5 equiv) were dissolved in MeOH, and 1 equiv of HCl from a 2 M aqueous solution was added to the stirring mixture. After 4 h and 30 min, the solvent was evaporated and the resulting crude was purified by silica gel chromatography (DCM/MeOH 9:1, 0.1% NEt_3_), giving yellowish fluffy solids. The yield for the fractions containing NEt_3_ was corrected accordingly. A full conversion was calculated for each of the amino compounds with different PEG lengths.

### General Procedure for the Synthesis of the Cores **14**, **15**, and **16**

Compound **13** (2 equiv per core valency) and the appropriate bromomethyl benzene (1 equiv) were dissolved under an Ar atmosphere in dry DMF. NaH 60% in mineral oil (2.5 equiv per core valency) was added at 0 °C to the stirring solution, which turned yellow to orange upon addition. Reaction was stirred 17 h at room temperature. Reaction work-up consisted in liq/liq extraction in EtOAc and washing the organic phase with NaCl sat. solution and water. Organic solvent was removed by rotary evaporation and the remaining crude was purified by silica gel chromatography. The ester hydrolysis was performed by stirring the compound solubilized in a solution of 2 M NaOHaq/THF/dioxane 1:1:2 (final concentration 0.1 M) for 16 h. At full conversion, Amberlite IR-120 H^+^ resin was added until neutral pH and then filtered. The filtrate was evaporated in vacuo.

### General Procedure for the Synthesis of Multivalent Compounds

#### Trivalent Series

Acid **14** (1 equiv), HATU (4.5 equiv), and **10**, **11**, or **12** (12 equiv) were dissolved in dry DMF under an inert atmosphere. Immediately after the solvent, also DIPEA (16 equiv) was added to the mixture at r.t. The orange solution was stirred overnight and then diluted in EtOAc and transferred into a separatory funnel. The organic solution was washed with water, HCl 1 M, NaHCO_3_, and NaCl sat. solutions. The organic phase was dried over sodium sulfate, filtered, and dried by rotary evaporation. The residual crude was purified by flash chromatography. MeOH 5–10% in DCM was used as the eluent in the case of **tri-3** and **tri-4**, while for the purification of **tri-2** was chosen a gradient of 1–8% MeOH in DCM. The obtained acetylated compounds were deprotected in a 0.1 M solution of MeONa in MeOH. Solution was stirred overnight at r.t. and neutralized with Amberlite IR-120 H^+^ when the reaction was completed. The solution was filtrated to remove the resin and then dried by rotary evaporation and at the high vacuum pump. A pure white solid was obtained in quantitative yield for the second step. Compounds were additionally purified by HPLC and freeze-dried. Final compounds were obtained as white powders after final purification (**tri-2**: 59% before HPLC, 33% after; **tri-3**: 28% before HPLC, 14% after; and **tri-4**: 65% before HPLC, 23% after).

#### Tetravalent Series

Acid **15** (1 equiv), HATU (6 equiv), and **10**, **11**, or **12** (16 equiv) were dissolved in dry DMF under an inert atmosphere. Immediately after the solvent, also DIPEA (16 equiv) was added to the mixture at r.t. The orange solution was stirred overnight, and then, work-up was performed as described for the trivalent series. The crude was purified by flash chromatography in eluent DCM/MeOH 95:5 → 90:10. The obtained compounds were deacetylated as described above for the trivalent series. Compounds were additionally purified by HPLC and freeze-dried. Final compounds were obtained as white powders after final purification (**tetra-2**: 24% before HPLC, 13% after; **tetra-3**: 24% before HPLC, 20% after; **tetra-4**: 35% before HPLC, 17% after).

#### Hexavalent Series

Acid **16** (1 equiv), HATU (9 equiv), and **10**, **11**, or **12** (24 equiv) were dissolved in dry DMF under an inert atmosphere. Immediately after the solvent, also DIPEA (30 equiv) was added to the mixture at r.t. The orange solution was stirred overnight, and then, work-up was performed as described for the trivalent series. The crude was purified by flash chromatography in eluent DCM/MeOH 95:5 → 85:15 for hexa-2, while DCM/MeOH 95:5 → 90:10 was the gradient chosen for the rest. The obtained compounds were deacetylated following the procedure described before. Compounds were additionally purified by HPLC and freeze-dried. Final compounds were obtained as white powders after final purification (**hexa-2**: 29% before HPLC, 13% after; **hexa-3**: 31% before HPLC, 10% after; and **hexa-4**: 38% before HPLC, 11% after).

### Synthesis of Polyglycerol-Fucose Conjugate (**hPG-20**)

The polymer hPG-OH has been synthesized according to the reported procedure.^38,67,68^ To a solution of hPG-OH (186 mg, 0.26 mmol OH groups) in 5 mL of anhydrous DMF, NaH (13.9 mg, 0.57 mmol, 2.2 equiv, 60% dispersion in mineral oil) was added. After stirring for 3 h at rt, potassium iodide (8.7 mg, 0.052 mmol, 0.2 equiv) was dissolved in a minimum amount of DMF and added to the reaction mixture. After cooling the mixture to 0 °C, propargyl bromide (40 μL, 0.44 mmol, 1.7 equiv) was added and stirred overnight. The reaction mixture was extracted with EtOAc with ethyl acetate (3 × 60 mL), the combined organic layers were concentrated in vacuo and the crude product was purified by dialysis in chloroform (48 h) to obtain a light brown viscous oil. The IR spectra showed a visible C≡CH stretching peak at 2112 cm^–1^. Carbon NMR is used to calculate the relative abundance of the different protons that compose the polymer. The calculated percentage of propargyl was calculated to be 16% of 125 OH groups in hPG-OH = 20 end groups. The molecular weight of hPG-propargyl was calculated to be 10494 g/mol and the yield 72%. The hPG-propargyl polymer was dissolved in water followed by the addition of the fucose ligand (1.3 equiv per propargyl). Separately, 0.1 equiv of copper sulfate pentahydrate was dissolved in water and added to the reaction mixture. 0.3 equiv of sodium ascorbate was also dissolved in water separately and added to the reaction mixture. The reaction was carried out at 100 °C in the microwave for 60 min. CupriSorb (Seachem) resin was added to the reaction mixture and stirred, followed by filtration of the resin. The solvent was evaporated and the crude reaction mixture was purified by dialysis using a cellulose-based dialysis cassette (MWCO: 2 K) against deionized water for 3–4 days and freeze-dried to obtain the final compound **hPG-20** in 91% yield. The final compound was characterized using NMR and IR. The molecular weight of **hPG-20** was calculated as follows: 10494 + 20 × 232.2 = 15138 g/mol.

## Supporting Information Available

Methods for binding and cell-based assays, HPLC traces, HRMS results, and NMR characterization of final compounds (PDF)
