# Supplementary material for: Multivalent Fucosides Targeting β-Propeller Lectins from Lung Pathogens with Promising Anti-Adhesive Properties
Source: ACS Chem Biol. 2022 Nov 22;17(12):3515–26. doi: 10.1021/acschembio.2c00708 (PMC9764287; doi:10.1021/acschembio.2c00708)
Supplement: Supplementary file 1 — cb2c00708_si_001.pdf [file cb2c00708_si_001.pdf]

Supporting Information

Multivalent fucosides targeting β-propeller lectins from lung pathogens with promising anti-adhesive properties

Margherita Duca,[†,‡,§] Diksha Haksar,[†] Jacq van Neer,[‡] Dominique M.E. Thies-Weesie,[||] Dania Martínez-Alarcón,[§] Hans de Cock,[‡,*] Annabelle Varrot,[§,*] and Roland J. Pieters.[†,*]

[†]Department of Chemical Biology & Drug Discovery, Utrecht Institute for Pharmaceutical Sciences, Utrecht University, Utrecht NL-3508 TB, The Netherlands.

[‡]Department of Biology, Utrecht University, Padualaan 8, 3584 CS Utrecht, The Netherlands.

[||]Debye Institute for Nanomaterials Science, Utrecht University, Padualaan 8, 3584 CS Utrecht, The Netherlands.

[§]Univ. Grenoble Alpes, CNRS, CERMAV, 38000 Grenoble, France.

*Corresponding authors

Email: r.j.pieters@uu.nl, annabelle.varrot@cermav.cnrs.fr, h.decock@uu.nl

Summary:

1. Binding assays S2

1.1 Fluorescence polarization competitive assay S2

1.2 Isothermal titration calorimetry S3

1.3 Microcalorimetry titrations S4

1.4 Biolayer interferometry S8

2. Analytical Ultracentrifugation S9

3. WST-1 viability assay S10

4. Adhesion assay S11

5. Spore aggregation S13

6.Synthesis S14

6.1 Ligand S14

6.2 Spacer-elongated ligands 10, 11, 12 .... S15

6.3 Cores 14, 15, 16 .... S16

6.4 Multivalent fucosides S17

6.5 Polyglycerol-fucose conjugate (hPG-20) S20

7. Characterization of final compounds S21

8. HRMS traces S32

9. References S37

1. Binding assays

1.1 Fluorescence polarization competitive assay

The fluorescent ligand **Fuc-FITC** (Figure S1) was prepared from compound **2a** following a protocol reported in literature.[1]
Typically, a stock solution of protein (final concentration 100 nM) and **Fuc-FITC** (final concentration 6 nM) were added to 3-fold serial dilutions of testing compounds in duplicates on a black 96 well microtiter plate (COSTAR™ 96). The starting concentration for multivalent compound were 47 µM for FleA, 6 µM for SapL1, 1 µM for BambL. The starting concentration for the monovalent compound was 3 mM and 5-fold dilutions were used in this case. All components were dissolved in the same buffer (20 mM Tris, 100 mM NaCl, pH 8.0). After addition of the reagents, the microtiter plates were shaked for 1 h at room temperature in the dark. The fluorescence was measured using a CLARIOstarPlus plate reader (BMG Labtech) with excitation filters at 480 nm and emission filters at 530 nm. The same gain was maintained for all measurement relative to the same protein. A fluorescence intensity spectral scan of the multivalent compounds alone was also performed to confirm no interference with the analysis. The data were analyzed with CLARIOstar Mars Software using a 4-parameter fit. The point of inflection was used to determine the IC_{50} value of each experiment. The standard deviation was calculated over the two independent IC_{50} values obtained in Microsoft Office Excel suite.

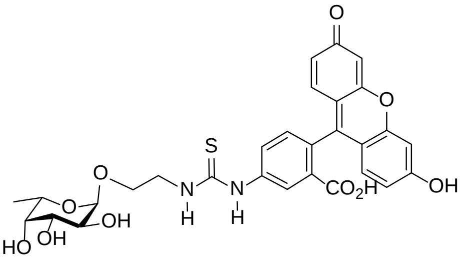

Figure S1. The structure of the fluorescent probe **Fuc-FITC.**

1.2 Isothermal titration calorimetry

Isothermal titration calorimetry was performed on a Microcal VP-ITC (Malvern Panalytical) and the data was analyzed using the Microcal PEAQ-ITC Analysis Software (one set of sites model). Protein targets were placed in the sample cell at concentrations ranging between 50 and 8.5 µM at 25 °C. The titration was performed with 30 injections of a solution of monovalent compound (1 or 3 mM) or multivalent compound (range 200-15 µM) in the same buffer as the protein (20 mM Tris, 100 mM NaCl, pH 8.0). At least two independent titrations were run and standard deviations were calculated over the average value of the obtained parameters.
The complete thermodynamic analysis of the interactions is displayed in the tables above (Table S1-S3).

Table S1. Microcalorimetry data relative to FleA binding.

	v	K_d (nM)	N	ΔH (kJ/mol)	-TΔS (kJ/mol)
**2a**	1	**97600** ± 300	**4.1** ± 0.02	**-39** ± 4	**16** ± 4
**tri-4**	3	**240** ± 24	**1.4** ± 0.2	**-112** ± 1	**74** ± 1
**tetra-4**	4	**149** ± 17	**1.2** ± 0.03	**-185** ± 3	**145** ± 3
**hexa-4**	6	**115** ± 4	**0.8** ± 0.02	**-246** ± 6	**206** ± 6
**hPG-20**	20	**60** ± 4	**0.4** ± 0.03	**-335** ± 1	**293** ± 1

Table S2. Microcalorimetry data relative to SapL1 binding.

	v	K_d (nM)	N	ΔH (kJ/mol)	-TΔS (kJ/mol)
**αMeFuc**	1	**68100** ± 4400	**3.52** ± 0.13	**-63** ± 11	**27**± 2
**tri-4**	3	**60** ± 3	**1.27** ± 0.12	**-105** ± 5	**64**± 5
**tetra-4**	4	**60** ± 2	**0.95** ± 0.03	**-160** ± 8	**119**± 8
**hexa-4**	6	**25** ± 3	**0.72** ± 0.02	**-199**± 13	**155** ± 12
**hPG-20**	20	**19** ± 2	**0.32** ± 0.04	**-335** ± 1	**291** ± 1

Table S3. Microcalorimetry data relative to BambL binding.

	v	K_d (nM)	N	ΔH (kJ/mol)	-TΔS (kJ/mol)
**2a**	1	**2135** ± 80	**1.81** ± 0.01	**-50** ± 1	**17** ± 1
**tri-4**	3	**3.7** ± 0.7	**0.97** ± 0.1	**-105** ± 11	**57** ± 10
**tetra-4**	4	**3.0** ± 0.9	**0.49** ± 0.04	**-201** ± 11	**152** ± 12
**hexa-4**	6	**4.7** ± 0.9	**0.35** ± 0.01	**-296** ± 9	**249** ± 10
**hPG-20**	20	**2.1** ± 0.1	**0.14** ± 0.01	**-335** ± 0.1	**286** ± 1

1.3 Microcalorimetry titrations of FleA, BambL and SapL1 with fucosides.[a]

[a]Only one of the replicates is shown for clarity.

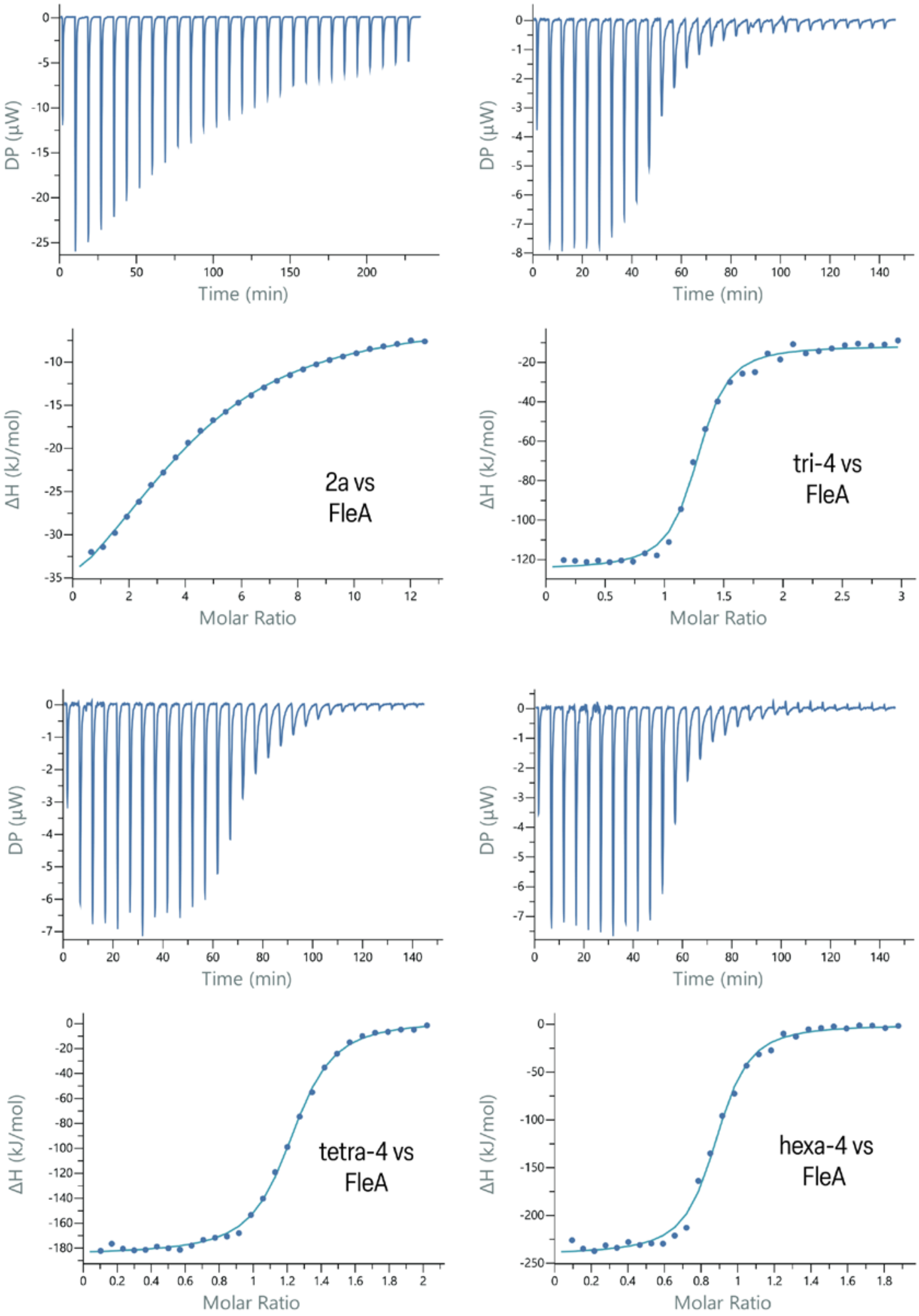

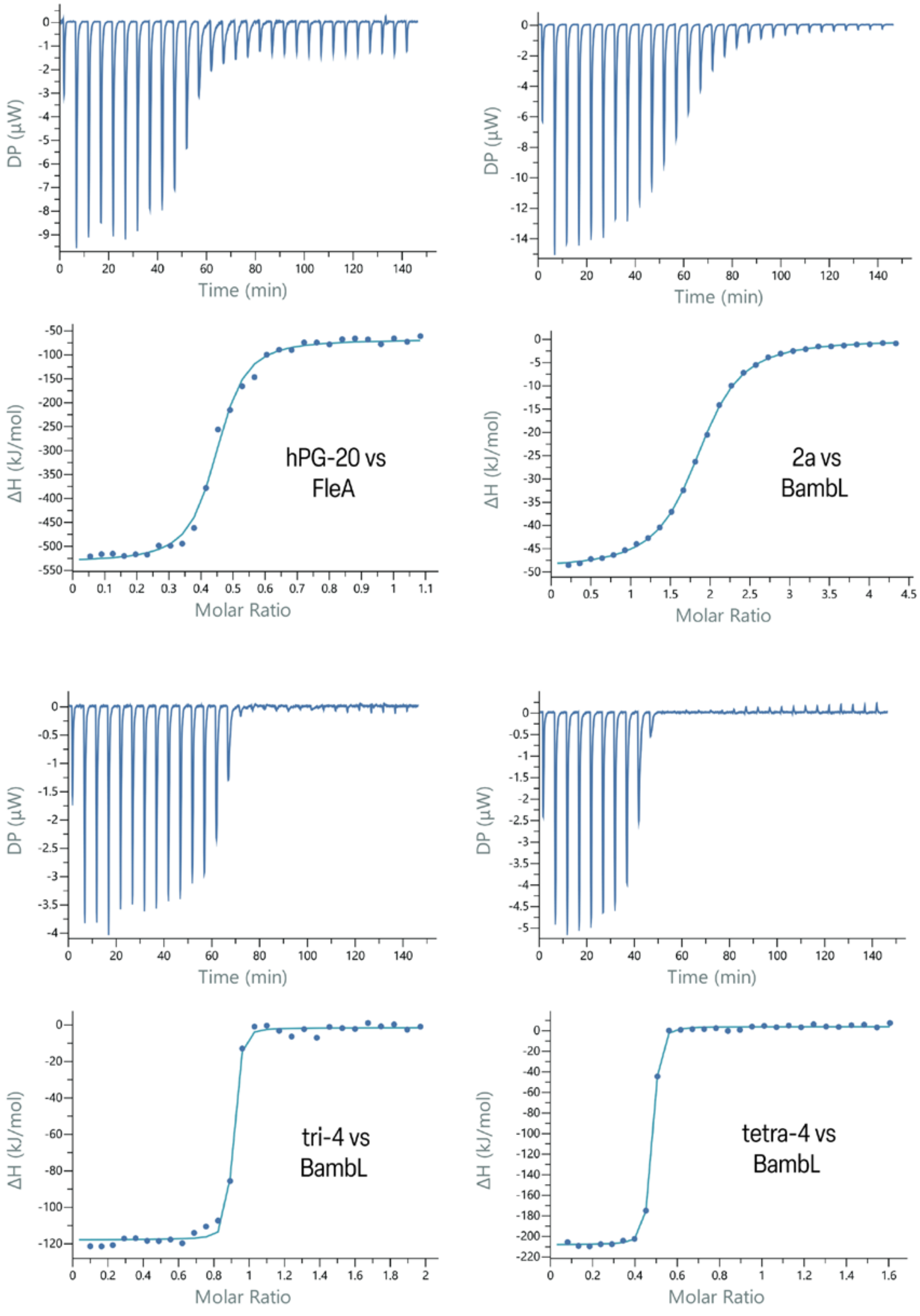
DP (µW)
Time (min)
ΔH (kJ/mol)
Molar Ratio
hPG-20 vs
FleA
2a vs
BambL
tri-4 vs
BambL
tetra-4 vs
BambL

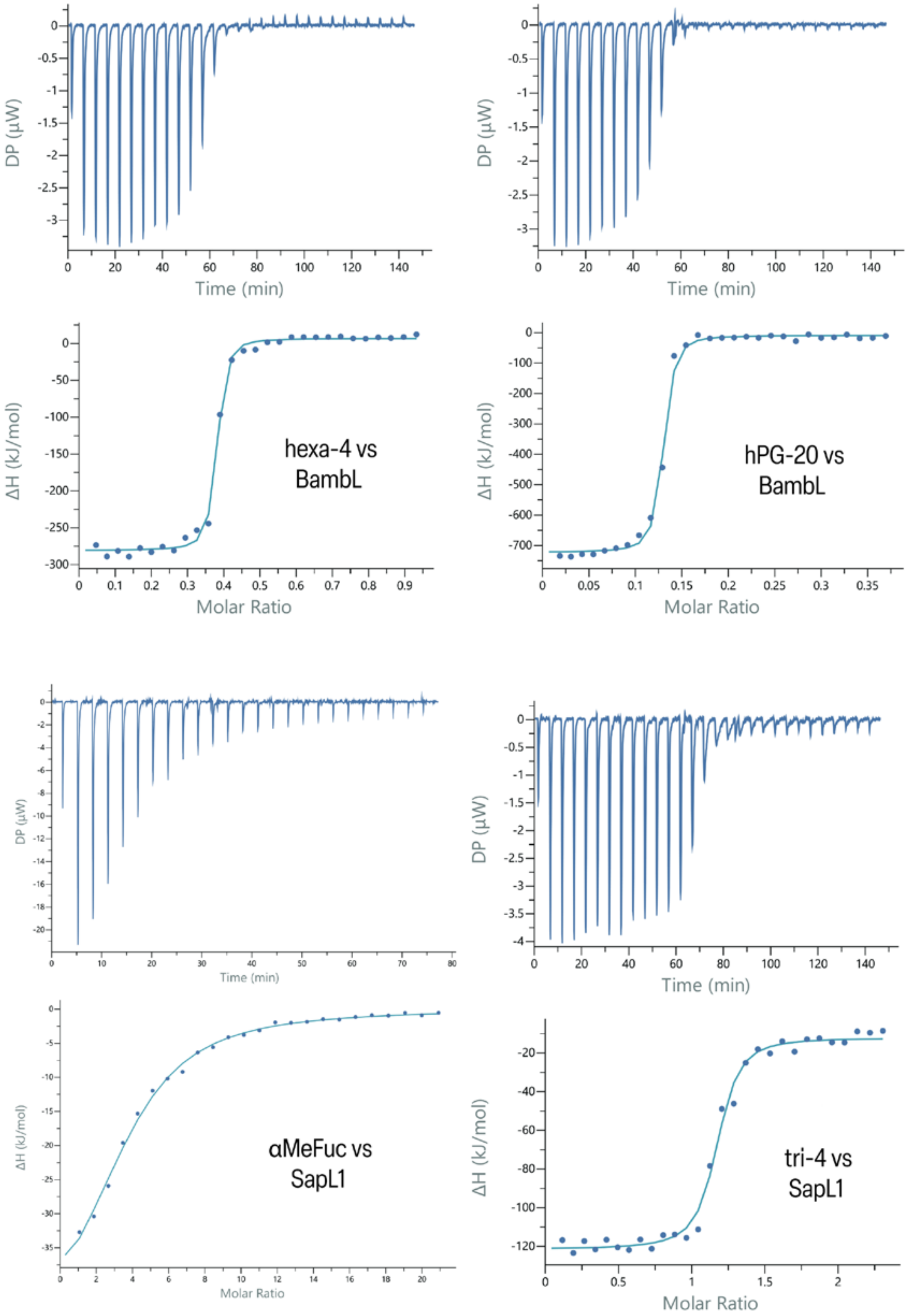

DP (µW)
Time (min)
ΔH (kJ/mol)
Molar Ratio
hexa-4 vs
BambL
DP (µW)
Time (min)
ΔH (kJ/mol)
Molar Ratio
hPG-20 vs
BambL
DP (µW)
Time (min)
ΔH (kJ/mol)
Molar Ratio
αMeFuc vs
SapL1
DP (µW)
Time (min)
ΔH (kJ/mol)
Molar Ratio
tri-4 vs
SapL1

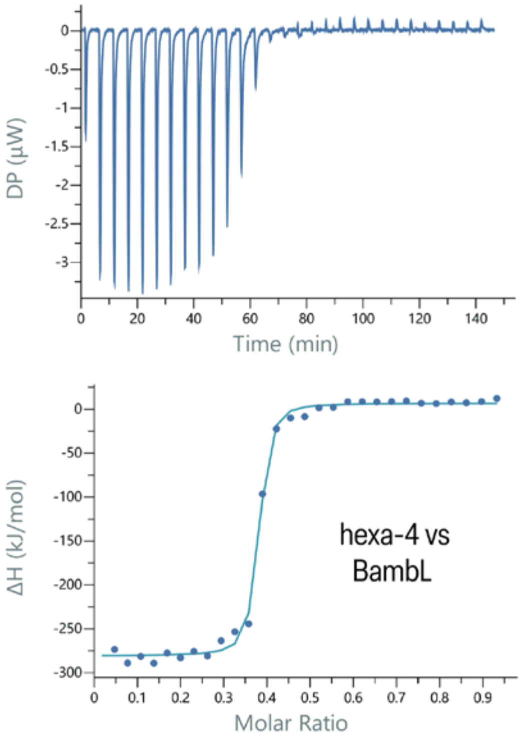
DP (μW)
Time (min)
ΔH (kJ/mol)
Molar Ratio
hexa-4 vs
BambL

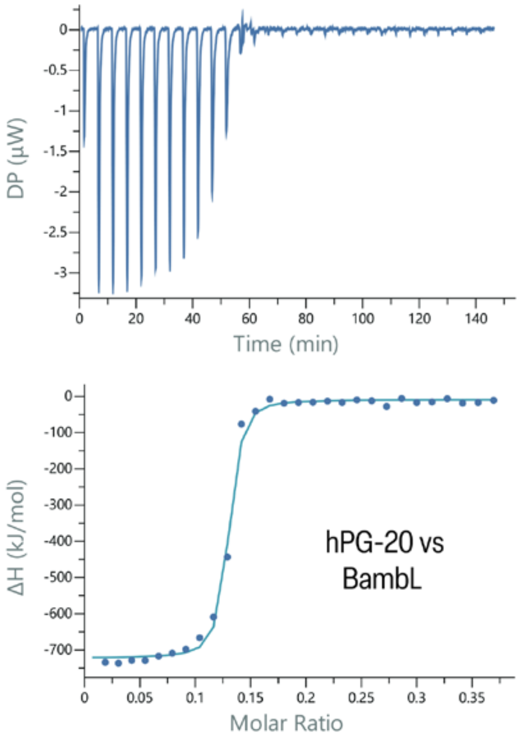
DP (μW)
Time (min)
ΔH (kJ/mol)
Molar Ratio
hPG-20 vs
BambL

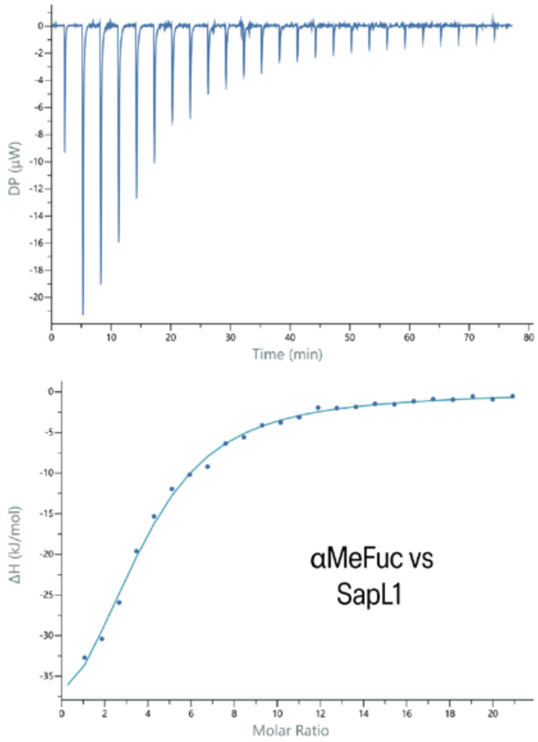
DP (μW)
Time (min)
ΔH (kJ/mol)
Molar Ratio
αMeFuc vs
SapL1

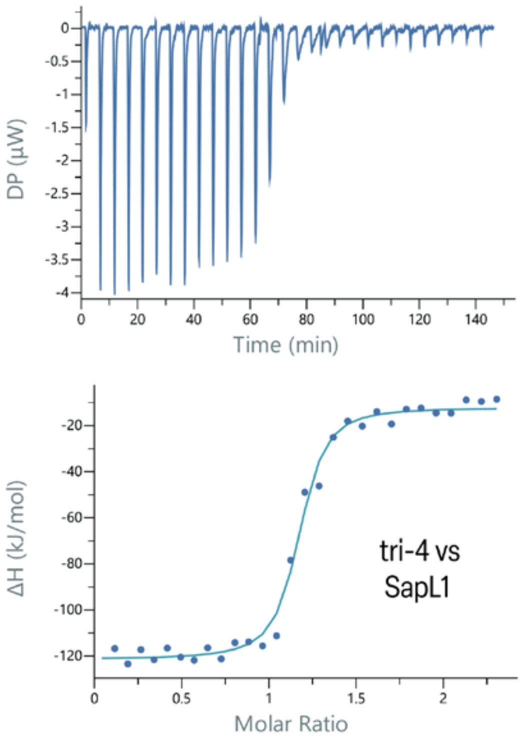
DP (μW)
Time (min)
ΔH (kJ/mol)
Molar Ratio
tri-4 vs
SapL1

1.4 Biolayer interferometry

Prior analysis, proteins (10 mg/ml in PBS solution at pH 7.4) were biotinylated using 12 equivalents of biotin from a 100 mM solution of **biotinamidohexanoyl-6-amino-hexanoic acid-hydroxy-succinimide ester** (Sigma-Aldrich) in DMF. The mixture was incubated for 30 minutes with stirring. Afterwards, the excess of biotin and solvent was removed on a desalting column, eluting the protein with PBS buffer. The binding interaction between the biotinylated proteins and multivalent compounds was analyzed with an Octet Red96 instrument (ForteBio) using a streptavidin sensor. Biotinylated protein at 100nM concentrations were loaded on the sensor. After a washing and equilibration step, the sensor was submerged in solutions of multivalent fucoside at different concentrations (range 1μM-10nM) to obtain the relative association curve. The sensor was dipped in a solution containing only buffer to dissociate the compound, although full dissociation was never observed. The binding event with the monovalent reference could not be detected due to low sensitiveness of the technique. Resulting curves were analyzed by ForteBio Data Analysis 9.0 Software, using a full association and dissociation fit to extrapolate K_d values and relative errors.

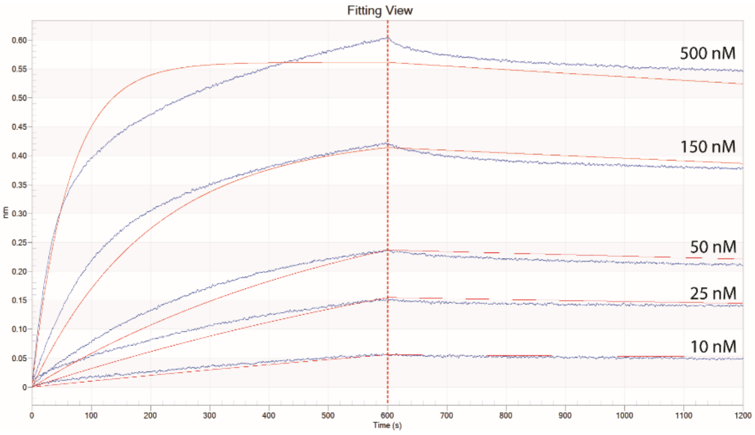

Figure S2. Representative BLI curves of FleA with the fucose-polymer **hpg-20**.

2. Analytical Ultracentrifugation

Samples of FleA and BambL and mixtures of them with different equivalents of compound were prepared just before the measurements. The 12 mm path length 2-sector aluminum centerpiece cells with sapphire windows, filled with about 380 μL sample and 400 μL buffer as a reference, were put in an An60Ti rotor in a Proteomelab XL-I analytical centrifuge (Beckman Coulter) at a temperature of 20°C. First a wavelength scan was performed at 3000 rpm in the middle of the cell to check the height of the absorbance peak at 280nm. This height is proportional to the concentration of material present (that absorbs at 280nm). Then a single velocity scan was performed at 280nm to see if the absorbance is constant over the height of the cell. If absorbance is not constant or not equal to the value of the wavelength scan, there is sedimentation (at this low speed) indicating the presence of very large (probably ill-defined) aggregates. High-speed experiments were not performed on large aggregates. If the absorbance is constant, the machine is stopped, the sample is homogenized and then the AUC is run at 60000 rpm. Changes in solute concentration were detected by 250 absorbance scans measured at 280 nm over a period of 5–6 h.

Analysis and fitting of the data was performed using the software SedFit v.14.3.[2] A continuous c (s) distribution model was fitted to the data, taking every 2nd scan. The resolution was set at 200 over a sedimentation coefficient range of 0.0–15.0 S. Parameters were set for the partial specific volume at 0.7068 mL/g for BambL, the buffer density of 1.00298 g/mL, and the buffer viscosity at 1.0165 mPa s, as calculated using SEDNTERP v3.0.3 for 100mM NaCl/20mM Tris buffer at pH=8. The frictional coefficient, the baseline, and the raw data noise were floated in the fitting. The meniscus
was also floated after initial estimations from the raw data.

3. WST-1 viability assay

A549 cells were seeded in a microtiter plate in a concentration of 10^4/well and grown for one day to almost full confluence at 37°C and 5% CO_2 atmosphere. Compounds (or only buffer for the positive control experiment) were added at specific concentrations (see Table S4) and the plate was incubated for other 24 h, after which the WST-1 reagent was added to each well. Triton X-100 is a common detergent used for cell lysis; as such, it was added 10 minutes prior the WST-1 to have a negative control. Cells were incubated with the cell proliferation reagent for 1h to allow the color to develop. Each condition was repeated in at least triplicates. Absorbance was detected at 450 nm with a BioTek Synergy microplate reader. Viability was measured in relation to the absorbance values according to the manufacturer's protocol (Cayman Chemical). Statistical analysis was performed in Rstudio Software to calculate means values and confidence intervals (CI_{95}). Significance was analyzed using the Welch's unpaired t-test (P-values of ≤0.05 were considered significant). The original absorbance data are reported above in Supporting Table 4. A scan of absorbance between 300 and 700 nm was also read, to confirm no interference of inherent compounds' absorbance with the WST-1 probe.

Table S4. WST-1 assay results: average of absorbance data at 450 nm and related confidence intervals. The significance is expressed in relation to the control (buffer).

Mean	Group	CI_{95}
3.014333	buffer	0.082853
0.2505	tritonX 1%	0.017635
3.1	tri4 0.1mM	0.146878
2.910333	tetra4 0.1 mM	0.532091
2.880333	hexa4 0.1 mM	0.425659
2.606	hpg20 0.1 mM	0.273266
305.275	αMeFuc 1 mM	0.158603

4. Adhesion assay

The adhesion assay was designed similarly to what previously published.[3] 200 μL of A549 cells at a starting concentration of $2x10^5$ were grown on 8-mm glass coverslips (ThermoFisher Scientific) until a confluent layer was formed. Conidia were preincubated with fucosides at different concentrations for 1 hour, then added ($5x10^5$ spores per well, MOI 1) to A549 cells and incubated for additional 4 h. In conditions without pre-incubation, the compounds were directly added to the wells after 1 h of infection. Unbound conidia were removed carefully by washing 4 times with media (DMEM + 10% FBS). Cells were fixed with 4% paraformaldehyde for 10 min at room temperature. To count the total number of cells per field, 1 μg mL^{-1} Hoechst stain (BD Biosciences) was added to each well and incubated for other 10 minutes. Af293.1 conidia express a plasmid containing a fluorescent RFP gene for visualization. Coverslips were mounted with FluorSave™ (Merck Millipore) onto glass slides and dried overnight prior to confocal analysis. Each condition was replicated on different coverslips. Confocal imaging was performed on a Zeiss LSM 700 microscope using an EC Plan-Neofluar 40x/1.30 oilDIC M27 objective. Images were taken using the 405 and 555 nm laser lines. Fluorescence emission of Hoechst was detected using the 400-600 nm spectral band and red fluorescence emission of RFP was detected with the 560-700 nm spectral band. The Z-stacks of about 10 fields per coverslip were randomly chosen and used as technical replicates. Fiji image processing package of ImageJ (www.fiji.sc) was used to calculate the number of nuclei and spores in each image. Statistical analysis was performed comparing the expected number of bound spores on the cellular surface of a field to the experimentally observed values. Normalization of the cell number was not done because each replicate had a significantly similar number of cells and to avoid overtreatment of data. However, cell count in the control experiment was not equal to the rest but about 1/6 lower. Even though this difference, the anti-adhesion effect of the treatment was significant, indicating that an even stronger result could be obtained in presence of more reproducible cell counts. Statistical analysis was performed by a chi-square test, followed by Bonferroni's multiple comparison test. SPSS software and Microsoft Office Excel suite were used for calculations.

Prior to the anti-adhesion assay with multivalent compounds, a separate experiment with only **αMeFuc** at a concentration of 1mM and 0.1 mM was run as a control assay (Figure S3). For this, cells were infected with a MOI of 2. The cell layer was treated with spores that were preincubated with the compound (no post-addition). Statistical analysis was performed on Rstudio Software, simplified to an average ratio of conidia/cells and expressed in percentages in relation to the control. The graph was plotted with bars relative to standard error.

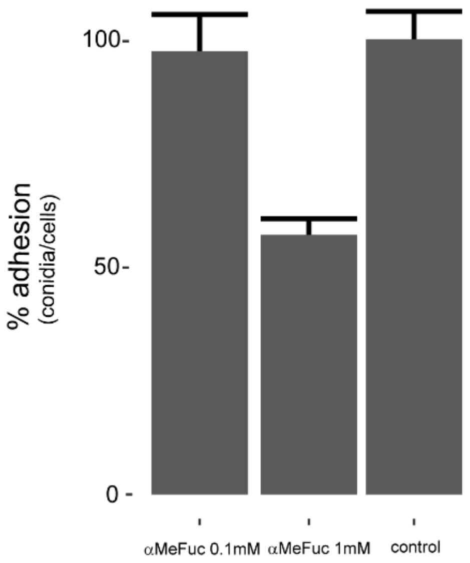

Figure S3. Control experiment for the anti-adhesion assay with **αMeFuc** at different concentrations.

To make sure that the aromatic core in **tetra-4** and **tri-4** has no unspecific effect that influences the anti-adhesive properties of the compound, we additionally tested the fucoside with polymeric scaffold, **hPG-20**, in a separate experiment. The compound was tested at the concentration of 0.1 mM as the other multivalent compounds (Figure S4). For this, the cell layer with spores that were preincubated with the compound (no post-addition) at a MOI 2. Statistical analysis was performed on Rstudio Software, simplified to the average ratio of conidia/cells and expressed in percentages in relation to the control. The graph was plotted with bars relative to standard error.

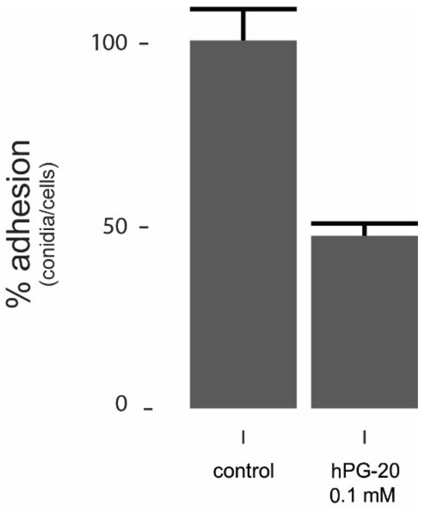

Figure S4. Adhesion of Af293.1 to A459 in presence and absence of **hPG-20**.

5. Spore aggregation

Solutions of spores in presence or absence of multivalent compounds were plated into a Corning® 96-well Clear Flat Bottom UV-Transparent Microplate (in absence of cells). Imaging was performed using an oCelloScope™ system (BioSense Solutions). Uniexplorer 9.0.0.7805 Software (BioSense Solutions) was used to distinguish aggregated spores from single spores. MO Excel was used to calculate the relative ratios, standard deviations and plot graphs at Figure S5 and S6. Figure S5 shows no significant difference between the number of aggregated spores in the control experiment or in the presence of multivalent compounds at the concentration used for the anti-adhesion assay. Due to limited amount of synthetic compound available, an experiment that shows no concentration dependence was run on the compound tri-2 (Figure 6). For this experiment, 4000 spores/well were plated in six replicates for each condition. The size of aggregates was not determined quantitively because not more than 3 spores can be recognized in an aggregate image. However, no striking differences were observed by naked eye.

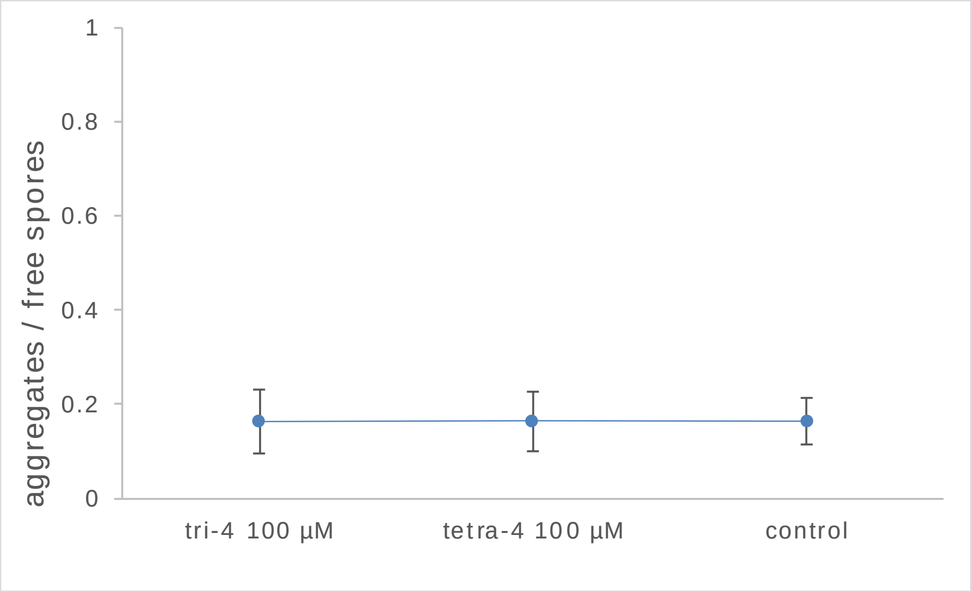

Figure S5. Compound **tri-4** and **tetra-4** do not increase the number of aggregated spores in solution.

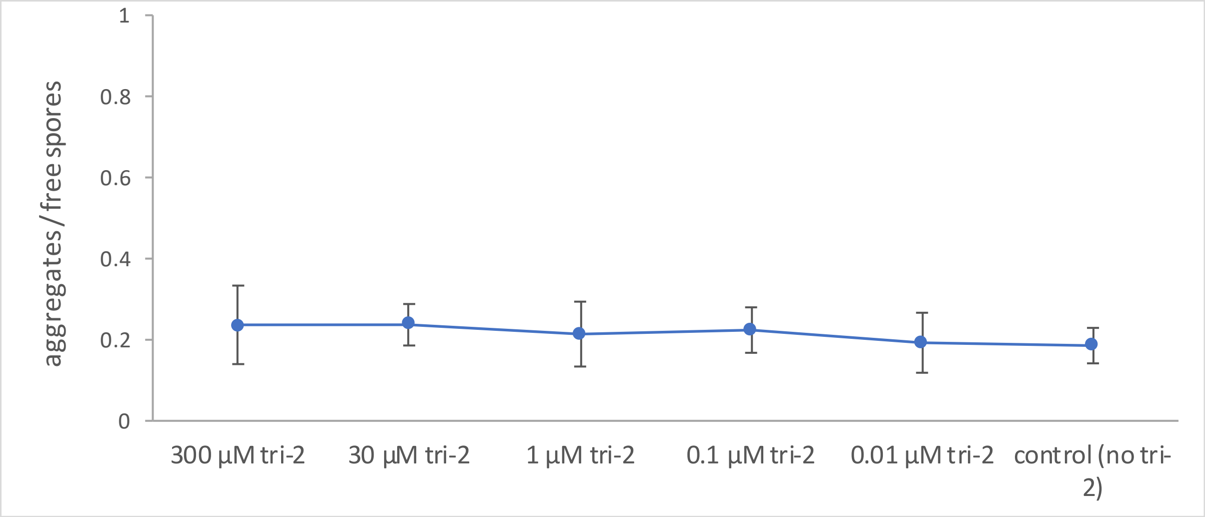

Figure S6. The number of aggregated spores is not dependent on the concentration of compound **tri-2.**

6.Synthesis

6.1 Ligand

2-Chloroethyl 2,3,4-tri-*O*-acetyl-L-fucopyranoside (1)

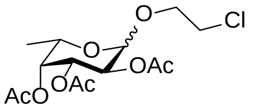

L-fucose (1.065g, 6.5 mmol, 1 eq.) was suspended in 6 mL of **2-Chloroethanol**. **Sulfamic acid** (126 mg, 1.3 mmol, 0.2 eq.) or **Amberlite IR-120 H$^+$** resin (100 mg/mmol of fucose) was added to the mixture. The solution was refluxed upon stirring overnight.
The resin was filtrated off the mixture, then the alcohol was evaporated and the crude material purified by flash silica chromatography (DCM→DCM:MeOH 85:15). After rotatory evaporation of the solvents, product was collected as an α,β mixture. The compound was dissolved in 6 mL of **pyridine** and 3 mL of **Ac_2O** were added to the solution at 0°C. After 16 hours of stirring at r.t., the solution was diluted in EtOAc, transferred in a separatory funnel and washed with HCl 1M, $NaHCO_3$ sat. and Brine solution. The organic phase was dried over sodium sulfate and evaporated *in vacuo*. Peracetylated fucoside **1** was obtained with a yield of 68% (1.565 g) in a α:β ratio of approximately 7:3.
^{1}H and ^{13}C NMR analysis corresponds to what previously reported in literature.[4]

2-Azidoethyl 2,3,4-tri-*O*-acetyl-α-L-fucopyranoside (2)

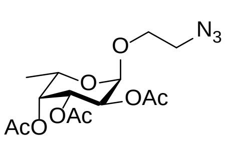

1 (680 mg, 1.9 mmol, 1 eq.), NaN_3 (618 mg, 9.6 mmol, 5 eq.) and TBAI (140mg, 0.4 mmol, 0.2 eq.) were dissolved in 4 mL of DMF. The mixture was stirred at 90°C for 6 hours, until it reached full conversion. Reaction work-up consisted in liquid extraction in AcOEt, washing the organic solution two times with water and once with Brine solution. After drying over $NaSO_4$, the filtrate organic solution was evaporated in vacuo. The resulting crude was purified by silica flash chromatography (Tol→Tol:AcOEt 8:2, 1% iPrOH). The alpha product was separated from the beta counterpart in 43% of yield (294 mg).
^{1}H and ^{13}C NMR analysis corresponds to what previously reported in literature.[4]

2-Azidoethyl α-L-fucopyranoside (2a)

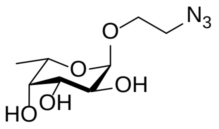

2 (107 mg, 0.32 mmol, 1 eq.) was dissolved in 3 mL of a 0.1M solution of MeONa in MeOH. Solution was stirred overnight at r.t. and neutralized with Amberlite IR-120 H$^+$. The solution was filtrated to remove the resin, then dried by rotary evaporation and with high vacuum pump. A pure white solid was obtained in quantitative yield (71 mg, 0.3 mmol).
^{1}H and ^{13}C NMR analysis corresponds to what previously reported in literature.[1]

2-Aminoethyl 2,3,4-tri-*O*-acetyl-α-L-fucopyranoside (3)

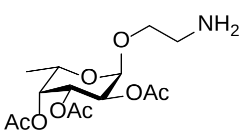

2 (216 mg, 0.6 mmol, 1 eq.), and $SnCl_2$ were dissolved in 6 mL of MeOH. 660 μL of HCl 1M (1.1 eq.) were added to the stirring solution. Reaction run at room temperature for 5 hours, until full conversion to a compound strongly staining in ninhydrine on TLC. Then, the solvent was evaporated and the crude was charged on a silica gel column. The product was eluted in a solution with a gradient of 1%→20% MeOH in DCM, with addition of 0.1-1% NEt_3 In the fractions containing residual NEt_3, the molecular ratio between the two species was calculated from the integration of relative signals in ^{1}H NMR and the yield corrected accordingly. Collectively, the compound **3** was obtained in a final yield of 99%. The disappearance on the azide was confirmed by IR.

^{1}H NMR (600 MHz, $CDCl_3$) δ: 5.45 (dd, J = 10.8, 3.3 Hz, 1 H, H-3), 5.32 – 5.26 (d, J = 3.4, 1 H, H-4), 5.15 (dd, J = 10.9, 3.6 Hz, 1 H, H-2), 5.08 (d, J = 3.6 Hz, 1 H, H-1α), 4.34 (q, J = 6.6 Hz, 1 H, H-5), 4.04 (dt, J = 11.0, 5.5 Hz, 1 H, OCH_2), 3.74 (dt, J = 10.3, 4.9 Hz, 1 H, OCH_2), 3.22 – 3.30 (m, 2 H, NCH_2), 2.15 (s, 3 H, CH_{3OAc}), 2.10 (s, 3 H, CH_{3OAc}), 1.96 (s, 3 H, CH_{3OAc}), 1.15 (d, J = 6.4 Hz, 3 H, CH_{3Fuc}).
^{1}H NMR (600 MHz, CD_3OD) δ: 5.43 (dd, J = 10.8, 3.3 Hz, 1 H, H-3), 5.31 (d, J = 3.4, 1 H, H-4), 5.13 (dd, J = 10.9, 3.6 Hz, 1 H, H-2), 5.08 (d, J = 3.6 Hz, 1 H, H-1α), 4.23 (q, J = 6.6 Hz, 1 H, H-5), 3.95 (dt, J= 10.5, 4.9 Hz, 1 H, OCH_2), 3.65 (dt, J= 10.7, 5.0 Hz, 1 H, OCH_2), 3.23 (bt, J= 5.0 Hz, 2 H, NCH_2), 2.16 (s, 3 H, CH_{3OAc}), 2.06 (s, 3 H, CH_{3OAc}), 1.97 (s, 3 H, CH_{3OAc}), 1.15 (d, J = 6.4 Hz, 3 H, CH_{3Fuc}).
^{1}H NMR (600 MHz, DMSO-d_6) δ: 5.38 (dd, J = 10.9, 3.5 Hz, 1 H, H-3), 5.17 (d, J = 3.5 Hz, 1 H, H-4), 5.00 (d, J = 3.7 Hz, 1 H, H-1α), 4.96 (dd, J = 10.9, 3.7 Hz, 1 H, H-2), 4.25 (q, J = 6.6 Hz, 1 H, H-5), 3.81 (dt, J = 11.1, 5.7 Hz, 1 H, OCH_2), 3.59 (dt, J = 10.3, 4.8 Hz, 1 H, OCH_2), 2.13 (s, 3 H, CH_{3OAc}), 2.05 (s, 3 H, CH_{3OAc}), 1.93 (s, 3 H, CH_{3OAc}), 1.05 (d, J = 6.4 Hz, 3 H, CH_{3Fuc}).

^{13}**C NMR (151 MHz, CDCl$_3$) δ:** 170.71 (1 C, C=O$_{Ac}$), 170.59 (1 C, C=OAc), 170.42 (1 C, C=OAc), 96.82 (1 C, C-1α), 71.32 (1 C, C-4), 68.22 (1 C, C-2), 68.05 (1 C, C-3), 65.27 (1 C, C-5), 64.56 (1 C, OCH$_2$), 39.23 (1 C, NCH$_2$), 20.88 (1 C, CH$_{3OAc}$), 20.78 (2 C, CH$_{3OAc}$), 16.02 (1 C, CH$_{3Fuc}$).

6.2 Spacer-elongated ligands 10, 11, 12

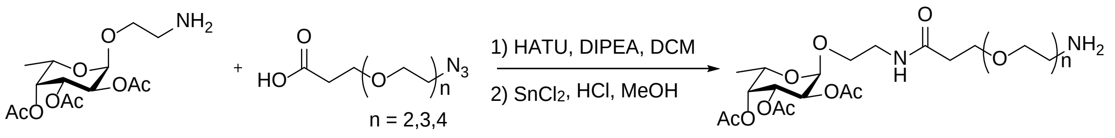

3 4'5'6 10'11,12

Scheme S1. General scheme for the synthesis of compounds **10, 11** and **12**.

(10)

^{1}**H NMR (600 MHz, CD$_3$OD) δ:** 5.34 (dd, *J* = 10.6, 3.4 Hz, 1 H, H-3), 5.27 (d, *J* = 3.4 Hz, 1 H, H-4), 5.05 (dd, *J* = 10.7, 3.7 Hz, 1 H, H-2), 5.03 (d, *J* = 3.8 Hz, 1 H, H-1α), 4.22 (q, *J* = 6.5 Hz, 1 H, H-5), 3.75 (m, 3 H, CH$_2$O), 3.72 – 3.68 (m, 2 H, CH$_2$O), 3.67 – 3.61 (m, 4 H, C*H*$_2$O), 3.53 (dt, *J* = 10.5, 5.4 Hz, 1 H, CH$_2$O), 3.45 – 3.39 (m, 2 H, CH$_2$N), 3.12 (t, *J* = 4.9 Hz, 2 H, CH$_2$N), 2.47 (t, *J* = 6.0 Hz, 2 H, CH$_2$C=O), 2.14 (s, 3 H, C*H*$_{3OAc}$), 2.05 (s, 3 H, C*H*$_{3OAc}$), 1.95 (s, 3 H, C*H*$_{3OAc}$), 1.12 (d, *J* = 6.5 Hz, 3 H C*H*$_{3Fuc}$).

^{13}**C NMR (151 MHz, CD$_3$OD) δ:** 174.29 (1 C, NC=O), 173.16 (1 C, C=O), 172.75 (1 C, C=O), 171.96 (1 C, C=O), 97.62 (1 C, C-1α), 72.49 (1 C, C-4), 71.27 (1 C, CH$_2$O), 71.24 (1 C, CH$_2$O), 69.40 (2 C, C-2, C-3), 68.12(1 C, CH$_2$O), 67.83 (1 C, CH$_2$O), 67.79 (1 C, CH$_2$O), 65.78 (1 C, C-5), 40.68 (1 C, CH$_2$N), 40.33 (1 C, CH$_2$N), 37.31 (1 C, CH$_2$C=O), 20.61 (1 C, CH$_{3OAc}$), 20.44 (1 C, CH$_{3OAc}$), (1 C, CH$_{3OAc}$), 16.15 (1 C, CH$_{3Fuc}$).

(11)

^{1}**H NMR (600 MHz, CD$_3$OD) δ:** 5.35 (dd, *J* = 10.6, 3.5 Hz, 1 H, H-3), 5.28 (dd, *J* = 3.5, 1.4 Hz, 1 H, H-4), 5.06 (dd, *J* = 10.6, 3.7 Hz, 1 H, H-2), 5.04 (d, *J* = 3.7 Hz, 1 H, H-1α), 4.23 (q, *J* = 6.6, 1.2 Hz, 1 H, H-5), 3.78 – 3.70 (m, 5 H, C*H*$_2$O), 3.70 – 3.61 (m, 8 H, C*H*$_2$O), 3.54 (ddd, *J* = 10.4, 6.0, 4.8 Hz, 1 H, C*H*$_2$O), 3.48 – 3.38 (m, 2 H, C*H*$_2$N), 3.15 – 3.11 (m, 2 H, C*H*$_2$N), 2.48 (t, *J* = 6.1 Hz, 2 H, C*H*$_2$C=O), 2.15 (s, 3 H C*H*$_{3OAc}$), 2.06 (s, 3 H, C*H*$_{3OAc}$), 1.96 (s, 3 H, C*H*$_{3OAc}$), 1.13 (d, *J* = 6.5 Hz, 3 H, C*H*$_{3Fuc}$).

^{13}**C NMR (151 MHz, CD$_3$OD) δ:** 175.04 (1 C, NC=O), 172.28 (1 C, C=O), 171.91 (1 C, C=O), 171.61 (1 C, C=O), 97.63 (1 C, C-1α), 72.51 (1 C, C-4), 71.52 (1 C, CH$_2$O), 71.36 (1 C, CH$_2$O), 71.20 (1 C, CH$_2$O), 71.15 (1 C, CH$_2$O), 69.41 (2 C, C-2, C-3), 68.19 (1 C, CH$_2$O), 67.85 (1 C, CH$_2$O), 67.81 (1 C, CH$_2$O), 65.79 (1 C, C-5), 40.65 (1 C, CH$_2$N), 40.33 (1 C, CH$_2$N), 38.04 (1 C, CH$_2$C=O), 20.68 (1 C, CH$_{3OAc}$), 20.45 (1 C, CH$_{3OAc}$), 20.41 (1 C, CH$_{3OAc}$), 16.17 (1 C, CH$_{3Fuc}$).

(12)

^{1}**H NMR (600 MHz, CD$_3$OD) δ:** 5.33 (dd, *J* = 10.5, 3.2 Hz, 1 H, H-3), 5.26 (d, *J* = 3.1 Hz, 1 H, H-4), 5.04 (t, *J* = 5.2 Hz, 1 H, H-2), 5.02 (d, *J* = 4.0 Hz, 1 H, H-1α), 4.21 (q, *J* = 6.4 Hz, 1 H, H-5), 3.74 – 3.56 (m, 17 H, C*H*$_2$O), 3.55 – 3.51 (m, 1 H, C*H*$_2$O), 3.45 – 3.38 (m, 2 H, C*H*$_2$N), 3.14 (t, *J* = 4.4 Hz, 2 H, C*H*$_2$N), 2.48 (t, *J* = 5.5 Hz, 2 H, C*H*$_2$C=O), 2.14 (s, 3 H, C*H*$_{3OAc}$), 2.05 (s, 3 H, C*H*$_{3OAc}$), 1.95 (s, 3 H, C*H*$_{3OAc}$), 1.11 (d, *J* = 6.3 Hz, 3 H, C*H*$_{3Fuc}$).

^{13}**C NMR (151 MHz, CD$_3$OD) δ:** 174.52 (1 C, NC=O), 172.18 (1 C, C=O), 171.86 (1 C, C=O), 171.59 (1 C, C=O), 97.48 (1 C, C-1α), 72.39 (1 C, C-4), 71.34 (1 C, CH$_2$O), 71.23 (1 C, CH$_2$O), 71.20 (1 C, CH$_2$O), 71.19 (1 C, CH$_2$O), 71.16 (1 C, CH$_2$O), 70.81 (1 C, CH$_2$O), 69.27 - 69.25 (2 C, C-3, C-2), 68.16 (1 C, CH$_2$O), 67.79 (1 C, CH$_2$O), 67.76 (1 C, CH$_2$O), 65.68 (1 C, C-5), 40.63 (1 C, CH$_2$N) , 40.26 (1 C, CH$_2$N), 37.32 (1 C, CH$_2$C=O), 20.79 (1 C, CH$_{3OAc}$), 20.63 (1 C, CH$_{3OAc}$), 20.45 (1 C, CH$_{3OAc}$), 16.16 (1 C, CH$_{3Fuc}$).

6.3 Cores 14, 15, 16

4-mercapto-phenyl-acetic acid ethyl ester (13)

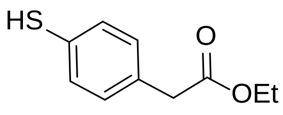

4-mercapto-phenyl-acetic acid (3.2g, 19 mmol, 1 eq.) was dissolved in 20 mL of ethanol. **Amberlite IR-120 H$^+$** resin (2g) was added to the mixture. The solution was refluxed upon stirring overnight. The resin was removed by filtration and the solvent by rotary evaporation. The crude of reaction was purified by silica flash chromatography (PE:AcOEt 9:1). Product was obtained as a colourless oil in 91% of yield.

^{1}H NMR (600 MHz, CDCl$_3$) δ: 7.23 (d, J = 8.2 Hz, 2 H, H$_{Ar}$), 7.15 (d, J = 8.2 Hz, 2 H, H$_{Ar}$), 4.14 (q, J = 7.1 Hz, 2 H, OCH_2), 3.55 (s, 2 H, CH_{2benz}), 3.43 (s, 1 H, S*H*), 1.24 (t, J = 7.2 Hz, 3 H, CH_3).
^{13}C NMR (151 MHz, CDCl$_3$) δ: 171.48 (1 C, C=O), 131.82 (1 C, C$_q$), 130.10 (2 C, CH$_{Ar}$), 129.81 (2 C, CH$_{Ar}$), 129.44 (1 C, C$_q$), 61.03 (1 C, OCH$_2$), 40.92 (1 C, CH$_{2benz}$), 14.28 (1 C, CH$_3$).

Tris-1,3,5-(4-(sulfanylmethyl) phenylacetic acid) benzene (14)

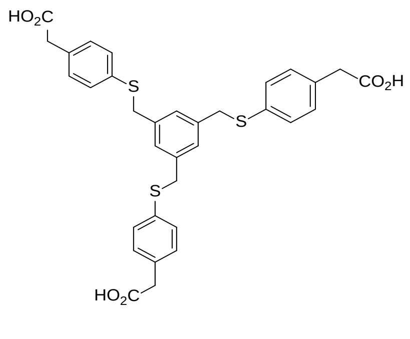

Reaction followed the general procedure, starting from **13** and **1,3,5-tris(bromomethyl)benzene**. The ester was purified by silica gel chromatography, using as eluent a mixture Hex:EtOAc 9:1→7:3.
Acid **14** was obtained as a white powder in 41% of yield over 2 steps.

^{1}H NMR (600 MHz, CD$_3$OD) δ: 7.16 (s, 12 H, CH_{SAr}), 7.02 (s, 3 H, CH_{Ar}), 3.97 (s, 6 H, SCH_2Ph), 3.48 (s, 6 H, COCH_2Ph).
^{13}C NMR (151 MHz, CD$_3$OD) δ: 139.59 (3 C, Cq), 138.00 (3 C, Cq), 134.49 (3 C, Cq), 131.46 (6 C, CH$_{SAr}$), 130.79 (6 C, CH$_{SAr}$), 129.22 (3 C, CH$_{Ar}$), 45.88 (3 C, COCH$_2$Ph), 39.88 (3 C, SCH$_2$Ph).

Tetrakis-1,2,4,5-((4-(sulfanylmethyl) phenylacetic acid) benzene (15)

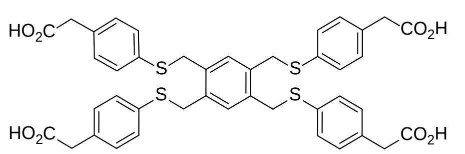

Reaction procedures followed the general procedure, starting from **13** and **1,2,4,5-tetrakis(bromomethyl)benzene**. The eluent used for flash chromatography was a mixture of PE:Tol:EtOAc 7:1:2. Acid **15** was obtained as a white powder in 87% of yield over 2 steps.

^{1}H NMR (600 MHz, CD$_3$OD) δ: 7.21 (dd, J = 8.3 Hz, 16 H, CH_{SAr}), 6.99 (s, 2 H, CH_{Ar}), 4.08 (s, 8 H, SCH_2Ph), 3.46 (s, 8 H, COCH_2Ph).
^{13}C NMR (151 MHz, CD$_3$OD) δ: 138.27 (4 C, Cq), 136.61 (4 C, Cq), 134.70 (4 C, Cq), 134.00 (2 C, CH$_{Ar}$), 132.15 (8 C, CH$_{SAr}$), 131.08 (8 C, CH$_{SAr}$), 45.71 (4 C, COCH$_2$Ph), 37.75 (4 C, SCH$_2$Ph).

Hexakis-(4-(sulfanylmethyl) phenylacetic acid) benzene (16)

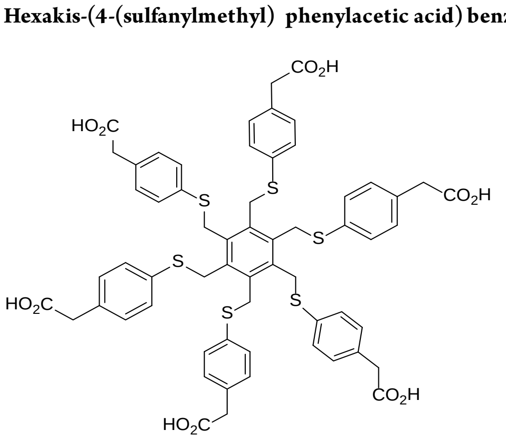

Reaction procedures followed the general procedure, starting from **13** and **hexakis(bromomethyl)benzene**. The eluent used for flash chromatography was a mixture of PE:Tol:EtOAc 4:4:2 + 1% iPrOH. Ethyl ester **16** was obtained as a white solid in 61% of yield over 2 steps.

^{1}H NMR (600 MHz, CD$_3$OD) δ: 7.29 – 7.10 (dd, 12 H, CH_{SAr}), 4.16 (s, 12 H, SCH_2Ph), 3.59 (s, 12 H, COCH_2Ph).
^{13}C NMR (151 MHz, CD$_3$OD) δ: 137.35 (6 C, Cq), 135.94 (6 C, Cq), 135.16 (6 C, Cq), 132.15 (12 C, CH$_{SAr}$), 131.41 (12 C, CH$_{SAr}$), 42.12 (6 C, COCH2Ph), 34.96 (6 C, SCH$_2$Ph).

6.4 Multivalent fucosides

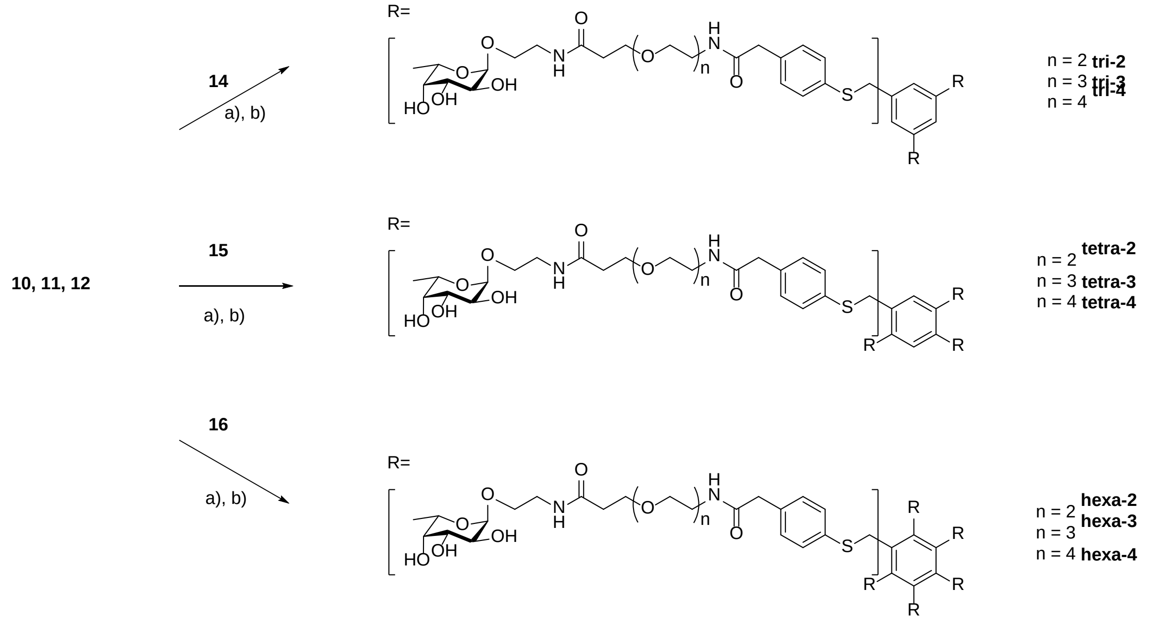

Scheme S2: Reagents and conditions: a) HATU, DIPEA, DMF; b) MeONa/MeOH.

(tri-2)

^{1}H NMR (600 MHz, CD_3OD) δ: 7.17 (s, 12 H, CH_{SAr}), 7.01 (s, 3 H, CH_{Ar}), 4.75 (d, J = 2.2 Hz, 3 H, H-1α), 3.98 (s, 6 H, SCH_2Ph), 3.92 (q, J = 6.6 Hz, 3 H, H-5), 3.75 – 3.73 (m, 6 H, H-2, H-3), 3.72 (t, J = 5.9 Hz, 9 H, CH_2O), 3.65 (d, J = 1.9 Hz, 3 H, H-4), 3.59 – 3.52 (m, 15 H, CH_2O, CH_2N), 3.50 (t, J = 5.5 Hz, 6 H, CH_2O), 3.47 (s, 6 H, $COCH_2Ph$), 3.43 (ddd, J = 10.8, 7.3, 3.9 Hz, 3 H, CH_2O), 3.34 (t, J = 5.4 Hz, 6 H, CH_2N), 3.27- 3.31 (m, 3 H, CH_2N), 2.44 (t, J = 6.1 Hz, 6 H, CH_2CO), 1.19 (d, J = 6.5 Hz, 9 H, CH_{3Fuc}).
^{13}C NMR (151 MHz, CD_3OD) δ: 173.90 (6 C, C=O), 139.56 (3 C, C_{qAr}), 135.71 (3 C, C_{qSAr}), 135.54 (3 C, C_{qSAr}), 131.71 (6 C, CH_{SAr}), 130.70 (6 C, CH_{SAr}), 129.27 (3 C, CH_{Ar}), 100.56 (3 C, C-1α), 73.62 (3 C, C-4), 71.64 (3 C, C-2), 71.32 (3 C, CH_2O), 71.24 (3 C, CH_2O), 70.50 (3 C, CH_2O), 70.02 (3 C, C-3), 68.25 (3 C, CH_2O), 67.79 (3 C, CH_2O), 67.68 (3 C, C-5), 43.32 (3 C, $COCH_2Ph$), 40.61 (3 C, CH_2N), 40.30 (3 C, CH_2N), 39.52 (3 C, SCH_2Ph), 37.62 (3 C, $COCH_2$), 16.72 (3 C, CH_{3Fuc}).
HRMS (ESI+, Q-Tof) m/z: calculated for $C_{78}H_{115}N_6O_{27}S_3$ $[M+H]^+$=1663.6972, found: 1663.6888; calcd for $C_{78}H_{114}N_6NaO_{27}S_3$ $[M+Na]^+$=1685.6792, found 1685.6819; $C_{78}H_{114}N_6Na_2O_{27}S_3$ $[M+2Na]^{2+}$=854.3345, found 854.3350.
HPLC purity (254nm): 95%.

(tri-3)

^{1}H NMR (600 MHz, CD_3OD) δ: 7.17 (s, 12 H, CH_{SAr}), 7.01 (s, 3 H, CH_{Ar}), 4.75 (d, *J* = 1.7 Hz, 3 H, H-1α), 3.98 (s, 6 H, SCH_2Ph), 3.93 (dq, *J* = 6.6, 1.3 Hz, 3 H, H-5), 3.76 – 3.72 (m, 6 H, H-2, H-3), 3.72 (t, *J* = 6.1 Hz, 9 H, CH_2O), 3.65 (q, *J* = 1.4 Hz, 3 H, H-4), 3.62 – 3.57 (m, 18 H, CH_2O), 3.57 – 3.54 (m, 6 H, CH_2O), 3.53 – 3.51 (dd, *J* = 6.0, 4.1 Hz, 3 H, CH_2N), 3.50 (t, *J* = 5.5 Hz, 6 H, CH_2O), 3.46 (s, 6 H, $COCH_2Ph$), 3.45 – 3.41 (m, 3 H, CH_2O), 3.34 (t, *J* = 5.5 Hz, 6 H, CH_2N), 3.30 – 3.25 (m, 3 H, CH_2N), 2.45 (t, *J* = 6.2 Hz, 6 H, CH_2CO), 1.20 (d, *J* = 6.6 Hz, 9 H, CH_{3Fuc}).
^{13}C NMR (151 MHz, CD_3OD) δ: 173.95 (1 C, C=O), 139.57 (3 C, C_{qAr}), 135.71 (3 C, C_{qSAr}), 135.53 (3 C, C_{qSAr}), 131.70 (6 C, C_{qSAr}), 130.70 (6 C, CH_{SAr}), 129.27 (3 C, CH_{Ar}), 100.56 (3 C, C-1α), 73.62 (3 C, C-4), 71.64 (3 C, C-2), 71.57 (3 C, CH_2O), 71.46 (3 C, CH_2O), 71.30 (3 C, CH_2O), 71.28 (3 C, CH_2O), 70.48 (3 C, CH_2O), 70.02 (3 C, C-3), 68.25 (3 C, CH_2O), 67.79 (3 C, CH_2O), 67.68 (3 C, C-5), 43.33 (3 C, $COCH_2Ph$), 40.60 (3 C, CH_2N), 40.28 (3 C, CH_2N), 39.51 (3 C, SCH_2Ph), 37.65 (3 C, $COCH_2$), 16.71 (3 C, CH_{3Fuc}).
HRMS (ESI+, Q-Tof) m/z: calculated for $C_{84}H_{127}N_6O_{30}S_3$ $[M+H]^+$=1795.7759, found: 1795.7740; calcd for $C_{84}H_{126}N_6NaO_{30}S_3$ $[M+Na]^+$= 1817.7578, found: 1817.7584; calcd for $C_{84}H_{126}N_6Na_2O_{30}S_3$ $[M+2Na]^{2+}$= 920.3738, found: 920.3739.
HPLC purity (254nm): 98%.

(tri-4)

^{1}H NMR (600 MHz, CD_3OD) δ:7.18 (s, 12 H, CH_{SAr}), 7.02 (s, 3 H, CH_{Ar}), 4.76 (d, J = 1.9 Hz, 3 H, H-1α), 3.98 (s, 6 H, SCH_2Ph), 3.93 (dq, J = 6.7 Hz, 3 H, H-5), 3.77 – 3.73 (m, 6 H, H-2, H-3), 3.71 (t, *J* = 6.2 Hz, 9 H, CH_2O), 3.65 (d, *J* = 1.5 Hz, 3 H, H-4), 3.62 – 3.55 (m, 36 H, CH_2O), 3.54 – 3.51 (m, 3 H, CH_2N), 3.51 (t, *J* = 5.4 Hz, 6 H, CH_2O), 3.46 (s, 6 H, $COCH_2Ph$), 3.45 – 3.41 (m, 3 H, CH_2O), 3.34 (t, *J* = 5.4 Hz, 6 H, CH_2N), 3.30 – 3.27 (m, 3 H, CH_2N), 2.44 (t, *J* = 6.2 Hz, 6 H, CH_2CO), 1.20 (d, *J* = 6.6 Hz, 9 H, CH_{3Fuc}).
^{13}C NMR (151 MHz, CD_3OD) δ: 139.57 (3 C, C_{qAr}), 135.72 (3 C, C_{qSAr}), 135.54 (3 C, C_{qSAr}), 131.69 (6 C, C_{qSAr}), 130.71 (6 C, CH_{SAr}), 129.27 (3 C, CH_{Ar}), 100.56 (3 C, C-1α), 73.61 (3 C, C-4), 71.64 (3 C, C-2), 71.56 (6 C, CH_2O), 71.53 (3 C, CH_2O), 71.45

(3 C, CH_2O), 71.33 (3 C, CH_2O), 71.27 (3 C, CH_2O), 70.47 (3 C, CH_2O), 70.02(3 C, C-3), 68.25 (3 C, CH_2O), 67.79 (3 C, CH_2O), 67.68 (3 C, C-5), 43.34 (3 C, $COCH_2Ph$), 40.61 (3 C, CH_2N), 40.28 (3 C, CH_2N), 39.51 (3 C, SCH_2Ph), 37.66 (3 C, $COCH_2$), 16.71 (3 C, CH_{3Fuc}).
HRMS (ESI+, Q-Tof) m/z: calculated for $C_{90}H_{139}N_6O_{33}S_3$ $[M+H]^+$=1927.8545, found: 1927.8587; calcd for $C_{90}H_{138}N_6NaO_{33}S_3$ $[M+Na]^+$=1949.8365, found: 1949.8389; calcd for $C_{90}H_{138}N_6Na_2O_{33}S_3$ $[M+2Na]^{2+}$= 986.4131, found: 986.9167.
HPLC purity (254nm): 95%.

(tetra-2)

^{1}H NMR (600 MHz, CD_3OD) δ: 7.23 – 7.17 (m, 16 H, CH_{SAr}), 6.91 (s, 2 H, CH_{SAr}), 4.76 (bd, *J* = 1.8 Hz, 4 H, H-1α), 4.05 (s, 8 H, SCH_2Ph), 3.85 (q, *J* = 6.7 Hz, 4 H, H-5), 3.76 – 3.70 (m, 20 H, H-2, H-3, CH_2O), 3.65 (d, J = 1.8 Hz, 4 H, H-4), 3.59 – 3.54 (m, 16 H, CH_2O), 3.54 – 3.47 (m, 12 H, CH_2O, CH_2N), 3.49 (s, 8 H, $COCH_2Ph$), 3.43 (ddd, J = 10.7, 7.3, 3.9 Hz, 4 H, CH_2O), 3.35 (t, J = 5.5 Hz, 8 H, CH_2N), 3.30 – 3.27 (m, 4 H, CH_2N), 2.44 (t, J = 6.1 Hz, 8 H, $COCH_2$), 1.19 (d, J = 6.5 Hz, 12 H, CH_{3Fuc}).
^{13}C NMR (151 MHz, CD_3OD) δ: 170.90 (4 C, C=O), 170.55 (4 C, C=O), 136.29 (4 C, C_{qAr}), 136.00 (4 C, C_{qSAr}), 135.54 (4 C, C_{qSAr}), 133.80 (2 C, CH_{Ar}), 132.29 (8 C, CH_{SAr}), 130.82 (8 C, CH_{SAr}), 100.56 (4 C, C-1α), 73.61 (4 C, C-4), 71.65 (4 C, C-2), 71.31 (4 C, CH_2O), 71.24 (4 C, CH_2O), 70.52 (4 C, CH_2O), 70.02 (4 C, C-3), 68.26 (4 C, CH_2O), 67.80 (4 C, CH_2O), 67.69 (4 C, C-5), 43.36 (4 C, $COCH_2Ph$), 40.63 (4 C, CH_2N), 40.31 (4 C, CH_2N), 37.63 (4 C, SCH_2Ph), 37.28 (4 C, $COCH_2$), 16.73 (4 C, CH_{3Fuc}).
HRMS (ESI+, Q-Tof) m/z: calculated for $C_{102}H_{151}N_8O_{36}S_4$ $[M+H]^+$=2191.9114, found: 2191.9246; calcd for $C_{102}H_{150}N_8NaO_{36}S_4$ $[M+Na]^+$=2213.8933, found: 2213.9001; calcd for $C_{102}H_{150}N_8Na_2O_{36}S_4$ $[M+2Na]^{2+}$=1118.4416, found 1118.4475.
HPLC purity (254nm): 96%.

(tetra-3)

^{1}H NMR (600 MHz, CD_3OD) δ: 7.23 – 7.17 (m, 16 H, CHS_{Ar}), 6.92 (s, 2 H, CH_{SAr}), 4.76 (bd, *J* = 1.6 Hz, 4 H, H-1α), 4.05 (s, 8 H, SCH_2Ph), 3.93 (q, *J* = 6.7 Hz, 4 H, H-5), 3.76 – 3.73 (m, 8 H, H-2, H-3), 3.72 (t, *J* = 6.1 Hz, 12 H, CH_2O), 3.65 (d, *J* = 1.7 Hz, 4 H, H-4), 3.62 – 3.55 (m, 33 H, CH_2O), 3.51 (m, 12 H, CH_2O, CH_2N), 3.49 (s, 8 H, $COCH_2Ph$), 3.43 (ddd, *J* = 10.2, 7.2, 4.0 Hz, 4 H, CH_2O), 3.35 (t, *J* = 5.4 Hz, 8 H, CH_2N), 3.30 – 3.27 (m, 3 H, CH_2N), 2.45 (t, *J* = 6.2 Hz, 8 H, $COCH_2$), 1.20 (d, *J* = 6.6 Hz, 12 H, CH_{3Fuc}).
^{13}C NMR (151 MHz, CD_3OD) δ: 173.96 (4 C, C=O), 173.83 (4 C, C=O), 136.30 (4 C, C_{qAr}), 136.00 (4 C, C_{qSAr}), 135.55 (4 C, C_{qSAr}), 133.82 (2 C, CH_{Ar}), 132.27 (8 C, CH_{SAr}), 130.84 (8 C, CH_{SAr}), 100.55 (4 C, C-1α), 73.61 (4 C, C-4), 71.63 (4 C, C-2), 71.57 (4 C, CH_2O), 71.45 (4 C, CH_2O), 71.30 (8 C, CH_2O), 70.49 (4 C, CH_2O), 70.01 (4 C, C-3), 68.25 (4 C, CH_2O), 67.79 (4 C, CH_2O), 67.68 (4 C, C-5), 43.37 (4 C, $COCH_2Ph$), 40.62 (4 C, CH_2N), 40.29 (4 C, CH_2N), 37.65 (4 C, SCH_2Ph), 37.26 (4 C, $COCH_2$), 16.73 (4 C, CH_{3Fuc}).
HRMS (ESI+, Q-Tof) m/z: calculated for $C_{110}H_{167}N_8O_{40}S_4$ $[M+H]^+$=2368.0162, found: 2368.0275; calcd for $C_{110}H_{166}N_8NaO_{40}S_4$ $[M+Na]^+$=2389.9982, found: 2390.0134; clcd for $C_{110}H_{166}N_8Na_2O_{40}S_4$ $[M+2Na]^{2+}$=1.206.494, found: 1206.4978; calcd for $C_{110}H_{168}N_8O_{40}S_4$ $[M+2H]^{2+}$=1184.5121, found: 1184.5142.
HPLC purity (254nm): 97%.

(tetra-4)

^{1}H NMR (600 MHz, CD_3OD) δ: 7.23 – 7.18 (m, 16 H, CHS_{Ar}), 6.93 (s, 2 H, CH_{SAr}), 4.76 (bd, *J* = 2.1 Hz, 4 H, H-1α), 4.06 (s, 8 H, SCH_2Ph), 3.93 (q, *J* = 6.6 Hz, 4 H, H-5), 3.76 – 3.72 (m, 8 H, H-2, H-3), 3.71 (t, *J* = 6.2 Hz, 12 H, CH_2O), 3.65 (d, *J* = 1.9 Hz, 4 H, H-4), 3.63 – 3.55 (m, 49 H, CH_2O), 3.51 (m, 12 H, CH_2O, CH_2N), 3.49 (s, 8 H, $COCH_2Ph$), 3.44 (ddd, *J* = 10.6, 7.2, 4.0 Hz, 4 H, CH_2O), 3.35 (dd, *J* = 6.3, 4.6 Hz, 8 H, CH_2N), 3.30 – 3.27 (m, 3 H, CH_2N), 2.44 (t, *J* = 6.2 Hz, 8 H, $COCH_2$), 1.20 (d, *J* = 6.6 Hz, 12 H, CH_{3Fuc}).
^{13}C NMR (151 MHz, CD_3OD) δ: 174.00 (4 C, C=O), 173.81 (4 C, C=O), 136.30 (4 C, C_{qAr}), 136.00 (4 C, C_{qSAr}), 135.55 (4 C, C_{qSAr}), 133.82 (2 C, CH_{Ar}), 132.24 (8 C, CH_{SAr}), 130.85 (8 C, CH_{SAr}), 100.55 (4 C, C-1α), 73.60 (4 C, C-4), 71.63 (4 C, C-2), 71.56 (8 C, CH_2O), 71.52 (4 C, CH_2O), 71.44 (4 C, CH_2O), 71.32(4 C, CH_2O), 71.27 (4 C, CH_2O), 70.48 (4 C, CH_2O), 70.01 (4 C, C-3), 68.25(4 C, CH_2O), 67.80 (4 C, CH_2O), 67.68 (4 C, C-5), 43.37 (4 C, $COCH_2Ph$), 40.62 (4 C, CH_2N), 40.28 (4 C, CH_2N), 37.65 (4 C, SCH_2Ph), 37.25 (4 C, $COCH_2$), 16.73 (4 C, CH_{3Fuc}).
HRMS (ESI+, Q-Tof) m/z: calculated for $C_{118}H_{183}N_8O_{44}S_4$ $[M+H]^+$=2544.1211, found: 2544.1148; calcd for $C_{118}H_{182}N_8NaO_{44}S_4$ $[M+Na]^+$=2566.1030, found: 2566.0985; calcd for $C_{118}H_{182}N_8Na_2O_{44}S_4$ $[M+2Na]^{2+}$=1294.5464, found: 1295.0500.
HPLC purity (254nm): 96%.

(hexa-2)

^{1}H NMR (600 MHz, CD_3OD) δ: 7.26 (d, *J* = 8.2 Hz, 12 H, CHS_{Ar}), 7.22 (d, *J* = 8.2 Hz, 12 H, CHS_{Ar}), 4.76 (bd, *J* = 1.7 Hz, 6 H, H-1α), 4.17 (s, 12 H, SCH_2Ph), 3.92 (q, *J* = 6.6 Hz, 6 H, H-5), 3.78 – 3.69 (m, 30 H, H-2, H-3, CH_2O), 3.65 (bd, *J* = 1.9 Hz, 6 H, H-4), 3.59 – 3.49 (m, 54 H, $COCH_2Ph$, CH_2O, CH_2N), 3.43 (ddd, *J* = 10.7, 7.2, 3.9 Hz, 6 H, CH_2O), 3.36 (t, *J* = 5.4 Hz, 12 H, CH_2N), 3.31 – 3.27 (m, 6 H, CH_2N), 2.44 (t, *J* = 6.1 Hz, 12 H, $COCH_2$), 1.19 (d, *J* = 6.6 Hz, 18 H, CH_{3Fuc}).
^{13}C NMR (151 MHz, CD_3OD) δ: 173.90 (6 C, C=O), 173.75 (6 C, C=O), 137.30 (6 C, C_{qAr}), 136.55 (6 C, C_{qSAr}), 135.19 (6 C, C_{qSAr}), 132.19 (12 C, CH_{SAr}), 131.22 (12 C, CH_{SAr}), 100.54 (6 C, C-1α), 73.60 (6 C, C-4), 71.63 (6 C, C-2), 71.31 (6 C, CH_2O), 71.22

(6 C, CH_2O), 70.52 (6 C, CH_2O), 70.00 (6 C, C-3), 68.26 (6 C, CH_2O), 67.79 (6 C, CH_2O), 67.68 (6 C, C-5), 43.37 (6 C, $COCH_2Ph$), 40.64 (6 C, CH_2N), 40.31 (6 C, CH_2N), 37.62 (6 C, $COCH_2$), 34.91 (6 C, SCH_2Ph), 16.75 (6 C, CH_{3Fuc}).
HRMS (ESI+, Q-Tof) m/z: calculated for $C_{150}H_{223}N_{12}O_{54}S_6$ $[M+2H]^{2+}$=1624.16985, found: 1624.6711; calcd for $C_{150}H_{222}N_{12}Na_2O_{54}S_6$ $[M+2Na]^{2+}$=1646.6557, found: 1646.6544.
HPLC purity (254nm): 98%.

(hexa-3)

^{1}H NMR (600 MHz, CD_3OD) δ: 7.26 (d, *J* = 8.3 Hz, 12 H, CHS_{Ar}), 7.23 (d, *J* = 8.3 Hz, 12 H, CHS_{Ar}), 4.76 (bd, *J* = 1.7 Hz, 6 H, H-1α), 4.19 (s, 12 H, SC*H*$_2$Ph), 3.92 (q, *J* = 6.6 Hz, 6 H, H-5), 3.74 -3.69 (m, 30 H, H-2, H-3, C*H*$_2$O), 3.65 (bd, *J* = 1.6 Hz, 6 H, H-4), 3.61 – 3.55 (m, 48 H, C*H*$_2$O), 3.55 – 3.48 (m, 30 H, COC*H*$_2$Ph, C*H*$_2$O, C*H*$_2$N), 3.43 (ddd, *J* = 10.6, 7.2, 4.0 Hz, 6 H, C*H*$_2$O), 3.37 (t, *J* = 5.4 Hz, 12 H, C*H*$_2$N), 3.28 – 3.31 (m, 6 H, C*H*$_2$N), 2.44 (t, *J* = 6.2 Hz, 12 H, COC*H*$_2$), 1.19 (d, *J* = 6.6 Hz, 18 H, C*H*$_{3Fuc}$).
^{13}C NMR (151 MHz, CD_3OD) δ: 173.91 (6 C, C=O), 173.67 (6 C, C=O), 137.46 (6 C, C_{qAr}), 136.57 (6 C, C_{qSAr}), 135.20 (6 C, C_{qSAr}), 132.20 (12 C, CH_{SAr}), 131.23 (12 C, CH_{SAr}), 100.58 (6 C, C-1α), 73.62 (6 C, C-4), 71.66 (6 C, C-2), 71.60 (6 C, CH_2O), 71.47 (6 C, CH_2O), 71.32 (6 C, CH_2O), 71.31 (6 C, CH_2O), 70.52 (6 C, CH_2O), 70.04 (6 C, C-3), 68.27 (6 C, CH_2O), 67.81 (6 C, CH_2O), 67.69 (6 C, C-5), 43.42 (6 C, $COCH_2Ph$), 40.67 (6 C, CH_2N), 40.30 (6 C, CH_2N), 37.69 (6 C, $COCH_2$), 35.56 (6 C, SCH_2Ph), 16.75 (6 C, CH_{3Fuc}).
HRMS (ESI+, Q-Tof) m/z: calculated for $C_{162}H_{248}N_{12}O_{60}S_6$ $[M+2H]^{2+}$=1756.7524, found: 1756.7491; calcd $C_{162}H_{246}N_{12}Na_2O_{60}S_6$ $[M+2Na]^{2+}$=1778.73435, found: 1778.7306.
HPLC purity (254nm): 98%.

(hexa-4)

^{1}H NMR (600 MHz, CD_3OD) δ: 7.27 (d, J = 8.3 Hz, 12 H, CHS_{Ar}), 7.23 (d, J = 8.2 Hz, 12 H, CHS_{Ar}), 4.76 (bd, J = 1.8 Hz, 6 H, H-1α), 4.20 (s, 12 H, SC*H*$_2$Ph), 3.93 (dt, J = 7.2, 6.0 Hz, 6 H, H-5), 3.76 – 3.68 (m, 30 H, H-2, H-3, C*H*$_2$O), 3.65 (bd, J = 1.5 Hz, 6 H, H-4), 3.63 – 3.55 (m, 72 H, C*H*$_2$O), 3.55 – 3.49 (m, 30 H, COC*H*$_2$Ph, C*H*$_2$O, C*H*$_2$N), 3.44 (ddd, J = 10.5, 7.1, 4.0 Hz, 6 H, C*H*$_2$O), 3.37 (t, J = 5.4 Hz, 12 H, C*H*$_2$N), 3.29 (dt, J = 7.1, 3.4 Hz, 6 H, C*H*$_2$N), 2.44 (t, J = 6.2 Hz, 12 H, COC*H*$_2$), 1.20 (d, J = 6.5 Hz, 18 H, C*H*$_{3Fuc}$).
^{13}C NMR (151 MHz, CD_3OD) δ: 173.96 (6 C, C=O), 173.68 (6 C, C=O), 137.31 (6 C, C_{qAr}), 136.57 (6 C, C_{qSAr}), 135.19 (6 C, C_{qSAr}), 132.13 (12 C, CH_{SAr}), 131.25(12 C, CH_{SAr}), 100.55 (6 C, C-1α), 73.60 (6 C, C-4), 71.63 (6 C, C-2), 71.57 (12 C, CH_2O), 71.53 (6 C, CH_2O), 71.44 (6 C, CH_2O), 71.32 (6 C, CH_2O), 71.27 (6 C, CH_2O), 70.49 (6 C, CH_2O), 70.01 (6 C, C-3), 68.25 (6 C, CH_2O), 67.80 (6 C, CH_2O), 67.68 (6 C, C-5), 43.39 (6 C, $COCH_2Ph$), 40.65 (6 C, CH_2N), 40.28 (6 C, CH_2N), 37.66 (6 C, $COCH_2$), 34.91 (6 C, SCH_2Ph), 16.75 (6 C, CH_{3Fuc}).
HRMS (MALDI, DHB and CHCA matrix) m/z: calculated for $C_{172}H_{270}N_{12}Na_2O_{65}S_6$ $[M+4H+2Na]^{6+}$=630.2719, found: 629.5241; calcd for $C_{172}H_{266}N_{12}Na_6O_{65}S_6$ $[M+6Na]^{6+}$=644.9265, found: 645.4750.
HPLC purity (254nm): 99%.

6.5 Polyglycerol-fucose conjugate (hPG-20)

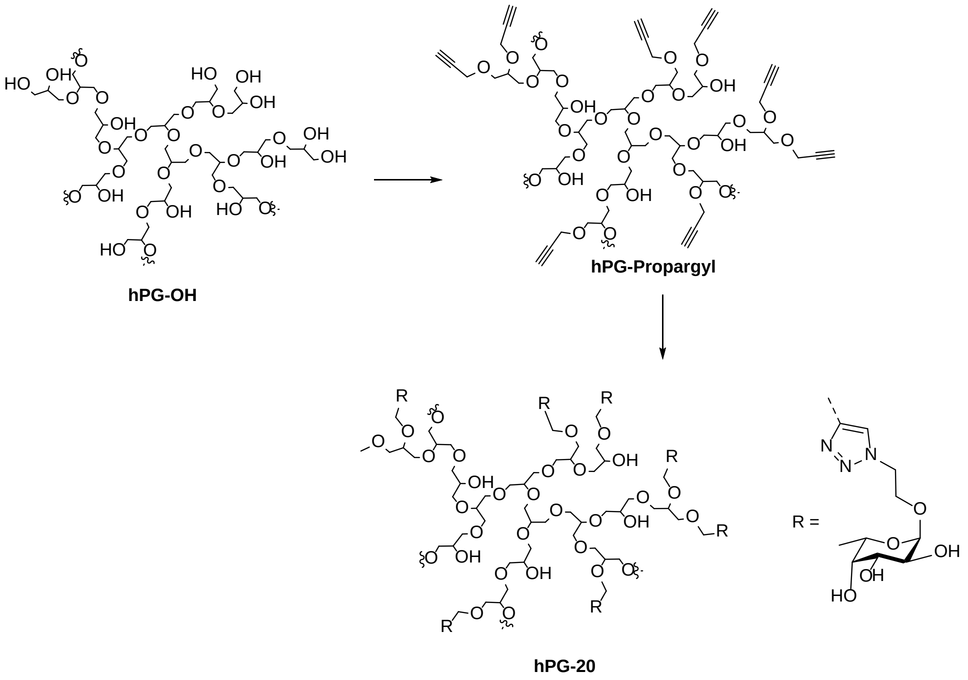

Scheme S3. Synthesis of **hPG-20**.

^{1}H NMR (600 MHz, D_2O) δ: 8.14 (d, J = 7.7 Hz, 1 H, triazole), 4.72 (s, Fuc, H-1), 4.67 (s, Fuc, OCH_2CH_2), 4.07 – 3.40 (m, hPG C*H*, hPG CH_2, Fuc: H-2, H-3, H-4, OCH_2CH_2N), 3.12 – 3.05 (m, Fuc, H-5), 1.03 – 0.98 (m, Fuc, H-6).
^{13}C NMR (151 MHz, D_2O) δ: 125.69 (C-triazole), 98.00 (Fuc, C-1), 77.95, 72.06, 71.51, 70.65, 70.36, 69.40, 68.81, 67.73, 66.43 (Fuc, C-5), 65.85 (Fuc, CH_2CH_2N), 62.60, 50.10, 15.25 (Fuc, C-6).

7. Characterization of final compounds

^{1}H NMR 3

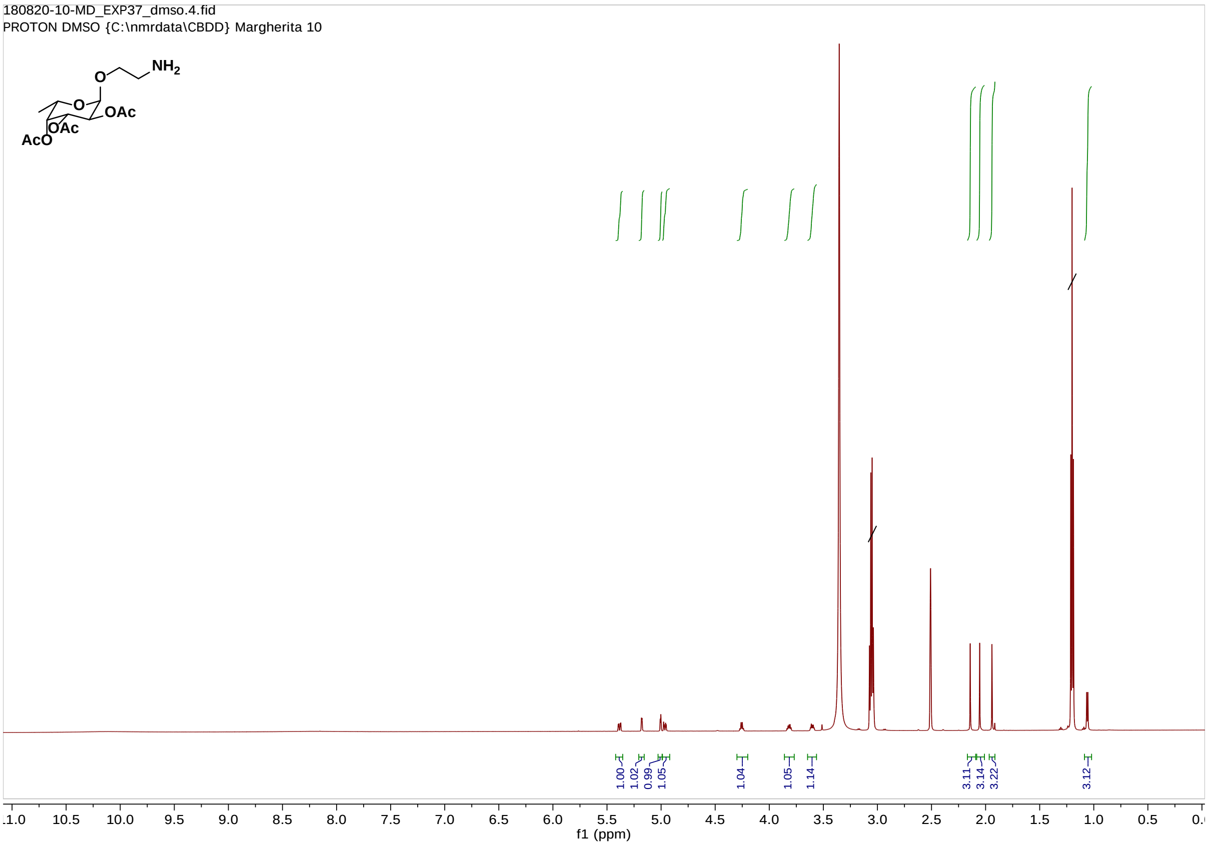

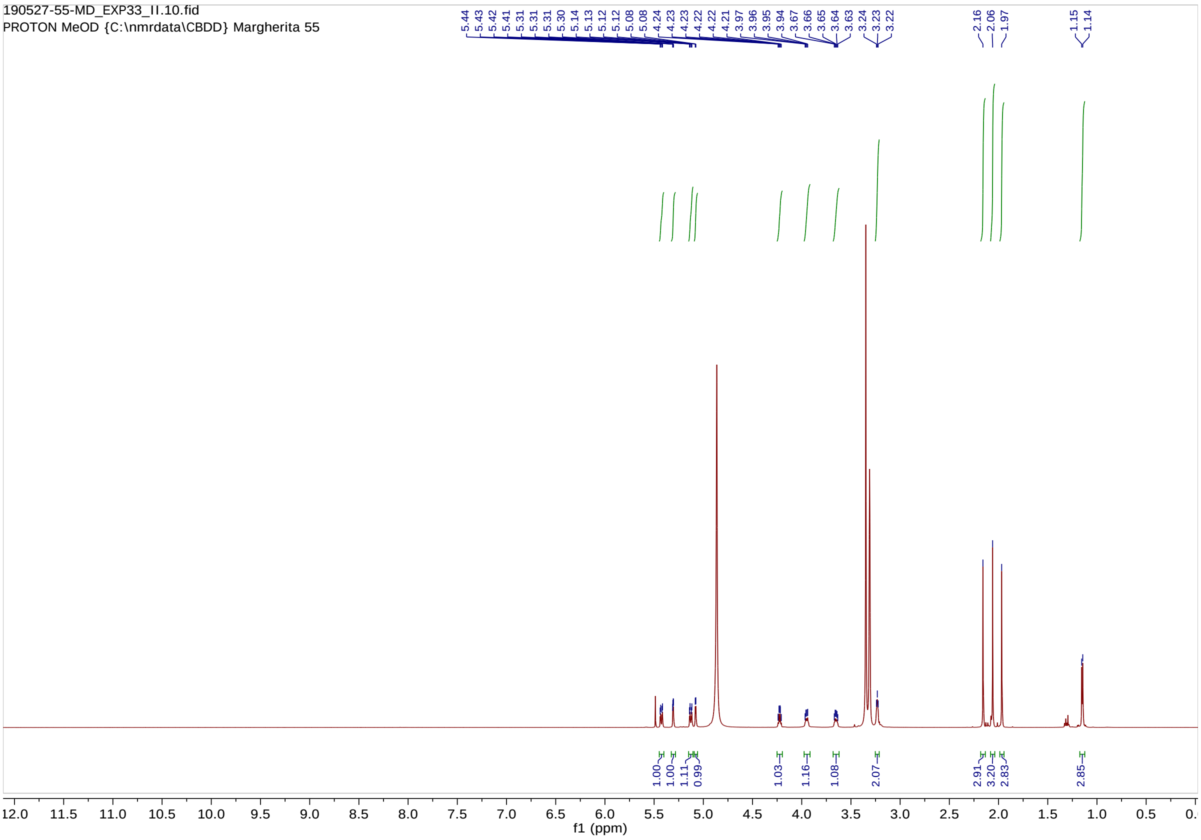

^{1}H NMR tri-2

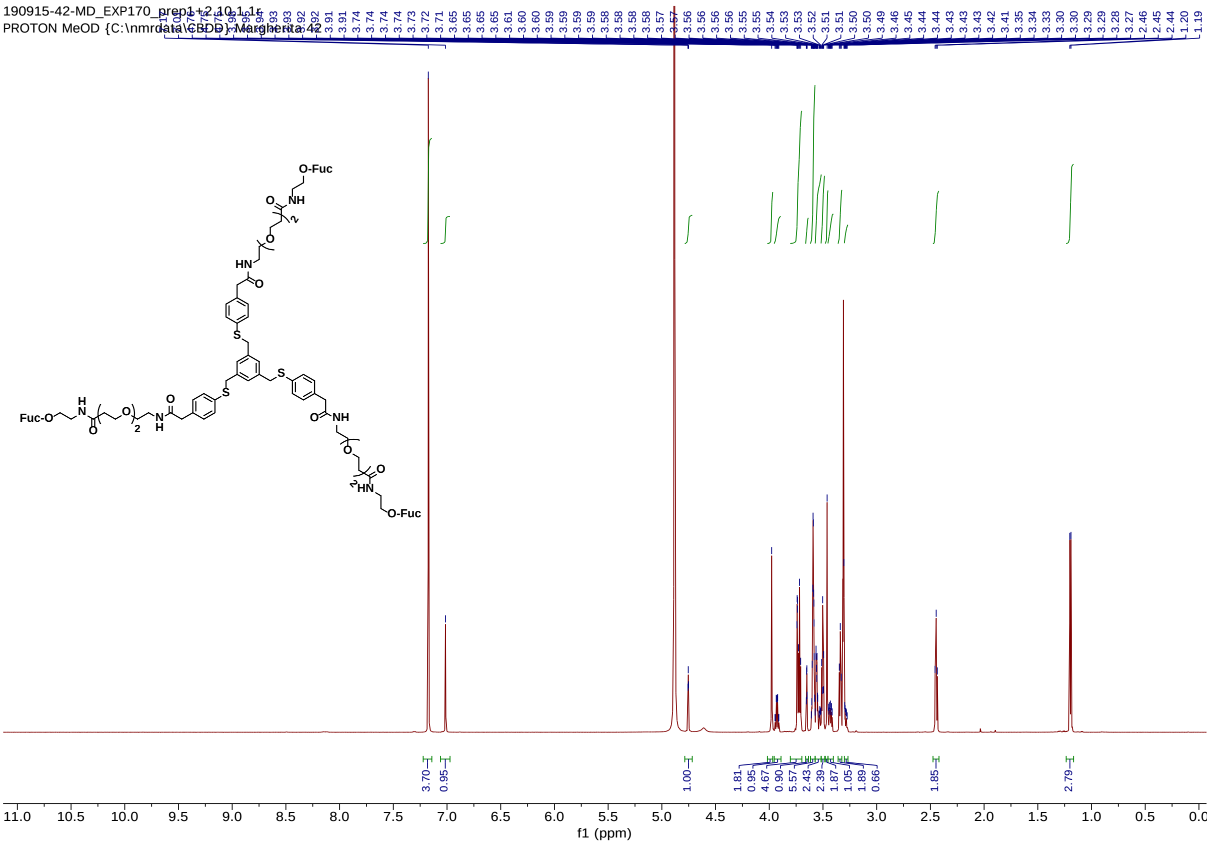

Analytical HPLC tri-2 (blank subtracted)

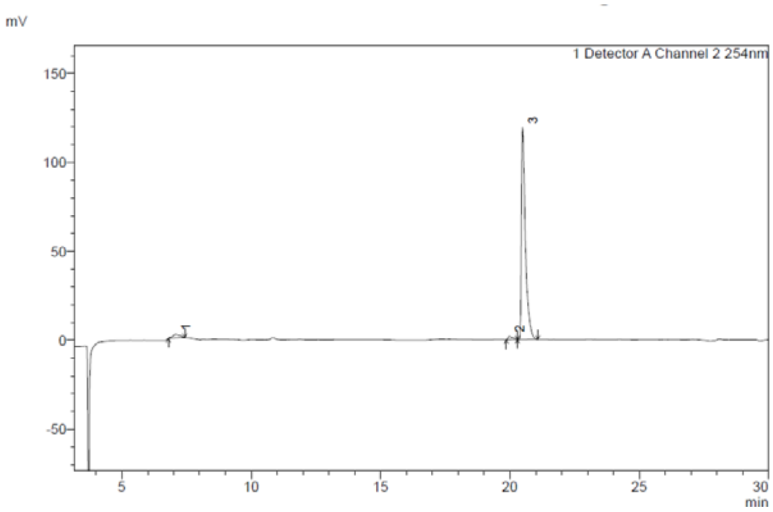

Detector A Channel 2 254nm

Peak#	Area	Area%
1	43031	3.185
2	24922	1.845
3	1283056	94.970
Total	1351010	100.000

^{1}H NMR tetra-2

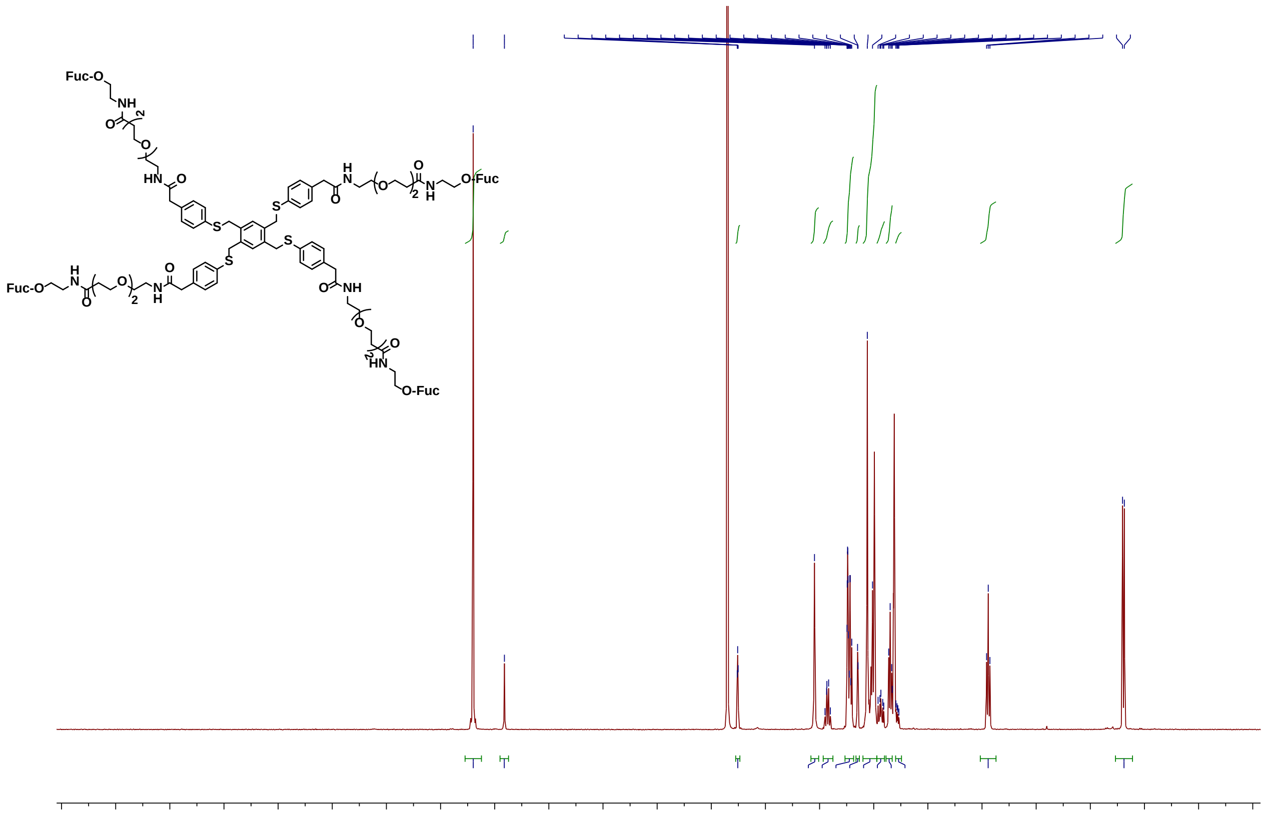

Analytical HPLC tetra-2 (blank subtracted)

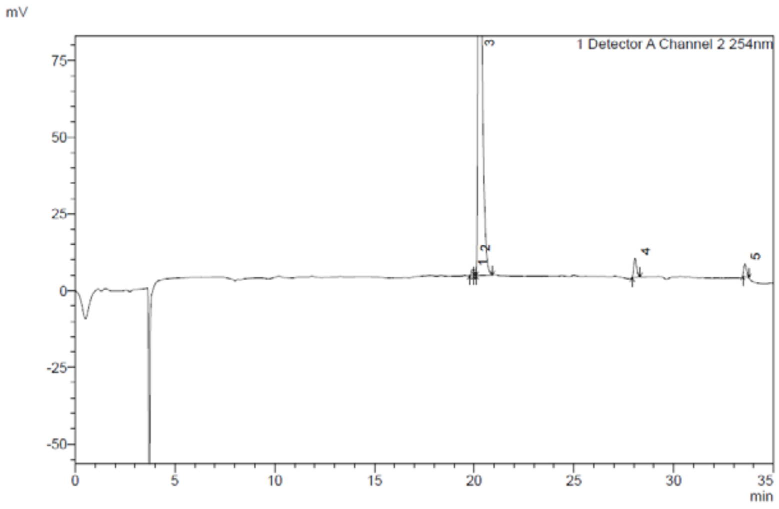

Detector A Channel 2 254nm

Peak#	Area	Area%
1	16683	0.529
2	12840	0.407
3	3024025	95.817
4	61016	1.933
5	41493	1.315
Total	3156057	100.000

^{1}H NMR hexa-2

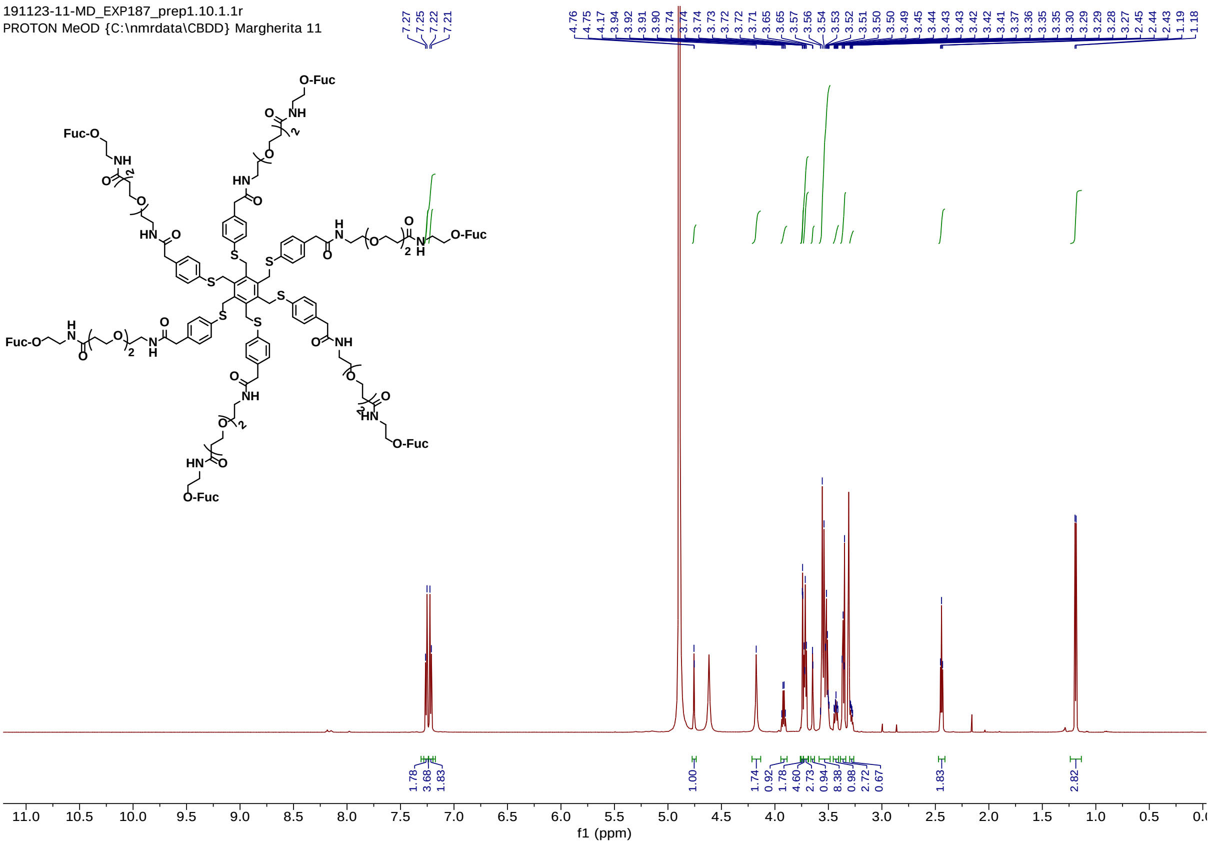

Analytical HPLC hexa-2 (blank subtracted)

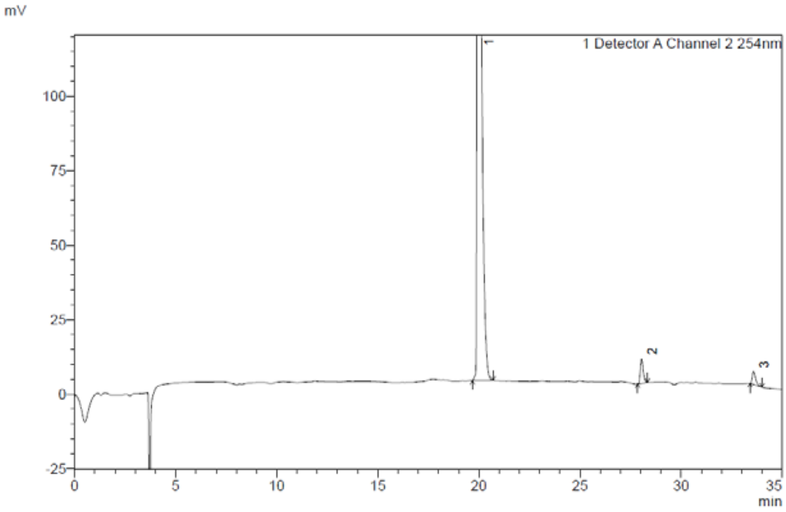

Detector A Channel 2 254nm

Peak#	Area	Area%
1	5603199	97.705
2	81076	1.414
3	50515	0.881
Total	5734789	100.000

^{1}H NMR tri-3

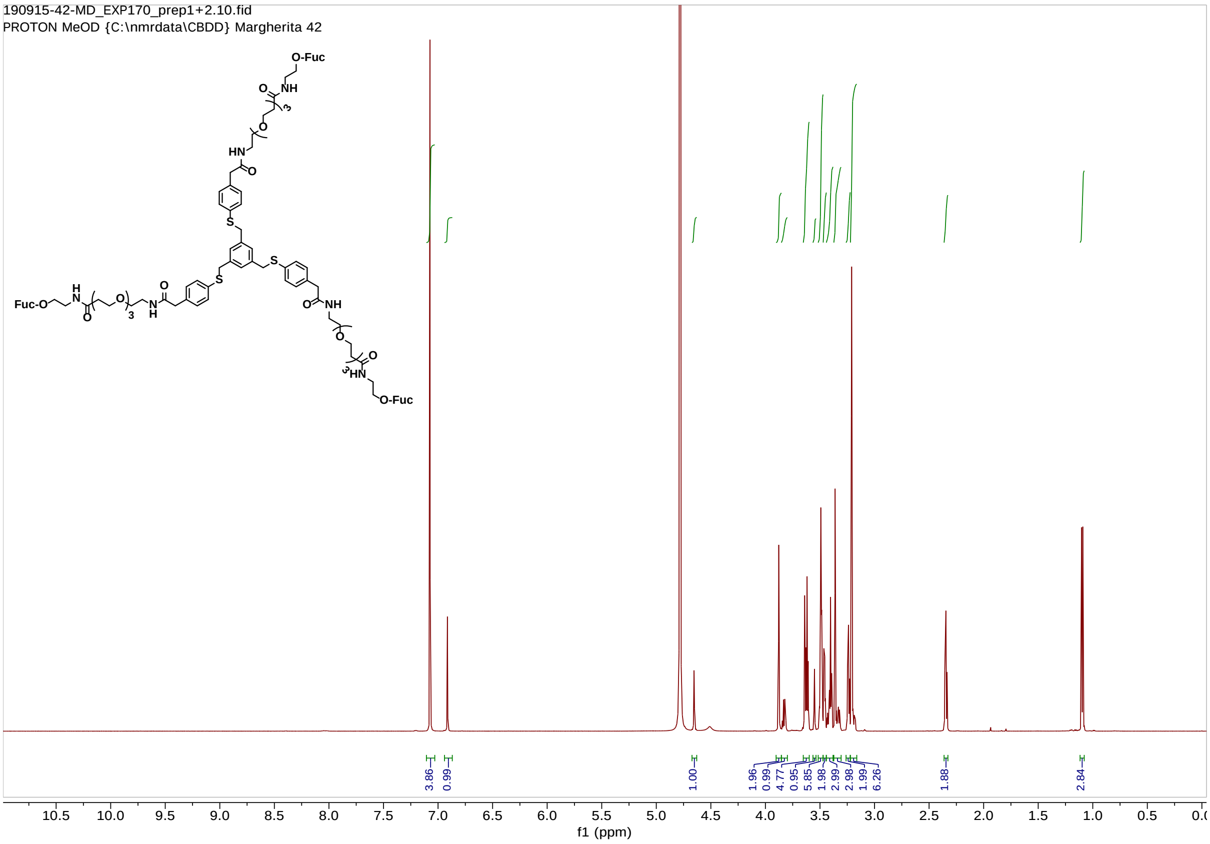

Analytical HPLC tri-3 (blank subtracted)

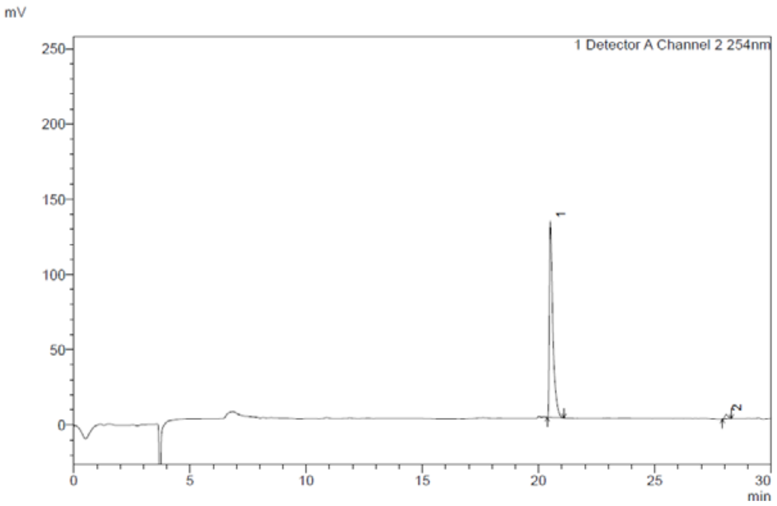

Detector A Channel 2 254nm

Peak#	Area	Area%
1	1375078	98.124
2	26286	1.876
Total	1401364	100.000

^{1}H NMR tetra-3

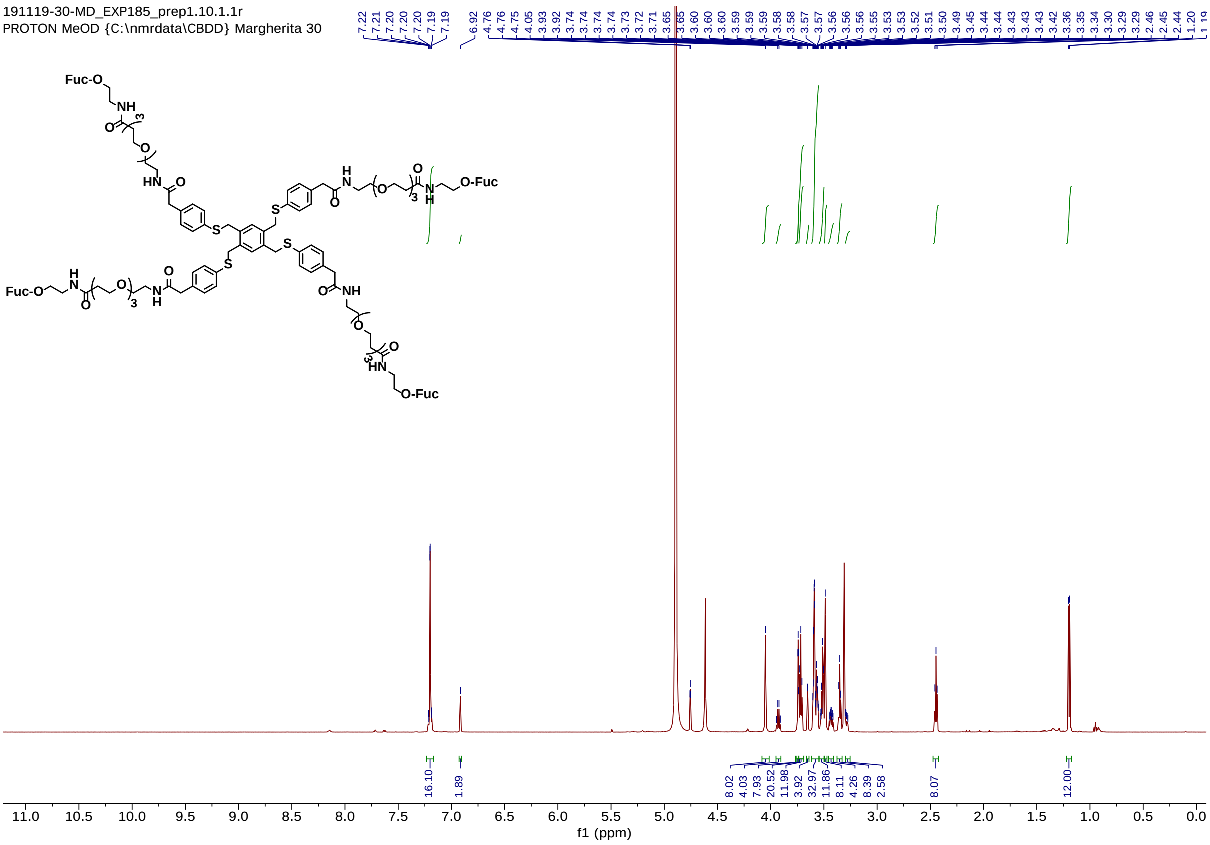

Analytical HPLC tetra-3 (blank subtracted)

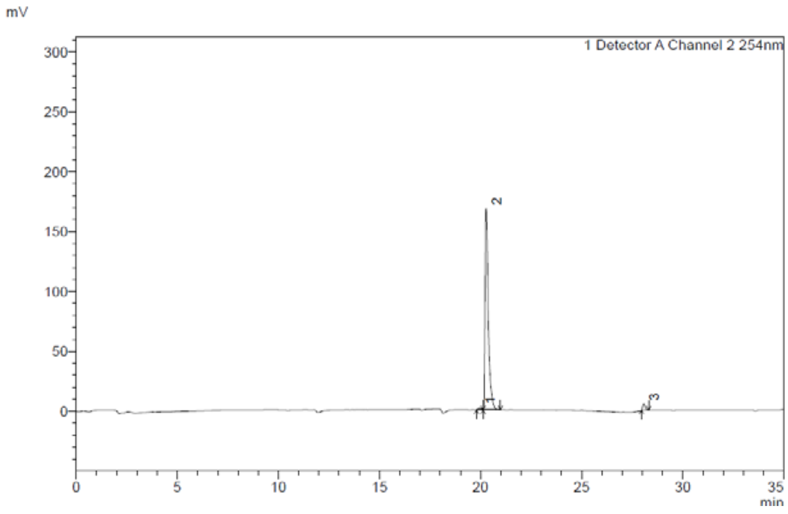

Detector A Channel 2 254nm

Peak#	Area	Area%
1	18304	0.945
2	1874208	96.793
3	43791	2.262
Total	1936303	100.000

^{1}H NMR hexa-3

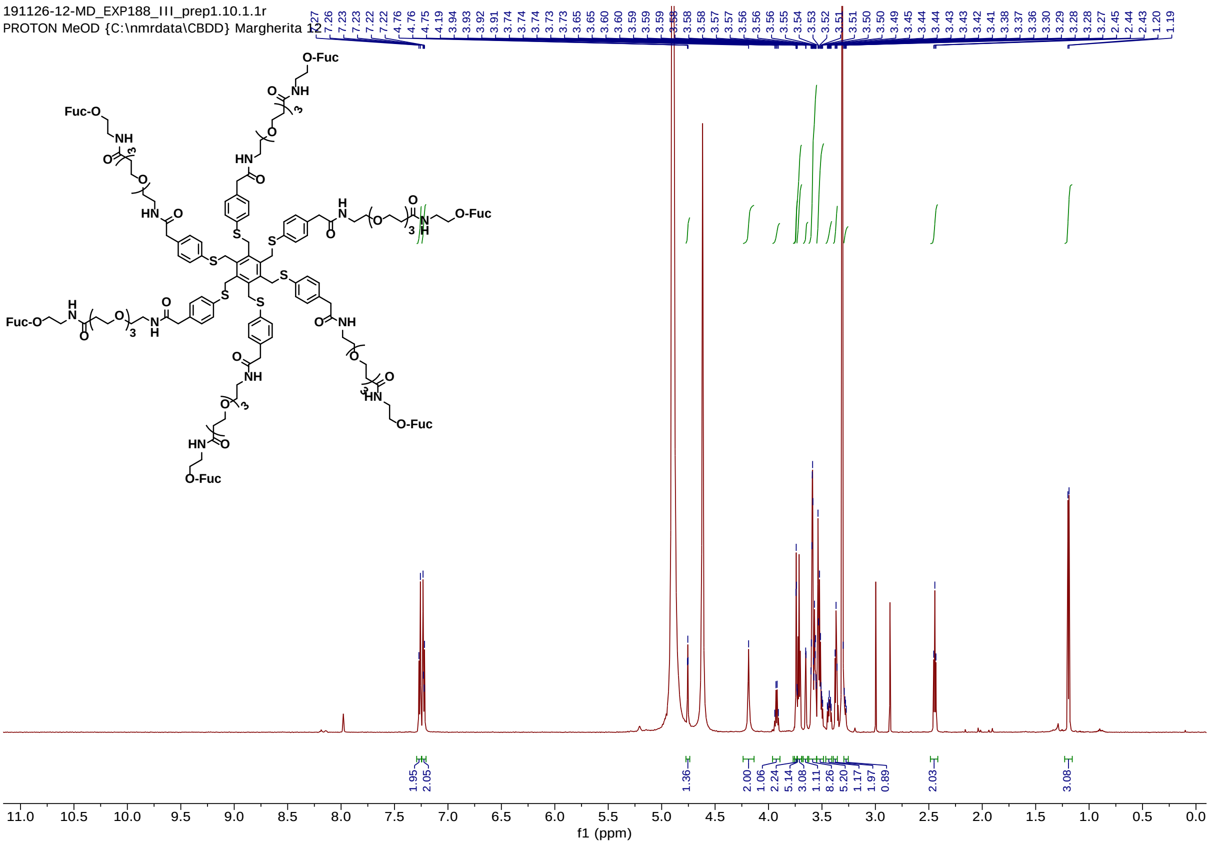

Analytical HPLC hexa-3 (blank subtracted)

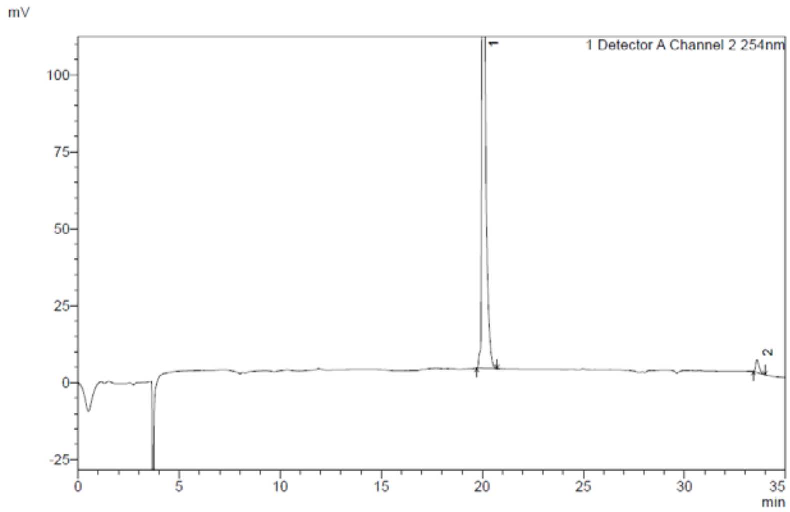

Detector A Channel 2 254nm

Peak#	Area	Area%
1	2721203	98.275
2	47768	1.725
Total	2768970	100.000

^{1}H NMR tri-4

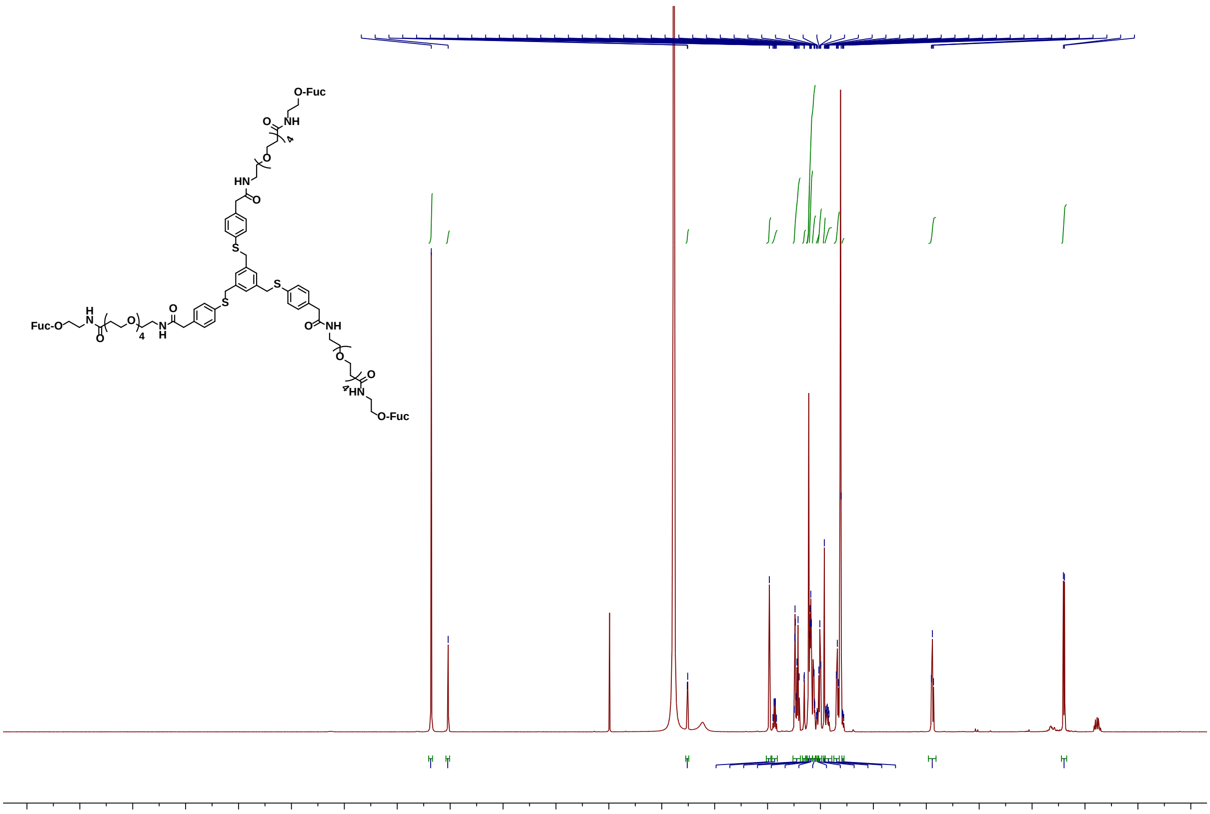

Analytical HPLC tri-4 (blank subtracted)

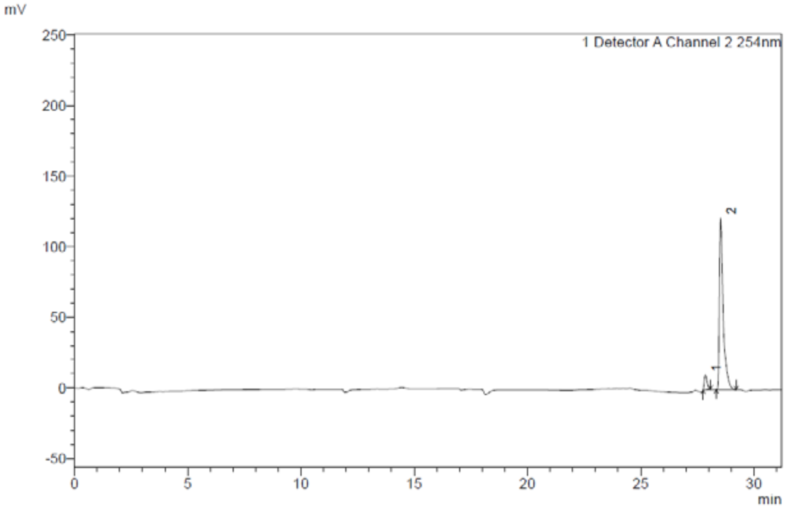

Detector A Channel 2 254nm

Peak#	Area	Area%
1	82035	5.401
2	1436720	94.599
Total	1518755	100.000

^{1}H NMR tetra-4

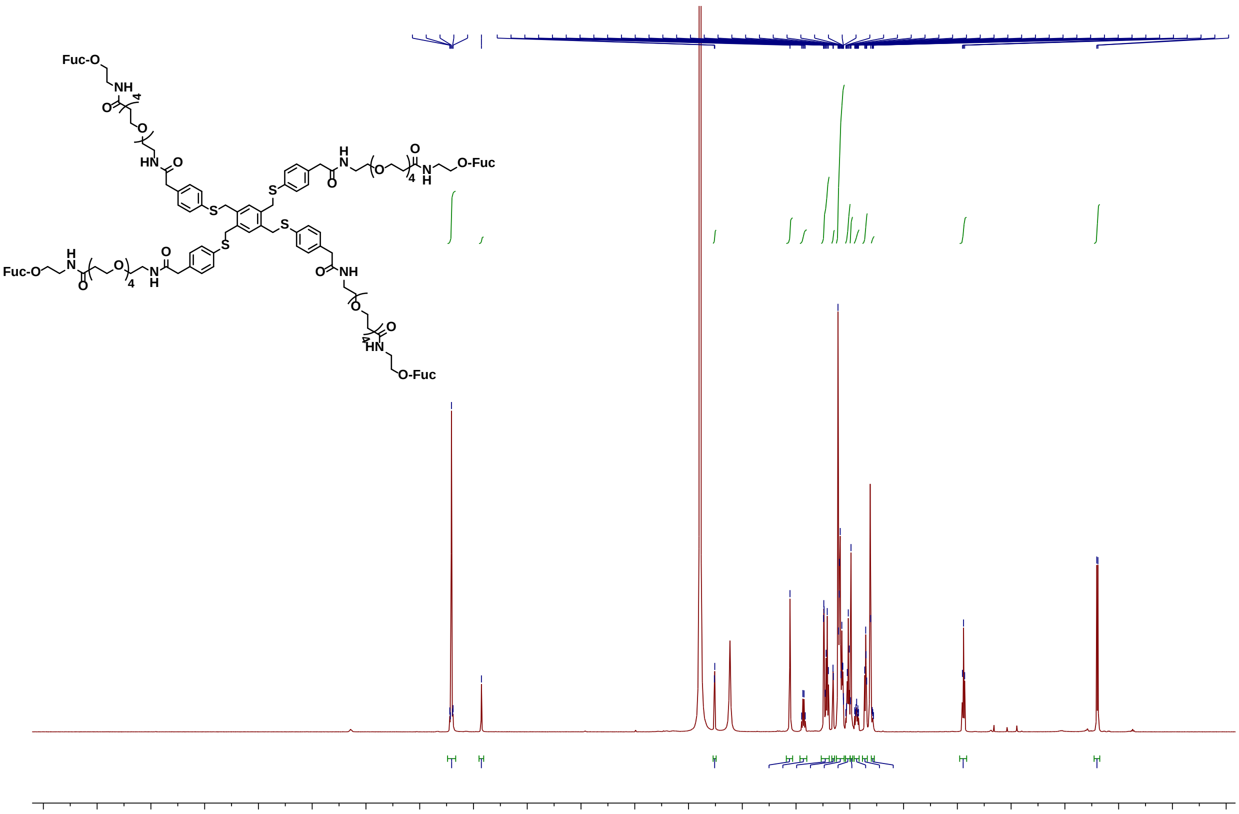

Analytical HPLC tetra-4 (blank subtracted)

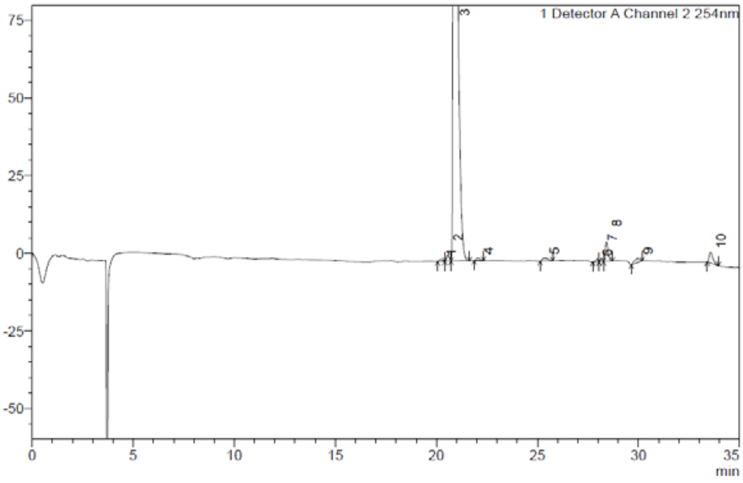

Detector A Channel 2 254nm

Peak#	Area	Area%
1	7346	0.145
2	21752	0.430
3	4852136	95.848
4	11651	0.230
5	20900	0.413
6	8125	0.161
7	6843	0.135
8	64575	1.276
9	23951	0.473
10	45045	0.890
Total	5062324	100.000

^{1}H NMR hexa-4

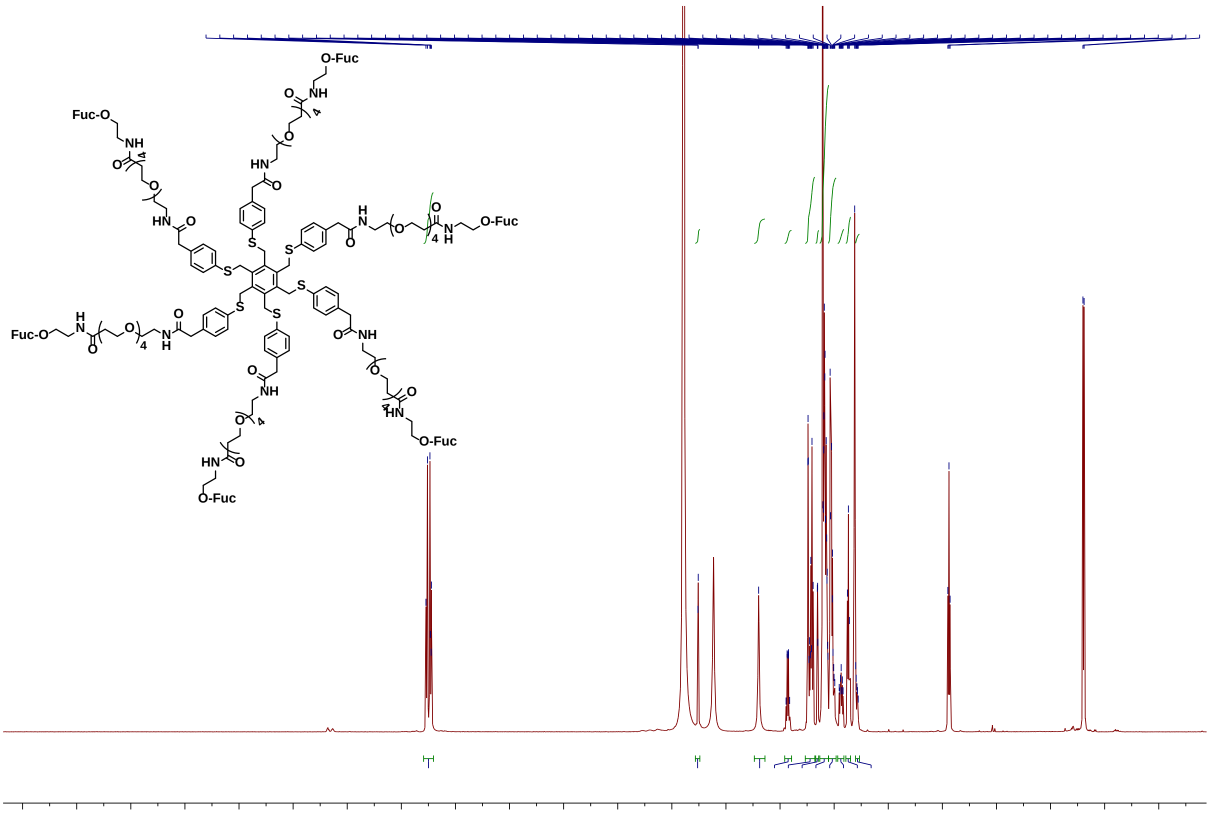

Analytical HPLC hexa-4 (blank subtracted)

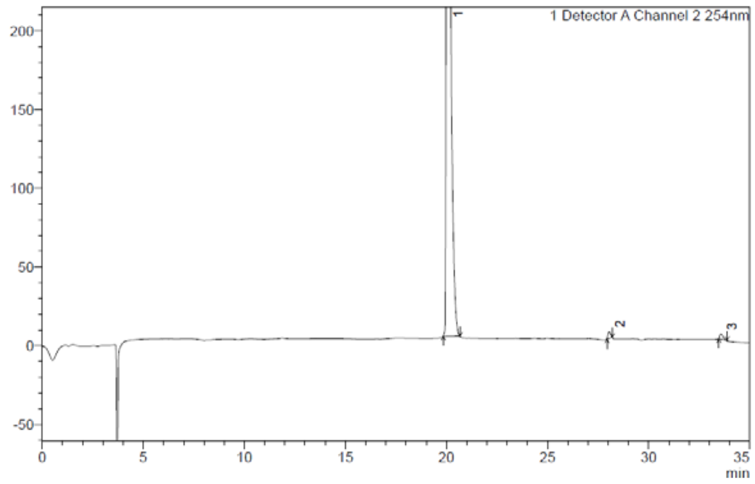

Detector A Channel 2 254nm

Peak#	Area	Area%
1	10680797	99.288
2	37690	0.350
3	38859	0.361
Total	10757347	100.000

¹H NMR hPG-20

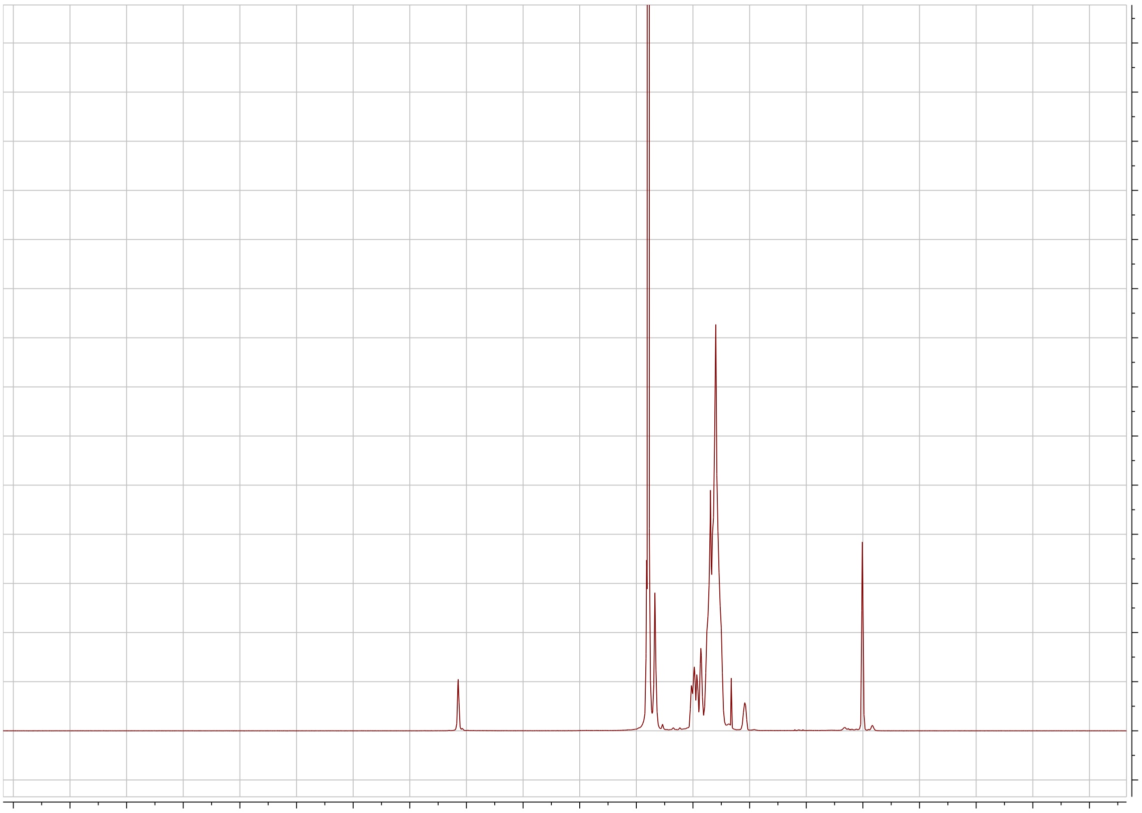

IR spectra hPG-20

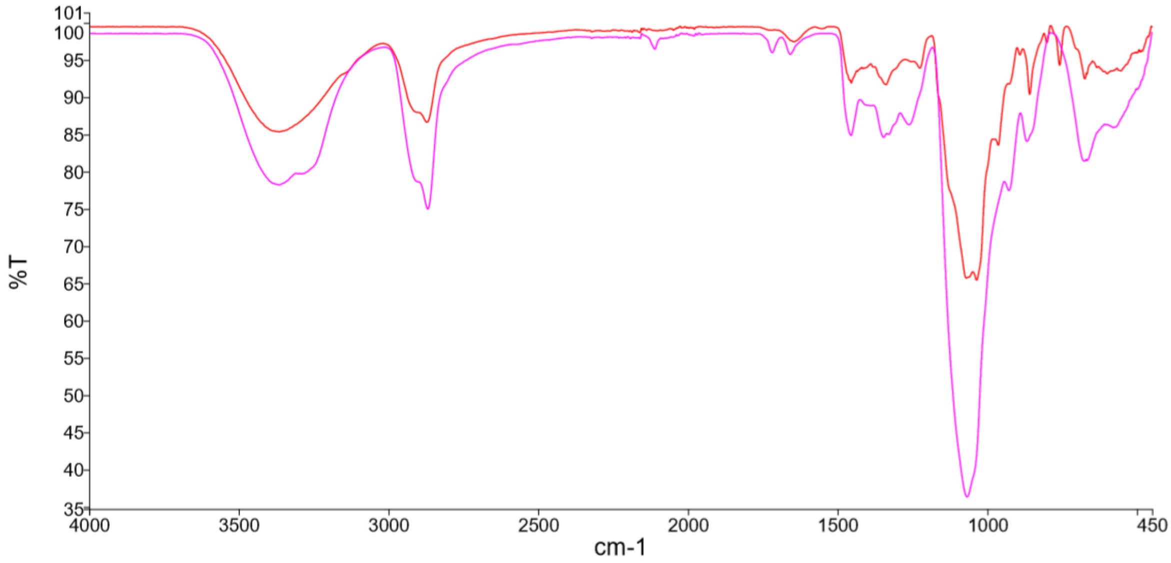

hPG-20; hPG-propargyl (alkyne)

8. HRMS traces

tri-2

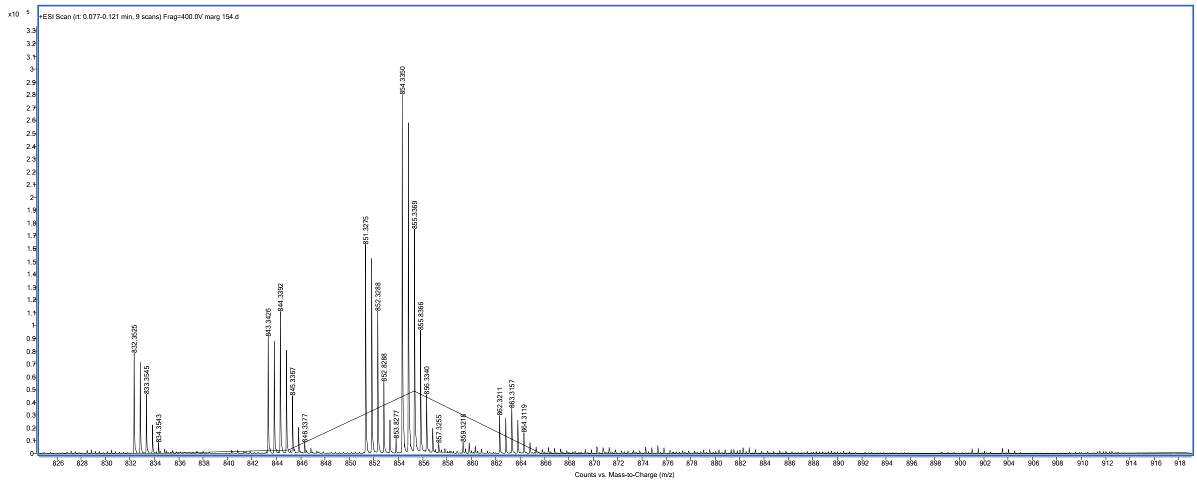

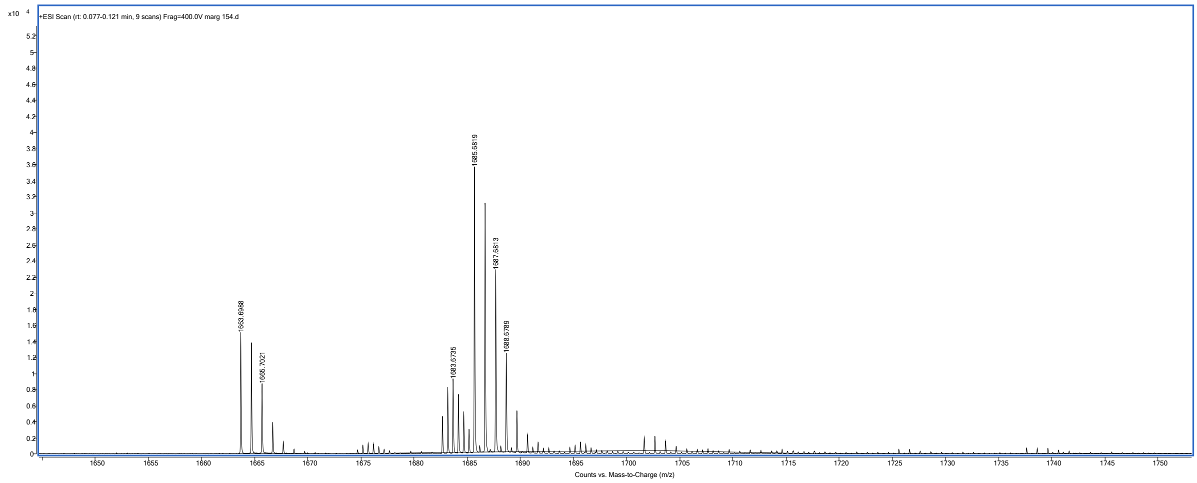

tri-3

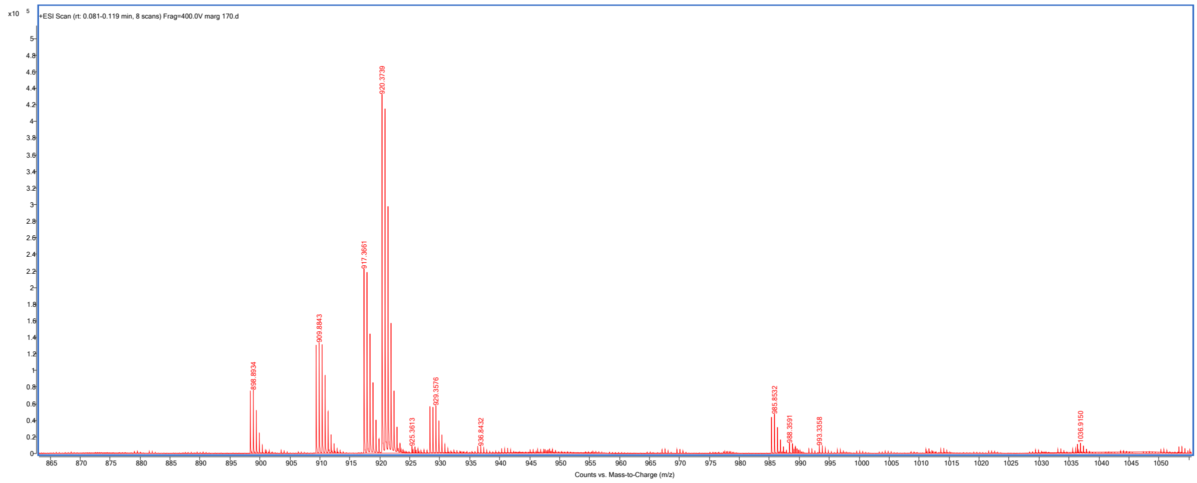

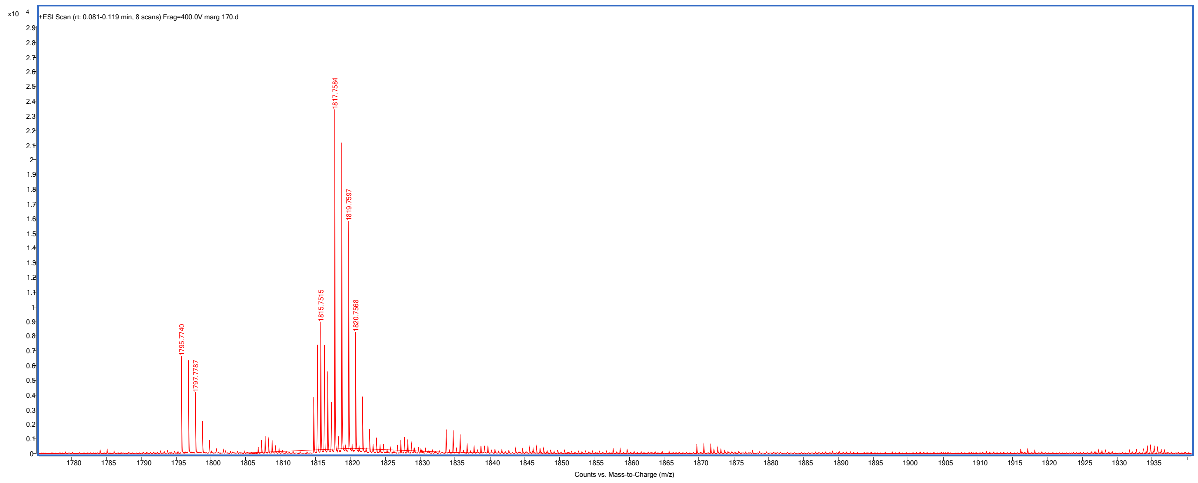
+ESI Scan (rt: 0.081-0.119 min, 8 scans) Frag=400.0V marg 170.d
1795.7740
1797.7787
1815.7515
1817.7584
1819.7597
1820.7568
Counts vs. Mass-to-Charge (m/z)

tri-4

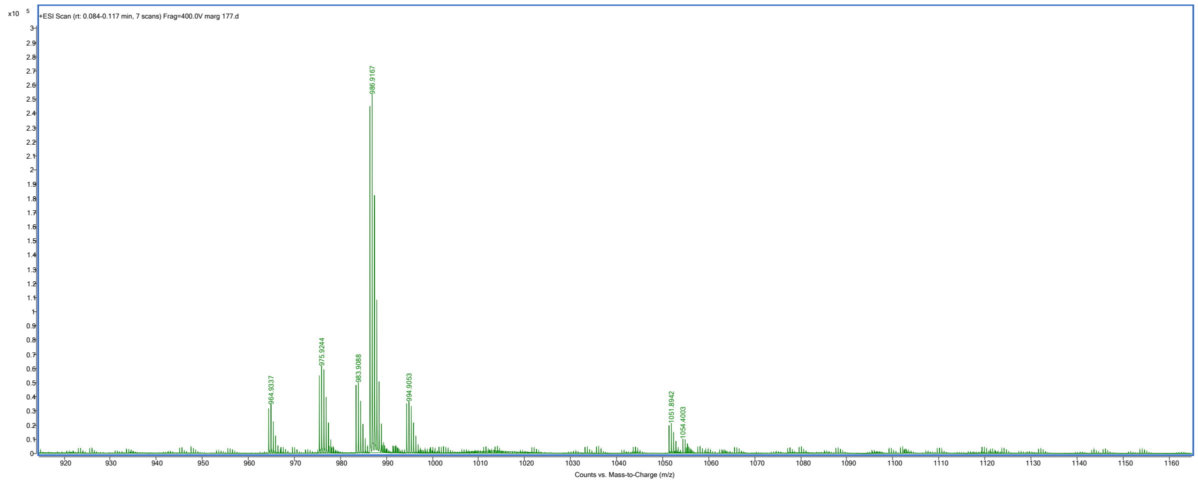
+ESI Scan (rt: 0.084-0.117 min, 7 scans) Frag=400.0V marg 177.d
964.9337
975.9244
983.9088
986.9167
994.9053
1051.8942
1054.4003
Counts vs. Mass-to-Charge (m/z)

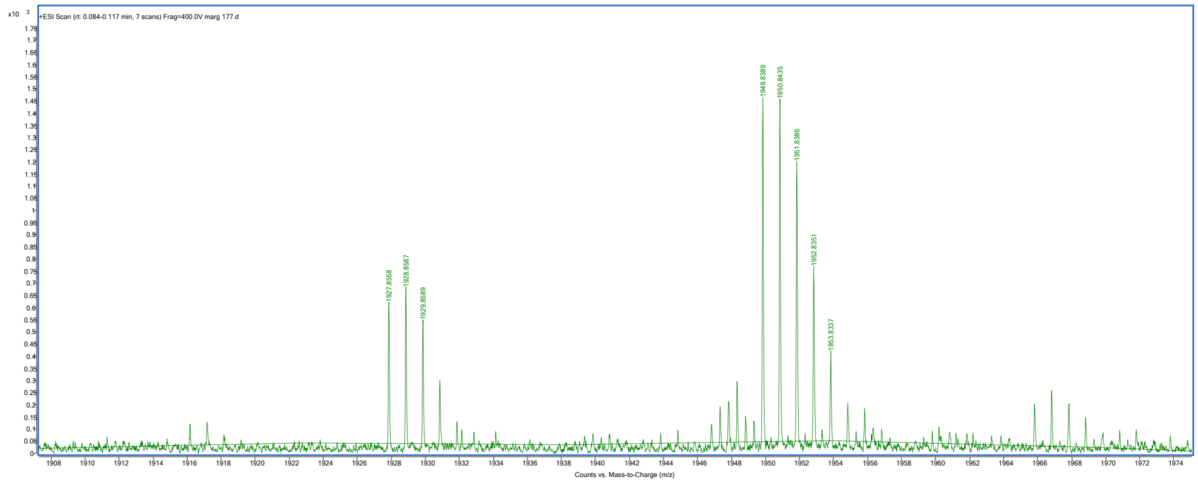
+ESI Scan (rt: 0.084-0.117 min, 7 scans) Frag=400.0V marg 177.d
1927.8558
1928.8587
1929.8589
1949.8389
1950.8435
1951.8386
1952.8351
1953.8337
Counts vs. Mass-to-Charge (m/z)

tetra-2

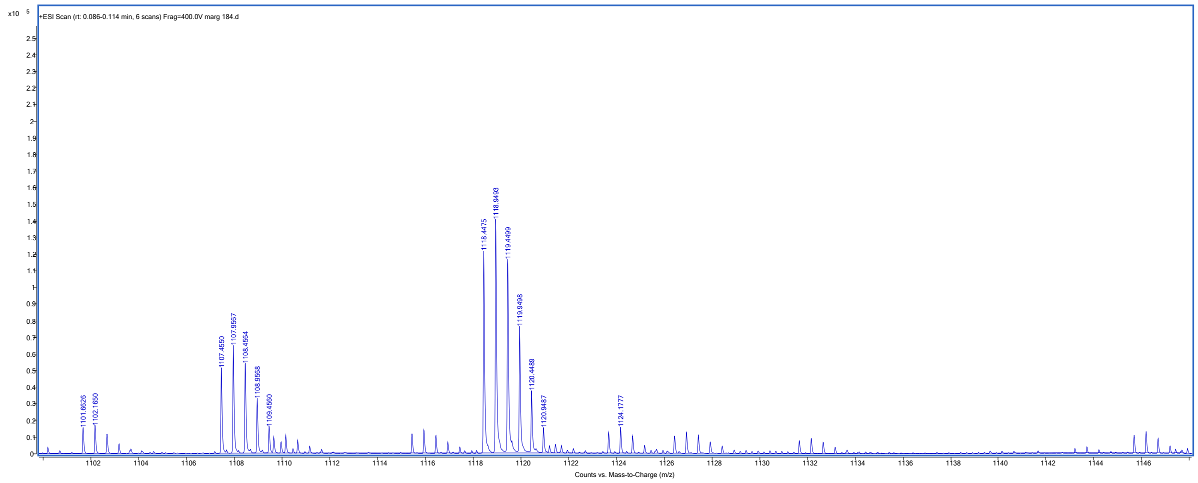

x10 5
+ESI Scan (rt: 0.086-0.114 min, 6 scans) Frag=400.0V marg 184.d
1101.6626
1102.1650
1107.4550
1107.9567
1108.4564
1108.9568
1109.4560
1118.4475
1118.9493
1119.4499
1119.9498
1120.4489
1120.9487
1124.1777
Counts vs. Mass-to-Charge (m/z)

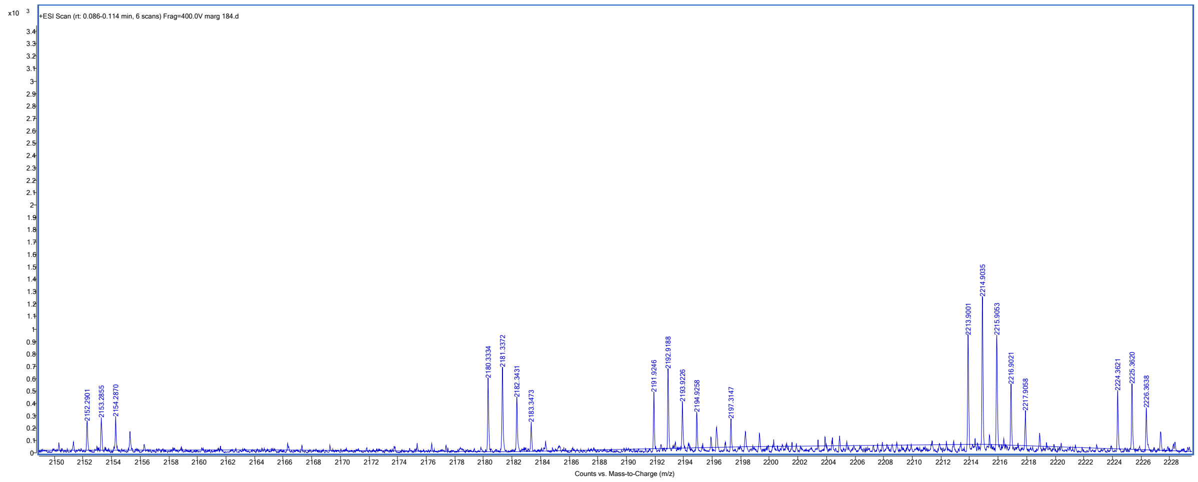

x10 3
+ESI Scan (rt: 0.086-0.114 min, 6 scans) Frag=400.0V marg 184.d
2152.2901
2153.2855
2154.2870
2180.3334
2181.3372
2182.3431
2183.3473
2191.9246
2192.9188
2193.9226
2194.9258
2197.3147
2213.9001
2214.9035
2215.9053
2216.9021
2217.9058
2224.3621
2225.3620
2226.3638
Counts vs. Mass-to-Charge (m/z)

tetra-3

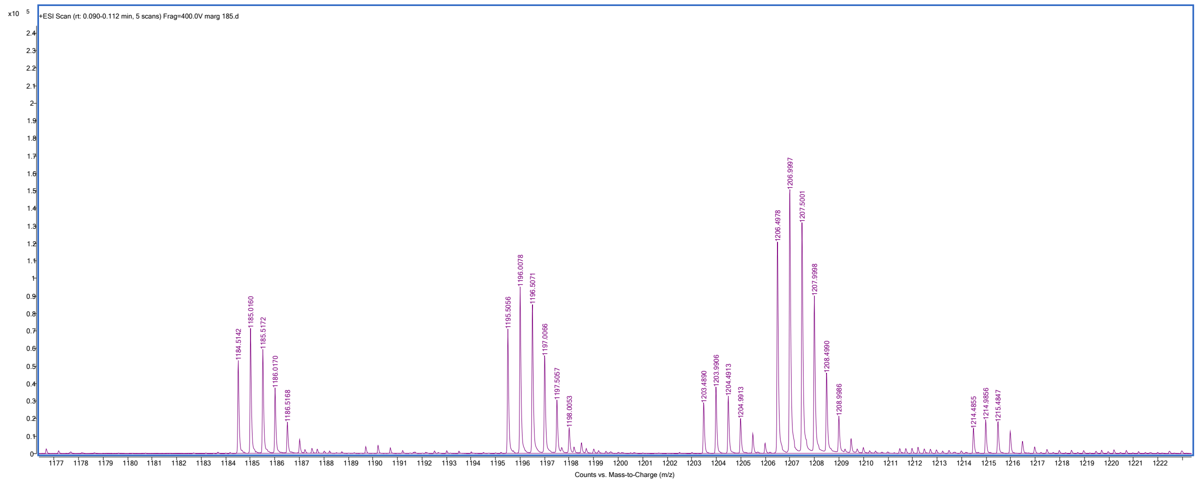

x10 5
+ESI Scan (rt: 0.090-0.112 min, 5 scans) Frag=400.0V marg 185.d
1184.5142
1185.0160
1185.5172
1186.0170
1186.5168
1195.5056
1196.0078
1196.5071
1197.0066
1197.5057
1198.0053
1203.4990
1203.9906
1204.4913
1204.9913
1206.4978
1206.9997
1207.5001
1207.9998
1208.4990
1208.9986
1214.4855
1214.9856
1215.4847
Counts vs. Mass-to-Charge (m/z)

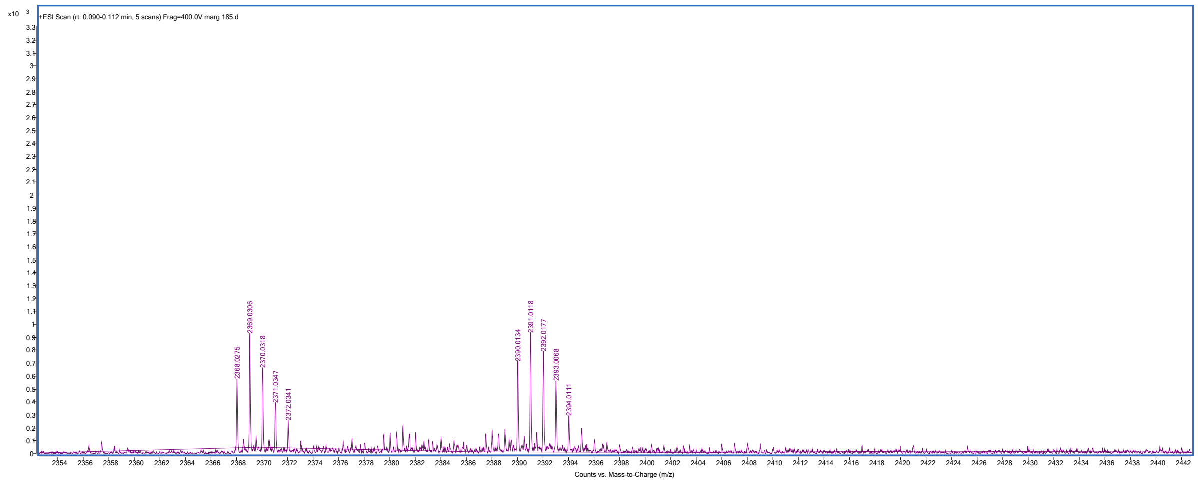

tetra-4

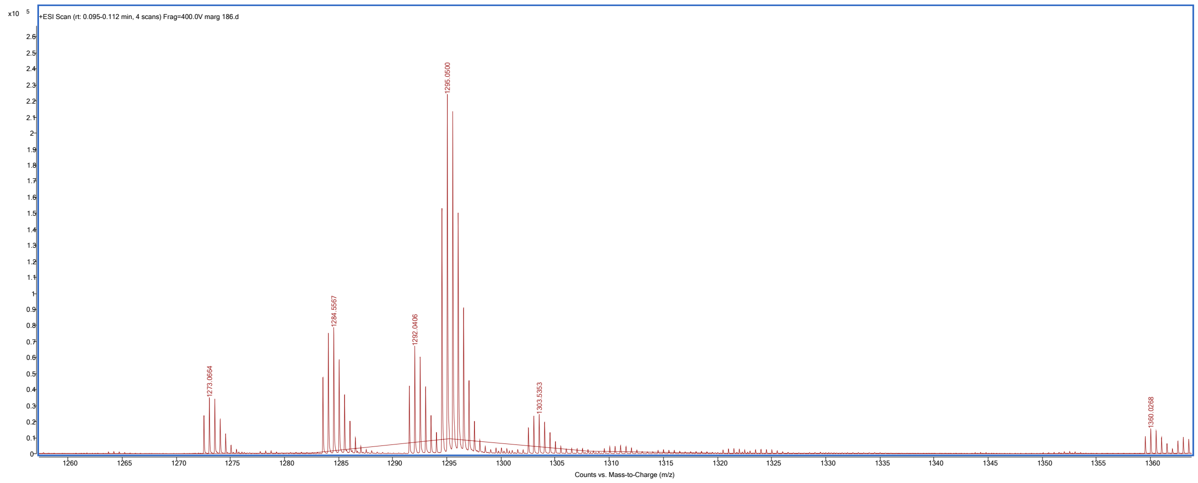

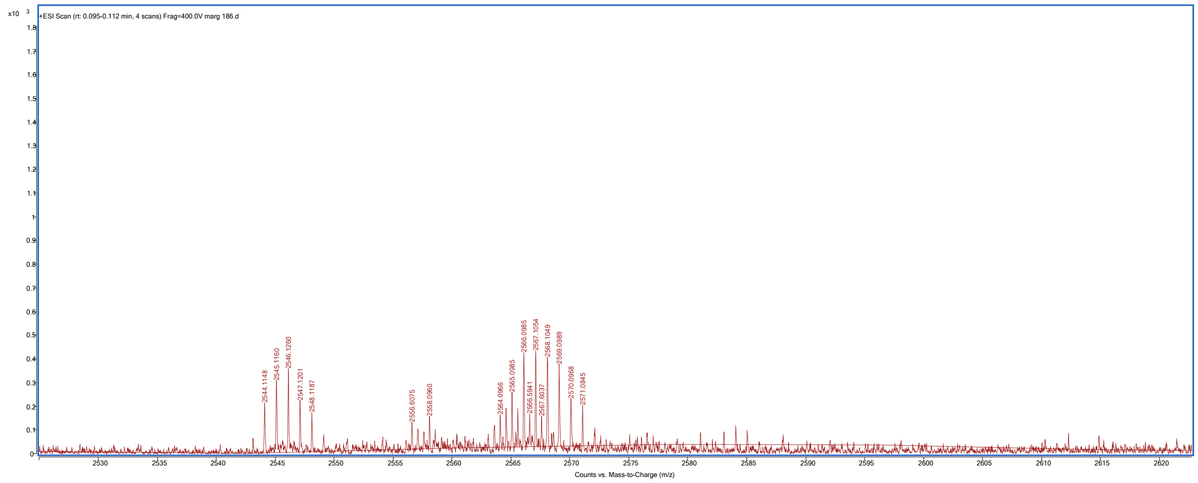

hexa-2

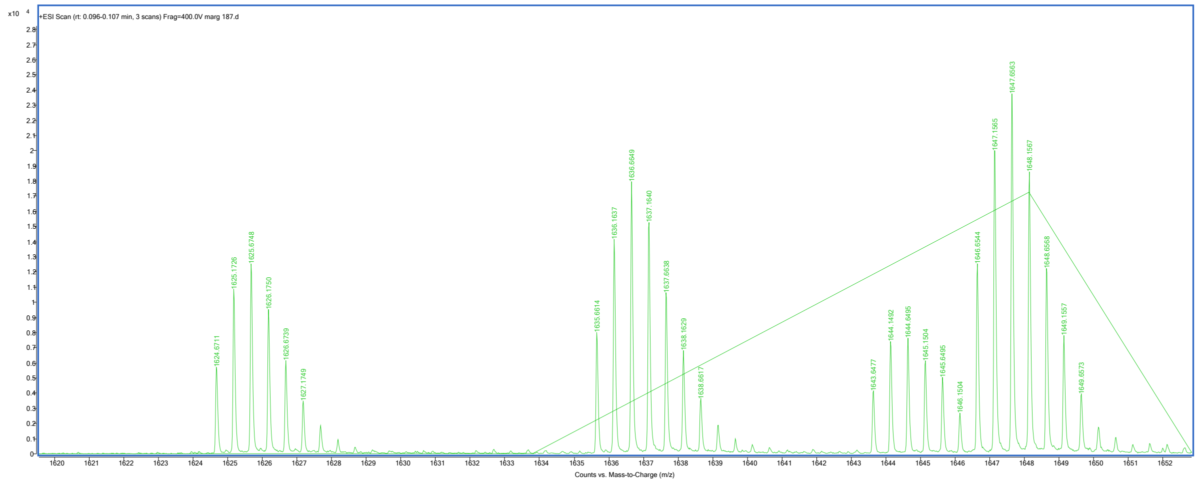
+ESI Scan (rt: 0.096-0.107 min, 3 scans) Frag=400.0V marg 187.d
Counts vs. Mass-to-Charge (m/z)

hexa-3

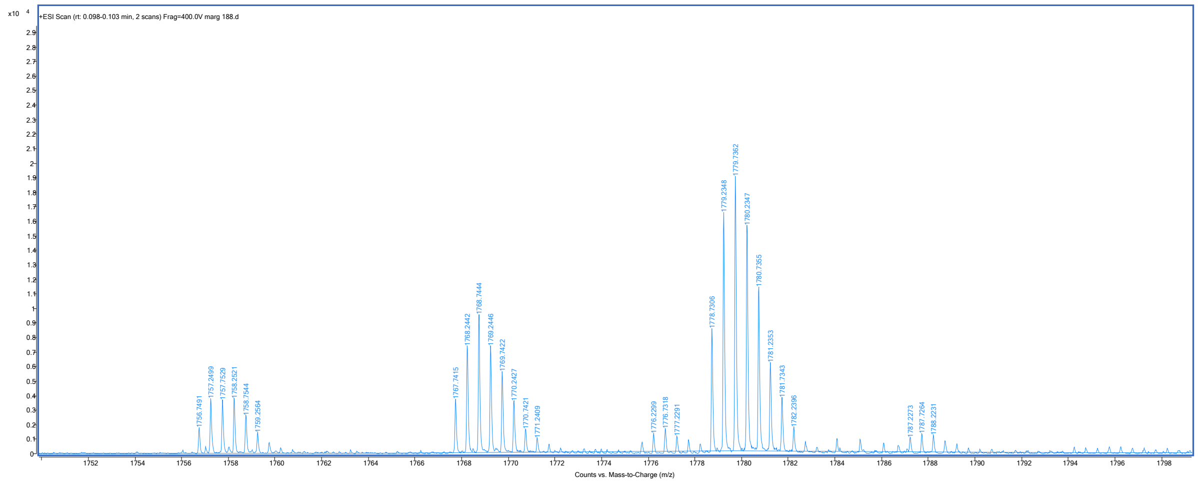
+ESI Scan (rt: 0.098-0.103 min, 2 scans) Frag=400.0V marg 188.d
Counts vs. Mass-to-Charge (m/z)

9. References

(1) Hauck, D.; Joachim, I.; Frommeyer, B.; Varrot, A.; Philipp, B.; Möller, H. M.; Imberty, A.; Exner, T. E.; Titz, A. Discovery of Two Classes of Potent Glycomimetic Inhibitors of *Pseudomonas aeruginosa* LecB with Distinct Binding Modes. *ACS Chem. Biol.* **2013**, No. 8, 1775–1784.

(2) Schuck, P. Size-Distribution Analysis of Macromolecules by Sedimentation Velocity Ultracentrifugation and Lamm Equation Modeling. *Biophys. J.* **2000**, *78* (3), 1606–1619.

(3) Escobar, N.; Ordonez, S. R.; Wösten, H. A. B.; Haas, P. J. A.; de Cock, H.; Haagsman, H. P. Hide, Keep Quiet, and Keep Low: Properties That Make *Aspergillus fumigatus* a Successful Lung Pathogen. *Front. Microbiol.* **2016**, 7 (APR), 1–13.

(4) Eissa, A. M.; Smith, M. J. P.; Kubilis, A.; Mosely, J. A.; Cameron, N. R. Polymersome-Forming Amphiphilic Glycosylated Polymers: Synthesis and Characterization. *J. Polym. Sci. Part A Polym. Chem.* **2013**, *51* (24), 5184–5193.
